# Neurotrophin System as a Complex Set of Potential Biomarkers in Psychiatric Disorders and Therapy—Overview of Recent Advances

**DOI:** 10.3390/ijms27146142

**Published:** 2026-07-09

**Authors:** Dragica Selakovic, Marina Mitrovic, Vladimir Janjic, Dragan Milovanovic, Nemanja Jovicic, Ermin Fetahovic, Jovan Milosavljevic, Gvozden Rosic

**Affiliations:** 1 Department of Physiology, Faculty of Medical Sciences, University of Kragujevac, 34000 Kragujevac, Serbia; jovan.milosavljevic1997@gmail.com (J.M.); grosic@fmn.kg.ac.rs (G.R.); 2 Department of Medical Biochemistry, Faculty of Medical Sciences, University of Kragujevac, 34000 Kragujevac, Serbia; mitrovicmarina34@gmail.com; 3 Center for Molecular Medicine and Stem Cell Research, Faculty of Medical Sciences, University of Kragujevac, 34000 Kragujevac, Serbia; 4 Department of Communication Skills, Ethics, and Psychology, Faculty of Medical Sciences, University of Kragujevac, 34000 Kragujevac, Serbia; vladadok@yahoo.com (V.J.); erminfetahovic96@gmail.com (E.F.); 5 Department of Psychiatry, Faculty of Medical Sciences, University of Kragujevac, 34000 Kragujevac, Serbia; 6 Psychiatric Clinic, University Clinical Center Kragujevac, 34000 Kragujevac, Serbia; 7 Department of Pharmacology and Toxicology, Faculty of Medical Sciences, University of Kragujevac, 34000 Kragujevac, Serbia; piki@fmn.kg.ac.rs; 8 Department of Histology and Embryology, Faculty of Medical Sciences, University of Kragujevac, 34000 Kragujevac, Serbia; nemanjajovicic.kg@gmail.com

**Keywords:** neurotrophins, neurotrophin receptors, psychiatric disorders, depression, anxiety, schizophrenia, bipolar disorder, autism, sleep disorders

## Abstract

The neurotrophin system has numerous regulatory roles in the central nervous system, including the growth, differentiation, and survival of neurons, the control of neurogenesis, synaptic plasticity, and the mediation of neurotransmitter balance, also with proven anti-inflammatory, anti-apoptotic, and antioxidative actions. These functions are seriously affected by numerous pathophysiological processes accompanied by mental illness. The variety of therapeutic protocols (pharmacotherapy, transcranial stimulation, psychotherapy, lifestyle changes, programmed physical activity, dietary interventions), individually or in combination, has been employed in the treatment of psychiatric disorders. To evaluate the effectiveness of therapeutic strategies, specific elements of the neurotrophin system have been quantified to assess their potential as biomarkers. However, data obtained in clinical trials for different psychiatric disorders still do not confirm the reliability of neurotrophin system elements as biomarkers. For this purpose, we aimed this narrative review to explore clinical studies published in the last decade that examined neurotrophin system elements in patients with various psychiatric disorders, with a focus on their association with disease severity, clinical state, and treatment response, which represents a promising avenue for improving our understanding of the underlying mechanisms and for identifying potential clinically relevant biomarkers, as well as sharpening therapeutic protocol outcome analyses.

## 1. Introduction

Mental disorders are increasingly recognized as leading causes of disease burden and remain among the top ten leading causes of burden worldwide, with no evidence of global reduction [1,2]. To reduce the burden of mental disorders and coordinate the delivery of effective prevention and treatment programs, clinicians are recommended to follow the international classifications (ICD-11 and DSM-5) to allow more effective communication and to establish a convenient shorthand for describing mental disorders, as well as to facilitate the identification and management of mental disorders in clinical settings [3].

Psychiatric disorders arise from a dynamic interplay of modifiable and non-modifiable factors, with neurotrophins representing a central molecular position through which diverse influences converge to shape both brain plasticity and vulnerability to mental illness. Despite the heterogeneity of diseases and symptoms, some common key pathophysiological mechanisms have been identified. Thus, neurotransmitter imbalance (monoamine deficiencies in depression, dopaminergic signaling and glutamate/GABA dysfunction in schizophrenia [4]), neuromorphological aberrations (decreased neuroplasticity in depression [5]), neuroendocrine dysregulation (in mood disorders [6]), neuroinflammatory processes (irregularity of synaptic transmission in depression and schizophrenia [7,8]), as well as specific neural circuit dysfunctions (in affective disorders [9]), have been attributed to the pathophysiological background of psychiatric disorders. All of them may be connected to various factors (genetics, environmental factors) contributing to triggering and developing mental illnesses [10,11]. Since the same etiology has been confirmed in a variety of neurodegenerative disorders, it is not surprising that neurodegenerative and psychiatric disorders show significant comorbidity [12]. Finally, it is of substantial importance that the neurotrophin system is concomitantly affected by the same triggering factors and that its disturbance is involved in all pathophysiological events, giving it a central role in the mediation and genesis of various psychiatric disorders.

**Figure 1:**
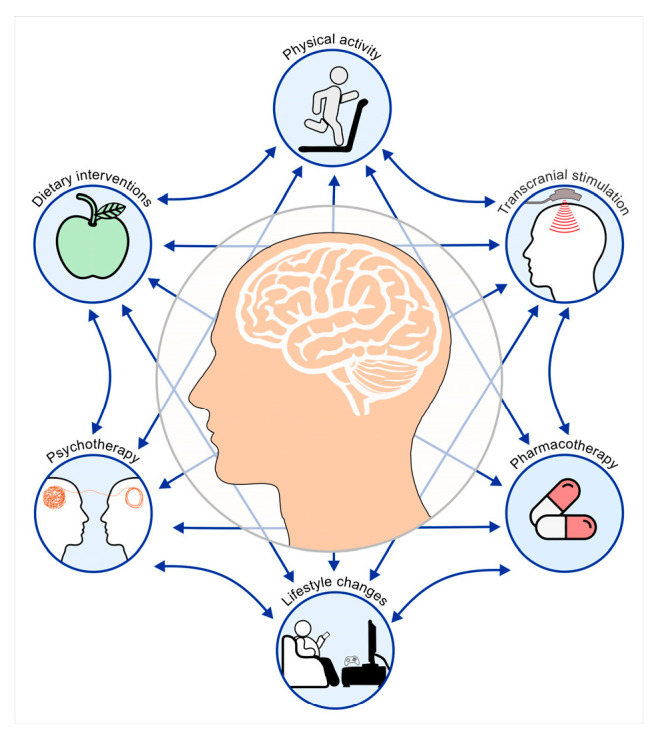
Various therapeutic approaches in treating psychiatric disorders.

As illustrated in Figure 1, a wide spectrum of interventions, including physical activity, dietary patterns, psychotherapy, pharmacotherapy, transcranial stimulation, and broader lifestyle factors, can bidirectionally modulate neurotrophin signaling, thereby influencing synaptic integrity, neuronal survival and resilience, and circuit reorganization. These factors form a connected and adaptive system, where behavioral, environmental, and therapeutic influences interact to either increase the risk of, or help protect against, the development and progression of psychiatric disorders. All mentioned interventions can act as risk-enhancing or protective determinants depending on their intensity, duration, and interaction with individual biological susceptibility. This integrative perspective highlights the necessity of multimodal strategies that simultaneously target lifestyle, psychological, and neurobiological domains to effectively prevent and treat mental illness.

Despite major advances in psychiatric research, truly reliable biological markers for everyday clinical use for diagnosis, prognosis, or treatment response are still missing. Neurotrophins are therefore no longer seen as isolated molecules, but as part of a broader, highly dynamic system. This system is deeply involved in synaptic plasticity, stress responses, and the ability of the brain to adapt to different conditions. At the same time, findings in this area are far from uniform. Changes in the neurotrophin system do not follow a single pattern, and they are not specific to one disorder. Instead, they vary across psychiatric conditions and can even point in opposite directions. For that reason, their role as clear diagnostic markers remains limited and still speculative. However, this variability also makes them interesting from a biological perspective, as it may reflect underlying mechanisms of mental disorders and open space for new treatment approaches. Psychiatric research has been shifting toward markers that go beyond traditional diagnostic categories and better capture core disease processes. In that sense, the neurotrophin system stands out as a strong candidate, given its central role in neuroplasticity and in the brain response to both external and internal stress, and to therapeutic procedures.

This narrative review aimed to explore clinical studies published in the last decade that examined neurotrophin system elements in patients with various psychiatric disorders, with a focus on their association with disease severity, clinical state, and treatment response, which represents a promising avenue for improving understanding of underlying mechanisms and identifying potential clinically relevant biomarkers, as well as sharpening therapeutic protocols’ outcome analyses.

## 2. Literature Search Strategy

A literature search was conducted on PubMed covering studies published in the last decade. The search approach employed Boolean operators to link the keywords, such as “anxiety” OR “depression”, etc. (for specific psychiatric disorder) AND “neurotrophins” OR “neurotrophin receptors”, etc. (for specific neurotrophin system elements), and included only the full-text original scientific articles, obtained in clinical trials, available in English on the PubMed database (by 8 May 2026). Review articles, conference abstracts, editorials, and studies unrelated to neurotrophin system elements and/or specific psychiatric disorders were excluded. In addition to the data that met the inclusion criteria (presented in the Tables), additional articles were identified by screening the reference lists of the selected studies and relevant reviews. As the most important limitation of this narrative review, we acknowledge that restricting the review to the last decade may have excluded some earlier studies (with all respect to the high-quality studies and some very influential pioneering investigations). However, this approach allowed us to concentrate on recent developments and emerging findings that appeared along with improved methodological approaches (pharmacological and other treatment protocols) and significantly increased the number of neurotrophin system elements to be evaluated (including the interactions between various neurotrophin system elements and their potential clinical impact, which can be important for potential exploratory/biomarker use). Our objective was to focus on the most recent advances in this rapidly evolving field and to provide an up-to-date overview of contemporary evidence. Future reviews could specifically focus on a more detailed analysis of the entire body of literature and include all studies addressing both neurotrophin system elements and psychiatric disorders, regardless of publication date. Finally, regarding the inclusion of studies involving conditions other than primary psychiatric disorders, our intention was to provide a comprehensive overview of the neurotrophin system in the context of psychiatric disorders, while also considering evidence from studies investigating psychiatric symptoms and psychiatric manifestations (such as depressive or anxiety episodes) developing in the setting of other diseases (diabetes mellitus), developmental stages (aging), physiological states (pregnancy and delivery), and medical interventions (surgical procedures). We believe that these studies contribute important insights into the role of the neurotrophin system and help provide a broader understanding of the field. Therefore, we consider their inclusion important for presenting a comprehensive overview of the current state of knowledge.

## 3. Challenges in the Interpretation of Neurotrophin System Actions in the Brain

The neurotrophin system elements are essential for the growth, differentiation, and survival of neurons and significantly influence the control of neurogenesis, synaptic plasticity, and the pathogenesis of neurological and psychiatric disorders [13,14]. However, interpreting neurotrophins’ actions in the brain has become particularly challenging due to the underlying complexity of the neurotrophin system across several organizational levels.

### 3.1. Neurotrophin System Elements

**Figure 2:**
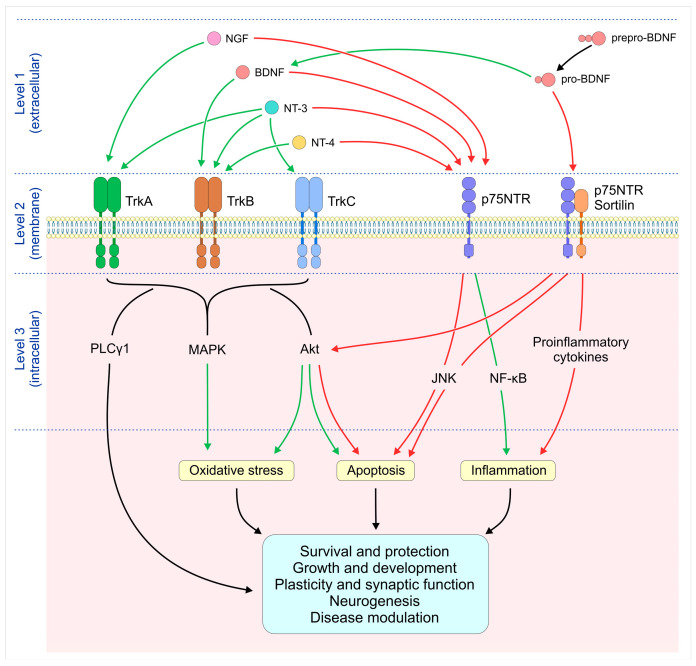
“Conflicting areas” that define the overall effect of neurotrophin system actions (green arrows denote stimulatory effect, red arrows denote inhibitory effect).

The mammalian neurotrophin system consists of four primary secreted proteins, including NGF, BDNF, NT-3, and NT-4/5. Neurotrophins, both mature and unprocessed pro-forms, interact and exert biological effects through two distinct receptor classes: the tropomyosin receptor kinase (Trk) family (TrkA for NGF and NT-3, TrkB for BDNF and NT-3/4, and TrkC for NT-3), which primarily mediates survival and growth-promoting effects, and the p75 neurotrophin receptor (p75NTR), a member of the TNF receptor superfamily that frequently mediates apoptosis or growth inhibition. Consequently, the neurotrophin system elements and their receptors (Trk and p75NTR) form a complex regulatory network that maintains equilibrium between axonal growth and synaptic pruning, as well as between neuronal survival and programmed cell death [15,16]. The complexity of the neurotrophin system poses a significant challenge in neuroscience, particularly in interpreting its actions and resulting clinical trial inconsistencies. To understand these inconsistencies, one must examine the “conflicting zones” at different neurotrophin system levels: extracellular (Level 1), cell membrane (Level 2), and downstream signaling (Level 3), as shown in Figure 2. Additionally, relying on a single neurotrophin as a biomarker overlooks important interactions and ratios that influence the neurotrophins’s functional outcomes.

Although not formally classified as a neurotrophin network element, GDNF (which belongs to the TGF-β superfamily) operates along with neurotrophic factors and significantly modulates the effects of neurotrophins, including BDNF. An interesting interplay was confirmed between BDNF and GDNF that actively interact, providing supportive feedback and maintaining brain tissue equilibrium [17]. This relationship was also documented in psychiatric disorders, where both BDNF and GDNF serum levels were proposed as potential biomarkers of generalized anxiety disorder [18].

### 3.2. Challenging Factors to Determine the Total Impact of the Neurotrophin System in the Brain

#### 3.2.1. Extracellular Compartment (Level 1): Conflicts in Proteolytic Processing–Pro- vs. Mature Neurotrophins

The first level of conflict associated with the neurotrophin system occurs at its origin. In general, all neurotrophins undergo a series of complex multistages during their synthesis and maturation [19]. Neurotrophins, such as BDNF, are first synthesized as pre-pro-BDNF, which is then further modified in the Golgi apparatus to produce pro-BDNF, which contains both a pro- and a mature domain, illustrating the complex process of obtaining fully functioning BDNF [20]. Importantly, pro-neurotrophins are effective mediators that influence biological actions (apoptosis, synaptic pruning), contrasting with mature neurotrophins that promote survival and plasticity. They primarily interact with p75NTR-sortilin receptors, while mature neurotrophins are more likely to interact with Trk receptors [21]. The challenge in the interpretation of neurotrophins action at the first level occurs in the extracellular space, which is influenced by a proteolytic environment and the distribution of neurotrophins. The pro-BDNF/BDNF ratio in this cellular compartment is crucial for neuronal outcomes, and its conversion is regulated by various proteases, such as intracellular furin/proprotein convertases and extracellular tPA/plasmin or matrix metalloproteinases (MMPs 2-9) [22]. Thus, the proteolytic cleavage of pro-neurotrophins into mature neurotrophins may act as a molecular switch between life and death signals. Indeed, many studies have suggested that an imbalance or inadequate pro-BDNF proteolytic conversion into BDNF may be crucial in the pathogenesis of neuropsychiatric disorders by affecting neural plasticity [23]. Some research demonstrates that pro-BDNF adversely affects mood regulation in depression, whereas mBDNF overexpression can mitigate the effects of pro-BDNF, suggesting that equilibrium between mBDNF and pro-BDNF is crucial for brain homeostasis and the prevention of depressive behavior [24]. The “conflict” arises from changes in the proteolytic environment during chronic inflammation or neurodegeneration, which is characterized by the downregulation of plasminogen activators or elevated levels of plasminogen activator inhibitor-1 (PAI-1), resulting in pro-BDNF accumulation and an increased PAI-1/BDNF ratio. Pro-BDNF induces long-term depression and apoptosis, whereas BDNF promotes long-term potentiation (LTP) and cellular survival [25,26]. Numerous studies have indicated that the altered expression and/or activities of components in the tPA/plasmin system result in an imbalance or inadequate conversion of pro-BDNF into BDNF, which is implicated in the pathological processes associated with depression, anxiety, and schizophrenia [21]. Moreover, an earlier study showed that the sorting of pro-NT in the extracellular compartment also involves sortilin, a member of the Vps10p receptor family, which acts as a molecular chaperone for pro-neurotrophin. The conflict here is structural: sortilin can either facilitate the trafficking of neurotrophin to the regulated secretory pathway [27] or, when present on the cell surface, act as a co-receptor for p75NTR to form a high-affinity death-signaling complex for pro-neurotrophin [28]. This dual role makes sortilin a critical player in whether a neurotrophin synthesis event results in trophic support or programmed cell death. Therefore, the pro- to mature neurotrophin ratio in the extracellular milieu becomes an important factor determining the functional outcome, although such ratios are rarely assessed in clinical trials.

#### 3.2.2. Cell Membrane (Level 2): Trk and p75NTR Receptor Crosstalk and Competition

In the second level of conflict, which takes place at the cell membrane, different neurotrophin receptors and co-receptors engage in cooperative or competitive activities, impacting the process by which extracellular neurotrophin signals trigger intracellular responses. The neurotrophin system elements can bind to two different types of receptors, Trk and p75NTR, that frequently compete for the same neurotrophin, which causes biochemical conflict at the cell membrane [29]. Trk receptors (TrkA, TrkB, and TrkC) operate in a dimeric fashion, and the binding of neurotrophin causes conformational changes that lead to autophosphorylation and downstream signaling, resulting mainly in neurogenesis and synaptic plasticity. However, the composition and ratios of Trk receptor assemblies may vary, as they can form both homodimers and sometimes heterodimers that include other Trk receptors, leading to alterations in signaling specificity [30]. Additionally, Trk receptors exhibit versatile actions due to allosteric regulation, where agonists bind to alternative sites to modify receptor functions, and ligand biasing, which allows different neurotrophins to trigger unique signaling pathways through the same Trk receptor [31].

The most significant challenge at the cellular membrane level presents the ratio of Trk to p75NTR, because the expression of p75NTR greatly affects Trk receptor signaling activity [32]. When p75NTR co-expresses with Trk receptors, it increases the binding affinity of neurotrophins to Trk receptors (e.g., NGF-TrkA) and functions as a co-receptor that enhances survival signaling [33]. On the other hand, the initial binding of BDNF to TrkB occurs independently of p75NTR, which only interacts with TrkB following phosphorylation, indicating that the formation of the TrkB/p75NTR complex is not required for BDNF binding; however, it is essential for BDNF/TrkB signaling [34]. Nonetheless, when p75NTR is expressed in the absence of Trk receptors or at low levels of Trk expression, or when it is activated by pro-neurotrophin in a complex with sortilin, it can initiate proapoptotic signaling [35]. Indeed, in various regions of the brain, including the hippocampus, the ratio of TrkB to p75NTR dictates whether BDNF signaling results in synaptic pruning or LTP [36]. Hence, the interpretation of neurotrophin system action presents a challenge because simply knowing the level of BDNF in the system (brain and/or serum) is insufficient to determine whether its effects are beneficial or detrimental. It is also essential to consider the relative ratio of TrkB and p75NTR/sortilin expressions in the specific brain area, as p75NTR receptors may be up-regulated while Trk receptors could be down-regulated in conditions of injury and/or psychiatric disorders [15].

#### 3.2.3. Down-Stream Signaling Mechanisms (Level 3): Intracellular Conflicts and Pathway Divergence

The third level of conflict within the neurotrophin system elements includes non-linear downstream signaling pathways, where the same neurotrophin signals can trigger different or even opposing effects, as they frequently interact with and influence one another [37]. The activation of Trk receptors by mature neurotrophin primarily triggers the PLC-γ1, MAPK/ERK, and PI3K/Akt pathways. Activation of the PLC-γ1 pathway causes the release of intracellular calcium and protein kinase-C (PKC) activation that is crucial for synaptic plasticity [38]. The Akt pathway functions as a key mediator for neuronal survival by inhibiting pro-apoptotic proteins like Bad and Bax, while also promoting anti-apoptotic proteins such as Bcl-2 to sustain mitochondrial function. ERK’s function in neuronal differentiation and neurite growth includes promoting the expression of anti-apoptotic genes via CREB activation [39]. Neurotrophin system elements also help maintain redox balance; specifically, BDNF/TrkB signaling activates neuroprotective pathways via Nrf2 by activating ERK/CREB/BDNF, p38/MAPK/ERK, and IRS/PI3K/AKT and further enhancing mitochondrial function and combating oxidative stress by upregulating SOD and heme oxygenase-1, ultimately inhibiting apoptosis linked to injury and inflammation [40,41]. In general, the neuroprotective effects of BDNF often depend on the Akt and ERK pathways working collaboratively to counteract apoptotic challenges like excitotoxicity and oxidative stress.

Moreover, downstream signaling networks of neurotrophin system elements and Trk receptors include extensive crosstalk, integrating signals from multiple NT/Trk pathways. PLCγ-activated PKC can phosphorylate Raf to create a positive feedback system that connects calcium signaling with MAPK activation. Further, ERK phosphorylates PLCγ, and Akt signaling impacts the ERK pathway through different mechanisms. Also, these pathways have different kinetics: MAPK activation occurs within minutes, PI3K/Akt signaling persists for hours, and calcium responses can be either rapid or sustained. This temporal division enables complex cellular responses by integrating immediate survival signals with differentiation programs [30].

The conflict in the neurotrophin/Trk downstream signaling pathway is determined by the signal’s duration, as prolonged BDNF/TrkB activation enhances dendritic arborization, while transient TrkB stimulation facilitates dendritic growth. In hippocampal slices, the delivery of BDNF at a slow rate enhances LTP, while a rapid application of BDNF increases basal synaptic transmission [42]. The phenotypic shift between cell survival and apoptosis/inflammation is strongly influenced by the temporal pattern of Trk receptor activation. Sustained Trk signaling promotes neuronal survival, differentiation, synaptic plasticity, and anti-inflammatory responses through prolonged activation of the PI3K/Akt and ERK/MAPK pathways. Continuous engagement of these signaling cascades maintains cellular homeostasis and supports neuroprotective functions. In contrast, transient or attenuated Trk activation may be insufficient to sustain these pro-survival mechanisms. Importantly, the phenotypic shift toward apoptosis and inflammation is not triggered by Trk activation itself, but rather by the reduction or loss of Trk-mediated signaling. When neurotrophin availability decreases, or Trk activation becomes short-lived, downstream survival pathways are weakened, resulting in reduced Akt activity, diminished inhibition of pro-apoptotic factors, and increased susceptibility to inflammatory stimuli. Under these conditions, signaling through p75NTR and stress-related pathways, including JNK and NF-κB, may become predominant, driving a phenotypic shift from a neuroprotective and pro-survival state toward apoptosis, neuroinflammation, and cellular dysfunction [42]. Also, BDNF-induced acute and gradual TrkB activation has distinct effects on synaptic activity and plasticity [43]. While transient MAPK/ERK signaling facilitates the proliferation process, the continuous activation of the NGF/TrkA pathway might either cause differentiation [44] or have adverse effects, such as oxidative stress, inflammation, and cell death [45]. Notably, prolonged Trk receptor activation results in their downregulation, indicating a regulatory mechanism for receptor expression beyond sustained activation for continuous growth and renewal [46].

When p75NTR is activated alone or in combination with sortilin and pro-neurotrophin, adaptors such as TRAF6 and NRIF are recruited, which in turn activate the JNK pathway and inhibit the Akt pathway [47]. This switching activates the pro-apoptotic genes (Bim, Bad, and Bax), p53, and cytochrome c release and consequently induces apoptosis, oxidative stress, and neuroinflammation [48]. Consequently, a signaling conflict arises where Akt-driven survival competes with JNK-mediated apoptosis in neurons [49]. The pro-BDNF/p75NTR/sortilin complex triggers JNK/Rho and NF-kB pathways, which diminish neurogenesis, synaptic development, and neuronal survival while enhancing neuronal apoptosis [50].

One of the most complex conflicting areas in neurotrophin signaling is the activation of NF-κB, influenced by both Trk and p75NTR [51]. NF-κB’s effects vary by context; it can either stimulate anti-apoptotic factors like Bcl-2 or promote pro-apoptotic elements such as p53 and BAX, alongside influencing oxidative stress [52] and regulating cytokine production [53]. Understanding NF-κB’s dual role as either neuroprotective or neurotoxic is vital for interpreting neurotrophic therapy effectiveness, highlighting its interaction with the cell’s inflammatory state in determining survival outcomes.

Another challenge in the interpretation of neurotrophin system action lies in the fact that neurotrophin functions as bidirectional modulators of inflammation. BDNF promotes anti-inflammatory effects by transitioning microglia from a pro-inflammatory M1 phenotype to an anti-inflammatory M2 phenotype [54]. p75NTR is associated with a variety of immune functions and shows increased expression in CNS-resident immune cells during insults, particularly during infections such as Toxoplasma gondii, which cause strong p75NTR upregulation in microglia [55]. Research suggests that both pro-NGF-p75NTR and pro-BDNF-p75NTR signaling cascades enhance pro-inflammatory responses, thus contributing to chronic tissue inflammation [56,57] and inflammation-induced pain [58]. Interestingly, while the deletion of p75NTR may enhance synaptic function, it does not affect neuroinflammation or cognitive impairment in the AD mouse model [59]. Conversely, the pro-BDNF/p75NTR/sortilin pathway’s role in severe depression is complex, influencing anti-inflammatory cytokines (IL-1β and IL-10) while simultaneously reducing TNF-α and IL-6, reflecting a conflicting context-dependent role where different neurotrophin system elements can either alleviate or worsen neuroinflammation based on the dominant receptor and neurotrophin subtype [60].

### 3.3. Necessity of a Multifactorial and Multilevel Approach in the Evaluation of Neurotrophin System Actions

The neurotrophin system elements play a critical role in brain development, synaptic plasticity, and the pathophysiology of psychiatric disorders; however, their inherent complexity makes the interpretation of their actions challenging. Functional effects result from the interactions among extracellular neurotrophin system elements, their membrane receptors, and downstream signaling pathways, all of which are influenced by contextual factors. Traditional single-biomarker approaches, such as measuring peripheral and/or brain BDNF levels, are inadequate due to this multilevel complexity. Future research must prioritize integrated profiling techniques that examine multiple neurotrophin system elements and their interrelationships at various levels, since that can be decisive for the outcome, transitioning from a focus on individual biomarkers to identifying biological subtypes with distinct mechanisms and treatment requirements. This systemic analysis is essential for advancing the field of psychiatric neuroscience, facilitating a better understanding of the diversity within psychiatric disorders.

## 4. Neurotrophin System in Depressive Disorders

Depression is a major neuropsychiatric disease that causes a persistent feeling of sadness and loss of interest [61], also characterized by emptiness and irritable mood, accompanied by somatic and cognitive changes that significantly affect the individual’s capacity to function [62]. Although the prevalence of major depressive disorders is high with a continuous tendency to increase, due to misconceptions and the stigma of mental health disorders, more than 50% of people with depression do not seek medical help, despite the fact that their condition represents a significant medical, social, and economic burden [63]. According to the DSM-5, depressive disorders are classified into disruptive mood dysregulation disorder, major depressive disorder (MDD), persistent depressive disorder (dysthymia), premenstrual dysphoric disorder, and depressive disorder due to another medical condition.

Despite the variety of clinical manifestations and forms, some common pathophysiological backgrounds of depressive disorders have been identified. Among others, neurotrophic factors have been confirmed to be linked to the pathogenesis of depression, while the definitive neurotrophic basis remains elusive [64]. In the last decade, substantial basic information on neurotrophin alterations and depressive symptoms under different medical conditions has been accumulated (Table 1). As the preferred starting point, special attention should be given to the data obtained from trials without any applied therapeutic protocols. The recent investigation was conducted on healthy volunteers (with no history of depression and/or other psychiatric disorders, and without specific medication) who underwent reconsolidation-interference protocols, showing no significant improvement for induced depression-like helplessness [65]. However, further analyses suggested that BDNF levels may have mediated outcomes.

The PRONIA study aimed to identify multivariate patterns of peripheral inflammation and gray matter volume in early-stage depressive and psychotic disorders using a transdiagnostic machine learning approach [66]. According to results obtained in this study, the typical blood parameter profile in those patients showed potential interaction between immune-inflammatory (IL-1β and IL-2) and compensatory immune-regulatory (IL-1RA, IL-2, and IL-4) processes linked with potential neurotrophic adaptation (based on increased BDNF), along with characteristic gray matter volume losses in the specific brain regions (predominantly limbic system), which altogether, according to the authors, may represent the typical distinct immune-neurobiological imprint of early-stage depression due to limbic-cortical dysregulation as a core neurobiological feature of depression. The connection between peripheral BDNF levels and depression severity was additionally confirmed in MDD patients with and without a history of suicide attempts, since the plasma BDNF level was significantly lower in suicidal MDD (10% above 684.75 pg/mL) than in non-suicidal MDD patients, and as expected, much lower than in healthy controls (78% above the predefined turn point) [67]. Those observations focus on the role of neurotrophins in modulating neural circuits that change simultaneously during MDD. This is in line with the trial performed on the Taiwanese population, where drug-naive first-episode MDD patients showed significantly lower BDNF levels when compared to healthy subjects [68]. Although they reached similar findings (lower circulating BDNF levels in MDD patients), the research group of Williams also reported higher BDNF levels in female patients with cardiovascular diseases, when compared to males, that persists from depression-naïve patients until very severe cases of MDD [69]. However, a previous investigation conducted by Caldieraro and coworkers did not confirm the relationship between peripheral BDNF levels and MDD severity [70].

**Table 1:** Basic information for neurotrophins alterations and depressive symptoms under different medical conditions (without therapeutic protocols).

Ref.	Type of Study	Number of Patients	NT System Alterations	Proposed Mechanism
[66]	randomized prospective trial	163 (early stage depression)	↑ BDNF	↑ neurotrophic adaptation ↑ immunomodulation
[67]	randomized prospective trial	50 (MDD patients with and without a history of suicide attempt)	↓ BDNF (in suicidal patients)	↑ modulation of neural circuits
[71]	prospective observational study	168 (hyperventilation syndrome)	↓ BDNF	↓ 5-HT ↓ CaMKII
[72]	randomized controlled trial	293 (older adults with chronic pain)	↓ BDNF (in males)	-

Abbreviations, acronyms, and symbols denote (in alphabetical order): 5-HT, 5-hydroxytryptamine; BDNF, brain-derived neurotrophic factor; CaMKII, Ca^2+^/calmodulin-dependent protein kinase II; MDD, major depressive disorder; NT, neurotrophin; Ref., reference; ↑, increased/upregulated; ↓, decreased/downregulated; -, not reported/not applicable.

In contrast, the analysis performed in patients with hyperventilation syndrome who expressed depression symptoms showed decreased BDNF (app. 15%) along with 5-HT levels when compared to non-depressed patients [71]. The authors proposed that calcium/calmodulin-dependent protein kinase II (CaMKII), which correlates with BDNF and 5-HT, could be a risk factor for depression in patients with hyperventilation syndrome. Another trial was conducted to clarify the interaction between depression severity and BDNF levels in the context of chronic pain in older adults [72]. Depressive patients exposed to chronic pain showed significantly lower values of BDNF in comparison to healthy subjects (app. 20% in intense pain). Interestingly, this relationship was observed only in male patients, but the authors claimed that more elements of the neurotrophin system should be involved in the analysis to reach reliable conclusions. In line with that, the trial performed in the elderly population that experienced post-stroke depression showed that increased peripheral BDNF levels negatively correlated with the risk of post-stroke depression [73], favoring the neuroprotective role of BDNF. The other group of researchers [74] also reached the same findings in patients with post-stroke depression after a minor stroke at three months, where peripheral BDNF levels negatively correlated with depression severity symptoms (only in females). Interestingly, progressive decline in peripheral BDNF levels was observed among patients with alcohol dependence and depression, with the lowest values found in patients with alcohol dependence and comorbid depression [75]. In an earlier study, Tschorn and colleagues [76] analyzed the relationship between BDNF and depression in a cohort of patients with coronary heart disease. The initial report revealed that significantly lower levels of BDNF were observed in the patients with more severe depressive symptoms. Numerous therapeutic protocols (pharmacotherapy, programmed physical activity, dietary protocols, transcranial stimulation, and cognitive behavioral therapy) were individually included in attempts to address depression; however, the search for reliable biomarkers that could follow the outcome has only recently brought attention to the elements of the NT system.

As shown in Table 2, the most frequent therapeutic approach in the treatment of depressive disorders is pharmacological. Probably due to its rapid action, it is not surprising that most pharmacological interventions that include NT alterations in depression treatment employ ketamine administration. The prevalence of depressive episode appearance in the context of surgical interventions attracted the attention of numerous research groups, looking for potential preventive and/or curative pharmacologic protocols. The combined perioperative (intra- and immediate postoperative) administration of esketamine (0.25 or 0.5 mg/kg), along with sufentanil, in patients undergoing laparoscopic total hysterectomy showed beneficial effects [77]. Namely, esketamine relieved postoperative depression (especially with higher doses), which occurred concomitantly with increased BDNF and 5-HT levels on days 1, 2, and 5 after the surgery. Those effects lasted up to one week after the operation, reaching the maximal effect (10% in the lower dose and 15% in the higher dose) after 24 h. Interestingly, a lower dose (0.3 mg/kg) of perioperatively administered esketamine in patients with cardiac valve surgery [78] resulted in the antidepressant effect accompanied by increased BDNF that remained significant even after seven days, with maximal effect (increase of 20–25%) in the early phase (day 1–3). The authors attributed those effects to neurotrophin-induced modulation of neuroinflammation. An intriguing issue, considering the schedule of esketamine administration along with surgery was the focus of a trial performed by Dai and colleagues in laparoscopic bariatric surgery patients [79]. To define the optimal timing for perioperative esketamine administration, the authors explored the effects of the same esketamine (subanesthetic) dose (0.2 mg/kg) applied prior (2 h) and after the induction of anesthesia on postoperative depression and peripheral BDNF levels. Testing one day after surgery revealed that the use of an applied esketamine dose after anesthesia failed to improve postoperative depression symptoms, while the use of esketamine before anesthesia could improve postoperative depression symptoms. At the same time, plasma BDNF levels were significantly increased only after preoperative esketamine administration (25–30% when compared to placebo, as well as postoperative administration). As expected, BDNF levels negatively correlated with depression symptoms. The benefits of adjunctive esketamine administration (0.5 mg/kg, perioperatively) on surgery-related depression were also confirmed in elderly patients undergoing hip fracture surgery [80], since it improved depressive symptoms and increased peripheral levels of BDNF (for 10%) and 5-HT. The antidepressant action of intraoperative ketamine administration was also observed in the study conducted by Jiang and collaborators involving patients undergoing elective orthopedic surgery [81]. Namely, ketamine, given at induction of anesthesia (0.5 mg/kg), followed by a continuous infusion of 0.025 mL/kg/h for 30 min, resulted in improved scores for depressed mood and increased serum BDNF levels (for only 10%, but interestingly increased values instead of decreased in placebo group). Postpartum depression is a significant health burden and has been linked to specific fluctuations in the levels of neurotransmitters and neurotrophins. Again, the fast-acting drug esketamine was employed in the trial performed by Jiang et al. involving this population [82]. According to the authors, esketamine (0.25 mg/kg) showed a strong antidepressant effect (determined on day 3), while also reversing the monoamine transmitter levels (5-HT and dopamine) and increasing BDNF (by 50%) levels. Those alterations have been attributed to the positive impact of esketamine in the postpartum period. A different group of participants, treatment-resistant MDD patients, did not significantly respond positively by means of BDNF levels following a single dose of ketamine (0.5 mg/kg) or esketamine (0.25 mg/kg) intervention, despite clinical improvement [83].

Numerous investigations evaluating the efficacy of various antidepressants also included the determination of certain neurotrophic factors. Thus, Mishra and colleagues explored the comparative efficacy of antidepressants with amantadine (200 mg/day), pramipexole (375 mg/day), and quetiapine (100 mg/day) as augmentation to ongoing sertraline treatment for eight weeks in patients with treatment-resistant unipolar depression [84]. Interestingly, the clinical outcome achieved with pramipexole was significantly above the effects of other applied drugs. At the same time, improvements in depressive symptoms were corroborated with increased BDNF (20–25% after 4 weeks, and more than twice after 8 weeks) and NGF (significant increase only after 8 weeks) levels in all patients, but with no difference between the administered antidepressants, suggesting a role of neurotrophins in promoting neuronal resilience and recovery. The beneficial effects by means of both clinical outcome (decreased depressive signs) and increased BDNF levels were presented by Gupta and investigators following the trial with mirtazapine (30 mg/day) for 12 weeks in MDD patients [85], with a proposed anti-inflammatory response (decreased TNF-α) as the potential mechanism. The evaluation of the single antidepressant (agomelatine) efficiency in MDD patients was also conducted by Martinotti and fellows [86] and it resulted in the confirmed antidepressant action of agomelatine (25–50 mg/day), accompanied by a simultaneous increase in peripheral BDNF levels (initial increase of 40%). Both beneficial effects were observed after two weeks of agomelatine administration and persisted for up to eight weeks (although with no additional significant increase in BDNF).

Another comparative study to examine the efficiency of individual antidepressants was conducted by Gupta et al. in MDD patients [87]. Both fluoxetine (20 mg/d) and agomelatine (50 mg/d) in a 12-week trial resulted in improved depression symptoms and increased BDNF levels (app. 15%). As applied protocols also produced a significant decrease in TNF-α, the authors proposed that the anti-inflammatory effect of BDNF could be a potential beneficial mechanism of MDD treatment (more with agomelatine compared to fluoxetine). Similarly, the whole blood BDNF (wb-BDNF) levels also remained unchanged by escitalopram (10–20 mg/day for 12 weeks) in patients with depression, according to a study by Navarro et al. [88]. Although the results of this trial did not cover clinical outcome evaluation, the authors showed that whole blood BDNF levels were lower in unmedicated MDD patients than in healthy controls (approximately 14%). Based on the presented results, Navarro and colleagues suggested that wb-BDNF could be used as a diagnostic but not as a prognostic biomarker in MDD. The pediatric population of depression patients was employed in the clinical trial of Wu and coworkers [89], which revealed beneficial effects of sertraline on depressive symptoms. Clinical outcome was accompanied by increased serum BDNF and 5-HT levels, as well as decreased inflammatory cytokine expression. Interestingly, the observed benefits of sertraline appeared as an early response to therapy (in 2 weeks) but leveled-up to baseline values after 4 weeks (except for the pro-inflammatory markers). The presented results lead to the conclusion that the antidepressant effect initially involved improvement in neurotrophin levels, which was followed by continuous immunomodulation.

To examine the sex-controlled differences in sertraline (100 mg/day) and citalopram (40 mg/day) efficacies in MDD patients, Shamabadi and collaborators analyzed their effects not only on depressive symptoms but also on peripheral BDNF levels, following an 8-week protocol [90]. There were no significant differences between sertraline and citalopram effects on clinical outcome (significant improvement in both groups), but the authors observed a significantly greater increase in male BDNF levels following sertraline treatment. Additional analysis showed significant associations between changes in IL-6 levels and BDNF levels in the sertraline group, as well as with depression severity scores in citalopram recipients.

Ketamine, as a rapid-acting treatment for treatment-resistant depression, was also used in the study by Rengasamy and collaborators to examine the potential connection between clinical features and neurotrophin and inflammatory markers [91]. A single infusion of ketamine (0.5 mg/kg) resulted in strong effects on depressive symptoms, but this was linked to changes in peripheral inflammatory markers, as well as BDNF. Still, the authors proposed that the lack of significant correlation between clinical outcome and estimated markers may be due to study limitations (i.e., sample size). Moreover, it should be highlighted that the effect of low-dose ketamine infusion in treatment resistant depression (TRD) patients strongly depends on BDNF polymorphism to the extent that the dosage of ketamine should be adjusted according to the degree of treatment refractoriness and BDNF rs6265 polymorphism to achieve the optimal antidepressant effect for patients with TRD [92]. The importance of BDNF polymorphism was also confirmed in patients with acute coronary syndrome who express depressive symptomatology [93]. The authors observed that patients with BDNF met alleles were more vulnerable to depressive disorders from the baseline (at the onset) and to depressive disorder persistence. Following the success of ketamine in MDD patients, additional clinical research on NMDA antagonists has been performed to achieve safe and long-term therapy. Hence, Maji et al. evaluated the effects of adjunct dextromethorphan (30 mg/day for 8 weeks) to SSRIs in mild to moderate MDD patients [94]. The improvement in symptoms, response, and remission rate was achieved with dextromethorphan, but with no significant difference in serum BDNF levels.

Another add-on to standard therapy that acts by modulating the NMDA receptor is sarcosine (an endogenous amino acid), which was applied with SSRIs in the management of MDD patients [95]. When compared to the results obtained from standard therapy, sarcosine supplementation (500 mg daily for eight weeks) produced a more pronounced antidepressant effect associated with upregulation in BDNF levels (for more than 10%), which led the authors to propose sarcosine as an efficient and safe add-on therapy. Very convincing results for the benefits of an add-on to standard SSRI therapy for MDD in adult patients were presented by Merza Mohammad et al. [96]. Pentoxifylline (400 mg twice daily), a phosphodiesterase inhibitor, was used as an add-on with citalopram (20 mg/day) to reduce pro-inflammatory activities for 12 weeks. Indeed, this combination resulted in a significant decline in MDD severity, which appeared along with a significant increase in BDNF and serotonin levels. The specific feature of pentoxifylline was manifested by the reduction in serum concentrations of pro-inflammatory factors, thus promoting this therapeutic strategy for beneficial modulation of neuroinflammatory processes.

The benefits of adjunctive therapy were also observed in the study presented by Esalatmanesh et al. [97], as shown in Table 2. The authors included celecoxib (200 mg twice a day), an anti-inflammatory agent, along with cognitive behavioral therapy (CBT) in patients with mild to moderate postpartum depression for six weeks. According to their results, the effect of celecoxib as an add-on was manifested by a significant decrease in depression severity, as well as by increased BDNF levels (app. 10%) and a reduction in inflammatory cytokines. For that reason, the authors proposed neuroprotective action, by means of decreased neuroinflammation, as the major beneficial effect. The study aimed to compare the efficacy and safety of low-dose amisulpride (100–300 mg/day) with that of the olanzapine-fluoxetine combination (5/10 mg + 20 mg) in post-schizophrenic depression (PSD) patients [98]. After the eight-week protocols in this head-to-head clinical trial, the authors determined the improvement in clinical signs, along with a significant increase in the serum BDNF levels, with up to a 10% increase (no significant difference between therapeutic protocols), with a significant negative correlation between the changes in the scores and BDNF levels in each group. Although both protocols showed beneficial effects, there were significant differences between the two therapeutic PSD groups.

Protocols aimed at reducing depression severity based on the programmed physical activity are also widely employed (Table 2). The investigation conducted by de Souza and colleagues on HIV patients, who often experience depressive symptoms, showed significant benefits from resistance training [99]. Eight weeks of programmed physical activity resulted in a significant decline in depressive symptoms; however, peripheral BDNF levels remained unaltered. Another programmed physical activity (high-intensity interval training, Nordic walking, and moderate-to-vigorous intensity continuous training) was explored for its effects on depression severity in patients with coronary artery disease enrolled in cardiac rehabilitation [100]. Like the findings in HIV patients, this 12-week program improved depressive symptoms, but again, this improvement could not be attributed to an increase in BDNF levels.

In contrast, aerobic-resistance training (for 12 weeks) showed beneficial effects in females with type 2 diabetes mellitus, as manifested by both decreased depression severity and increased circulating BDNF levels (app. 50%). Interestingly, the increased BDNF levels persisted even after an eight-week detraining protocol [101]. A complex machine-assisted programmed physical activity (whole-body vibration training program), when applied to women with fibromyalgia syndrome [102], also improved depressive symptoms with concomitant elevation of BDNF (more than 30%), and the authors attributed this improvement to aspects related to biological rhythm and increased peripheral BDNF levels. Physical activity (6-month resistance training, 3 times per week) also showed beneficial effects, as a non-pharmacological tool, in hemodialysis patients with depressive symptoms, by means of decreased depressive symptom intensity, along with increased BDNF levels (more than 50%) [103]. The extensive analysis of oxidative stress markers performed in this trial revealed a potential role of BDNF in improving oxidative balance.

Two different forms of physical activity, dance and martial arts, were employed in a trial performed by Hola and coworkers [104]. Older adults were tested for geriatric depression symptoms, along with BDNF and irisin levels, due to their confirmed neuroprotective actions. Both 12-week exercise protocols resulted in clinical improvement, as well as in upregulated BDNF levels (maximal effect at 10%), with no significant changes in irisin. The authors proposed a neuroprotective role (increased neuroplasticity) of BDNF as the underlying beneficial mechanism, which has been more pronounced in aerobic than in resistance training. Furthermore, the other research team included a home exercise program (45 min twice a week) to manage upper limb therapy during post-stroke rehabilitation, which was additionally accompanied by a music-enriched environment protocol (performed by an occupational and music therapist) for six weeks [105]. This interesting combination resulted in a reduction in post-stroke depression and increased peripheral BDNF (app. 50%). The authors proposed that observed BDNF augmentation may be responsible for optimization of the neural environment and favored neuronal recovery. Unlike previous reports, the results presented by Cartmel and investigators for the impact of exercise (a six-month home-based moderate-intensity aerobic program facilitated by weekly phone calls from trainers, 150 min/week) in ovarian cancer survivors [106] showed significant benefits by means of clinical outcome (decreased depression severity) but did not confirm the alterations in peripheral BDNF levels.

Probably the most popular and commonly accepted, for its safety and availability, variety of dietary and other food intake interventions (Table 2) was also the basic therapeutic approach in the treatment of depressive patients. Total restriction of food intake (10 h during the night followed by 10 h of breakfast) was performed in female adults (obese and non-obese) by Tibaes et al. to evaluate the impact on depressive mood and neurotrophin levels [107]. Deterioration of depressive symptoms after the fasting protocol was observed in only the obese group, while neurotrophins alterations were more complex–NGF increased in the non-obese group, while BDNF and glial cell line-derived neurotrophic factor (GDNF) remained unchanged. The authors proposed that fasting interventions should be tailored to individual needs according to anthropometric parameter values at the baseline.

Increased incidence of depression in type 2 diabetes mellitus patients, usually accompanied by vitamin D deficiency, initiated the research group of Putranto to evaluate the potential benefits of cholecalciferol supplementation on depressive symptoms [108]. Indeed, the authors showed that cholecalciferol add-on (4000 IU daily, for 12 weeks) resulted in significant antidepressant action, but their findings did not support the hypothesis that potentiated the neurotrophin theory of depression (according to NT-3 levels). Instead, the authors proposed enhancing c-peptide levels as a key mechanism. Another clinical trial was also based on vitamin D supplementation (50,000 IU/week), with or without simultaneous magnesium supplementation (250 mg/day), in obese women with mild to moderate depressive symptoms for 8 weeks [109]. Both supplements produced beneficial influences on depression severity, as well as serum levels of BDNF (increase of up to 10%) and inflammatory markers. The authors reported the greatest improvement in the group with combined administration of supplements and suggested diminished neuroinflammation as a potential beneficial mechanism. However, these results contrast with the previous findings that showed no impact of magnesium supplementation, even at twice the dose (500 mg/d) in the same timeframe, although with the same beneficial clinical outcome in MDD patients [110].

On the other hand, individual supplementation with vitamin D and magnesium, as well as their co-supplementation (Table 2), and supplementation with vitamin D and zinc in the same group of participants (patients with obesity and mild to moderate depressive symptoms) provided evidence of benefits [111]. This is in line with the mixed results for the impact of calorie restriction protocols on neurotrophic status. Although it is well known that obesity is accompanied by lower BDNF levels, therefore by impairment of BDNF/TrkB signaling that results in reduced energy expenditure and increased fat accumulation [112], the impact of various continuous calorie restrictions is still controversial [113]. The applied protocol yielded significant improvement in depressive symptoms (pronounced in patients with more severe depression), but none of the dietary protocols induced a significant increase in BDNF levels. The incidence of depressive disorders is also notably higher in multiple sclerosis patients. The population was employed in a trial performed by Hajiluian et al. [114] to evaluate the effects of ellagic acid supplementation on clinical outcomes and various peripheral markers. This natural polyphenol compound was applied for 12 weeks (180 mg/d) and resulted in a significant improvement in depression symptoms. BDNF and serotonin levels were enhanced in the experimental group of patients, while cortisol levels were decreased. The authors attributed the multiple beneficial effects of this dietary intervention to improved neurotrophin and neurotransmitter balance as well as HPA axis activity. Another successful attempt to achieve clinical improvement in MS patients with significant depression severity by using dietary interventions was observed in a trial by Rahimlou et al. [115]. Two multi-strain probiotic capsules daily for six months resulted in a significant reduction in depression symptoms, as well as an increase in BDNF, concomitantly with a significant reduction in IL-6 levels. The beneficial effects were, according to the authors, succeeded by the regulation of various signaling pathways, including inflammatory modulation.

Not all dietary interventions presented in the last decade confirmed the positive impact on the peripheral BDNF levels in MDD patients. Thus, in a trial conducted by Foshati et al. [116], the authors evaluated the effects of extra-virgin olive oil supplementation (25 mL/d for 52 days) and compared it to adequate intake of sunflower oil in randomized groups of patients. Although they reported improvement of depression symptoms (in patients with severe depression but not in those with mild to moderate depression), there was no significant change in peripheral BDNF levels.

Based on the fact that exogenous melatonin demonstrated a positive influence on depressive symptoms and sleep quality in breast cancer patients, Palmer and investigators [117] conducted a study that included breast cancer patients on chemotherapy who were supplemented with melatonin (20 mg/d for 10 days). Along with improved sleep quality, the applied protocol also decreased depression severity. The beneficial clinical outcomes occurred simultaneously with increased serum BDNF (by two-fold) and TrkB (app. 15%) expression, which led the authors to the conclusion that positive melatonin impact could be accompanied by increased neuroplasticity.

Although with a much shorter history in the treatment of depressive disorders, transcranial stimulation and cognitive behavioral therapy have attracted more attention in the last decade (Table 2). As one of the most sophisticated methodologies with potential for home-based application, transcranial stimulation (as a single therapy) was performed in the study by Pan et al. [118]. The authors presented beneficial effects of neuronavigation-guided rTMS (repetitive transcranial stimulation, for 7 consecutive days over one week), by means of significant clinical improvement, when compared to treatment-naïve depressive patients with suicidal ideation, but with no influence on serum BDNF and TrkB. The author postulated that high-dose rTMS rapidly regulates the serum BDNF-TrkB pathway and therefore upgrades the beneficial effects. However, it is important to notice that response to rTMS (and theta burst stimulation) in TRD patients strongly depends on BDNF polymorphism, especially Val66Met polymorphism [119], which contrasts with the results obtained with other methods of transcranial stimulation (direct current stimulation, tDCS), where BDNF polymorphism did not affect the clinical outcome, as previously reported for both unipolar and bipolar depression [120,121].

**Table 2:** Individual therapeutic approaches in depressive symptoms treatment according to neurotrophin system alterations.

Ref.	Type of Study	Number of Patients	Therapeutic Protocol	Clinical Outcome	NT System Alterations	Proposed Mechanism
**Pharmacotherapy**						
[77]	randomized controlled trial	83 (laparoscopic total hysterectomy)	esketamine (0.25 or 0.5 mg/kg)	Positive (↓ depressive symptoms)	↑ BDNF	modulation of BDNF and 5-HT pathways
[78]	randomized controlled trial	71 (cardiac valve surgery)	standard anesthesia + esketamine (0.3 mg/kg)	Positive (↓ depressive symptoms)	↑ BDNF	↓ neuroinflammation
[79]	randomized controlled trial	80 (laparoscopic bariatric surgery)	esketamine (0.2 mg/kg) prior or along with anesthesia	Positive (↓ depressive symptoms)–only prior	↑ BDNF (only prior)	↑ downstream processing
[80]	randomized controlled trial	45 (elderly patients on hip fracture surgery)	standard anesthesia + esketamine (0.5 mg/kg)	Positive (↓ depressive symptoms)	↑ BDNF	↑ neurotransmitter release ↑ neurotrophin production
[81]	randomized controlled trial	60 (elective orthopedic surgery)	standard anesthesia + ketamine (0.5 mg/kg) at induction + 0.25 mg/kg/h for 30 min	Positive (↓ depressive symptoms)	↑ BDNF	-
[82]	randomized controlled trial	152 (postpartum depression)	standard therapy + esketamine (0.25 mg/kg)	Positive (↓ depressive symptoms)	↑ BDNF	↑ neurotransmitter release ↑ neurotrophon production
[84]	randomized controlled trial	150 (treatment-resistant unipolar depression)	amantadine (200 mg/d), pramipexole (375 mg/d), quetiapine (100 mg/d) for 8 weeks	Positive (↓ depressive symptoms)	↑ BDNF ↑ NGF	↑ neuronal resilience and recovery
[85]	randomized controlled trial	30 (MDD)	mirtazapine (30 mg/d) for 12 weeks	Positive (↓ depressive symptoms)	↑ BDNF	↓ neuroinflammation
[86]	randomized controlled trial	27 (MDD)	agomelatine (25–50 mg/d) for 8 weeks	Positive (↓ depressive symptoms)	↑ BDNF	-
[87]	randomized controlled trial	60 (MDD)	fluoxetine (20 mg/d) or agomelatine (50 mg/d) for 12 weeks	Positive (↓ depressive symptoms)	↑ BDNF	↓ neuroinflammation
[88]	randomized controlled trial	75 (MDD)	escitalopram (10–20 mg/d) for 12 weeks	-	BDNF n.c.	-
[89]	randomized controlled trial	82 (pediatric depressive patients)	sertraline for 4 weeks	Positive (↓ depressive symptoms)	BDNF n.c. (↑ BDNF–early response)	↑ 5-HT ↑ immunomodulation
[90]	randomized controlled trial	80 MDD (40 males + 40 females)	sertraline (100 mg/d), citalopram (40 mg/d) for 8 weeks	Positive (↓ depressive symptoms)	↑ BDNF (males)	-
[91]	randomized controlled trial	89 (treatment-resistant unipolar depression)	ketamine (0.5 mg/kg, single infusion)	Positive (↓ depressive symptoms)	BDNF n.c.	-
[93]	randomized controlled trial	30 (MDD)	SSRIs + dextromethorphan (30 mg/d) for 8 weeks	Positive (↓ depressive symptoms)	BDNF n.c.	-
[95]	randomized controlled trial	60 (MDD)	SSRIs + sarcosine (500 mg/d), for 8 weeks	Positive (↓ depressive symptoms)	↑ BDNF	↑ neuroprotection
[96]	randomized controlled trial	50 (MDD)	SSRIs + pentoxifylline (800 mg/d) for 12 weeks	Positive (↓ depressive symptoms)	↑ BDNF	↓ neuroinflammation
[97]	randomized controlled trial	50 (postpartum depression)	celecoxib (400 mg/d) + CBT for 6 weeks	Positive (↓ depressive symptoms)	↑ BDNF	↓ neuroinflammation
[98]	randomized controlled trial	60 (PSD)	amisulpride (100–300 mg/d) or olanzapine-fluoxetine (5/10 mg + 20 mg) for 8 weeks	Positive (↓ depressive symptoms)	↑ BDNF	-
**Programmed physical activity**						
[99]	randomized controlled trial	11 (HIV)	resistance training for 8 weeks	Positive (↓ depressive symptoms)	BDNF n.c.	-
[100]	randomized controlled trial	135 (coronary artery disease)	programmed exercise for 12 weeks	Positive (↓ depressive symptoms)	BDNF n.c.	-
[101]	randomized controlled trial	34 (females with type 2 diabetes mellitus)	aerobic-resistance training (for 12 weeks)	Positive (↓ depressive symptoms)	↑ BDNF	-
[102]	randomized controlled trial	32 (females with fibromyalgia syndrome)	whole-body vibration training for 6 weeks	Positive (↓ depressive symptoms)	↑ BDNF	-
[103]	randomized controlled trial	81 (hemodialysis patients)	resistance training (3/w) for 6 months	Positive (↓ depressive symptoms)	↑ BDNF	↑ oxidative balance
[104]	randomized controlled trial	50 (older healthy adults)	dance or martial arts for 12 weeks	Positive (↓ depressive symptoms)	↑ BDNF	↑ neuroplasticity
[105]	pilot randomized controlled trial	30 (post-stroke depression)	home exercise program + music-enriched environment protocol for 6 weeks	Positive (↓ depressive symptoms)	↑ BDNF	↑ neuroregeneration
[106]	randomized controlled trial	74 (ovarian cancer survivors)	home-based exercise protocol for 6 months	Positive (↓ depressive symptoms)	BDNF n.c.	-
**Dietary protocols**						
[107]	randomized controlled trial	53 (obese and non-obese female adults)	fasting (10 + 10 h)	Negative (↑ depressive symptoms in obese)	BDNF n.c. ↑ NGF (in non-obese) GDNF n.c.	-
[108]	randomized controlled trial	38 (diabetes mellitus type 2)	cholecalciferol (4000 IU/d) for 12 weeks	Positive (↓ depressive symptoms)	NT-3 n.c.	-
[109]	randomized controlled trial	77 (obese female adults with mild to moderate depression)	vitamin D (50,000 IU/w) and/or magnesium (250 mg/d) for 8 weeks	Positive (↓ depressive symptoms)	↑ BDNF	↓ neuroinflammation
[110]	randomized controlled trial	46 (MDD)	magnesium (500 mg/d) for 8 weeks	Positive (↓ depressive symptoms)	BDNF n.c.	-
[111]	randomized controlled trial	140 (MDD)	vitamin D (2000 IU/d) and/or zinc (30 mg/d) for 12 weeks	Positive (↓ depressive symptoms)	BDNF n.c.	-
[114]	randomized controlled trial	29 (MS)	ellagic acid (180 mg/d) for 12 weeks	Positive (↓ depressive symptoms)	↑ BDNF	↑ neurotrophin balance ↑ neurotransmitter balance ↑ HPA axis activity
[115]	randomized controlled trial	32 (MS)	multi-strain probiotic (2 capsules/d) for 6 months	Positive (↓ depressive symptoms)	↑ BDNF	↓ neuroinflammation
[116]	randomized controlled trial	73 (MDD)	extra-virgin olive oil (25 mL/d) for 52 days	Positive (↓ depressive symptoms)	BDNF n.c.	-
[117]	randomized controlled trial	36 (breast cancer patients on chemotherapy)	melatonin (20 mg/d) for 10 days	Positive (↓ depressive symptoms)	↑ BDNF ↑ TrkB	↑ neuroplasticity
**Transcranial stimulation and cognitive behavioral therapy**						
[118]	randomized controlled trial	31 (treatment-naive depressive patients with suicidal ideation)	neuronavigation-guided rTMS (7 days)	Positive (↓ depressive symptoms)	BDNF n.c. TrkB n.c.	-
[122]	randomized controlled trial	26 (MDD undergraduate population)	MCBT (2.5 h/w) for 8 weeks	Positive (↓ depressive symptoms)	↑ BDNF	↓ neuroinflammation
[123]	randomized controlled trial	97 (infertile women)	MCBT (2 h/w) for 10 weeks	Positive (↓ depressive symptoms)	BDNF n.c.	-

Abbreviations, acronyms, and symbols denote (in alphabetical order): 5-HT, 5-hydroxytryptamine; BDNF, brain-derived neurotrophic factor; CBT, cognitive behavioral therapy; GDNF, glial cell line-derived neurotrophic factor; HIV, human immunodeficiency virus; HPA, hypothalamic–pituitary–adrenal; MCBT, mindfulness-based cognitive behavioral therapy; MDD, major depressive disorder; MS, multiple sclerosis; n.c., no significant change; NGF, nerve growth factor; NT, neurotrophin; NT-3, neurotrophin-3; PSD, post-stroke depression; Ref., reference (number); rTMS, repetitive transcranial magnetic stimulation; SSRIs, selective serotonin reuptake inhibitors; TrkB, tropomyosin receptor kinase B; ↑, increased/upregulated; ↓, decreased/downregulated; -, not reported/not applicable.

Finally, cognitive behavioral therapy as the principal therapeutic approach for depressive disorder treatment was the subject of several trials that included the evaluation of neurotrophin system elements for potential use as biomarkers (Table 2). Mindfulness-based cognitive therapy (MCBT) alone was used for the treatment of MDD in undergraduate students [122]. The 8-week protocol (2.5 h sessions and 1 day of practice- a day of silence) was sufficient to reduce MDD symptoms, along with improvement in anxiety levels and sleep. The clinical outcomes occurred concomitantly with increased peripheral BDNF levels (more than 10%) and improved cytokine profiles. The beneficial effects appeared to be proportional to the duration of the protocol. However, the mindfulness-based program (2 h weekly for 10 weeks) aimed at stress reduction in infertile women did not significantly alter BDNF levels despite the obvious improvement in depressive symptoms, according to the results of the study by Nery et al. [123].

It is not surprising that to maximize the benefits achieved with various individual therapeutic protocols, numerous authors have made attempts to employ the combined therapies in depressive disorder treatment (Table 3). Almost all combined therapeutic protocols followed the algorithm based on standard pharmacological therapy with add-ons in the form of other monotherapies.

Repetitive transcranial magnetic stimulation showed numerous benefits in MDD patients currently undergoing antidepressant therapy, according to results obtained by Ozkan and colleagues [124]. The rTMS protocol (20 sessions in a month) was sufficient to reduce the MDD symptoms while significantly increasing peripheral BDNF and GDNF levels (25% and 10%, respectively). Simultaneously, the applied treatment reduced oxidative stress and restored thiol-disulfide balance in MDD patients. The beneficial impact of rTMS was attributed to the modulation of neurotrophic factors and neuroactive steroids. Similar results were presented following the trial of Wang and collaborators [125] performed in middle-aged and elderly patients with MDD. Compared with standard antidepressant therapy (escitalopram, 5–20 mg/d), employment of rTMS (20 min/d, 5 days/w) additionally improved clinical outcome (depression and cognitive function) and increased peripheral BDNF levels after eight weeks (for 20%). The authors proposed that neuroprotection was based on the increased nerve cell growth due to BDNF action. The effectiveness of rTMS (5 sessions/w for 8 weeks) was additionally confirmed during simultaneous administration with agomelatine (25 mg to 75 mg/d) in the study performed by Pu et al., which employed adult patients with mild to moderate depressive disorder [126]. The combination applied in this trial resulted in both clinical improvement and increased peripheral BDNF levels (for 30%, after 8 weeks), along with boosting neurotransmitter content. On the other hand, tDCS (20 or 30 min sessions in two weeks), when applied along with sertraline (50 mg/day) in middle to moderate depressive patients, resulted in improved depressive symptoms with unchanged BDNF levels [127]. When applied along with paroxetine, acupuncture, as another form of peripheral stimulation, had positive effects in the treatment of mild to moderate depression [128]. As this beneficial impact was accompanied by increased serum BDNF levels, in-depth analysis based on DNA methylation revealed that the turning point could be addressed to decrease CpG31 methylation of BDNF. In addition, the lower mean BDNF DNA methylation was also associated with impaired antidepressant response to escitalopram [129].

The impact of Baduanjin exercise (Chinese traditional exercise for 8 weeks) on patients with poststroke depression was evaluated as additional therapy to standard pharmacotherapy (escitalopram oxalate 25–50 mg/d) and rational emotive behavior therapy (REBT, 30 min, 5 times/week) by Liu et al. [130] (Table 3). This innovative therapeutic strategy resulted in a significant improvement in illness severity (along with sleep regulation) when compared to the initial combination. Also, it was sufficient to increase BDNF (app. 30%) and 5-HT levels, while reducing the level of serum proinflammatory factor IL-6. The authors proposed the neurotrophin-induced decrease in neuroinflammation as a potential key mechanism for the beneficial effects of this specific therapeutic combination. Mindfulness-based cognitive therapy, as a psychosocial program used to prevent relapse/recurrence in MDD (120 min, once a week), was applied in combination with standard pharmacotherapy (escitalopram, 5–20 mg/d) by Guo and coworkers [131]. Eight weeks of combined protocol resulted in improvement of clinical outcome while simultaneously remarkably increasing BDNF and NGF levels (25% and 10%, respectively), which authors attributed to the beneficial total effect. Again, the impact of polymorphism on the resilience scores in response to cognitive therapy in MDD patients, evaluated by Peters et al. [132], revealed that initial resilience scores were higher in patients with the Met allele than in the Val/Val genotype. Furthermore, cognitive therapy was more efficient, by means of increased resilience scores and decreased depressive symptoms, observed with the Met allele. This observation also applies to the dose-dependent response to ketamine in TRD patients [133] as well as in elderly male depressive patients undergoing physical activity programs [134].

Another interesting combination of therapeutic approaches was performed by means of dietary intervention (Zishen pingchan granules–ZPG) and standard pharmacotherapy (pramipexole) in a trial performed by Ning and colleagues [135] in depressed patients with Parkinson’s disease (PD) (Table 3). ZPG (13.5 g/d), a traditional Chinese herbal formula, when combined with pramipexole (0.75 mg/d) for 12 weeks, significantly decreased depression severity and prevented a reduction (of 30%) in BDNF levels and an increase in IL-6 levels. The described neuroprotective and antidepressant effects were attributed to decreased neuroinflammation. In contrast, a therapeutic combination that consisted of standard antidepressants and inulin supplementation was applied to women with obesity and depression on a calorie-restricted diet [136]. Based on data showing that gut microbiota dysbiosis is observed in both MDD and obesity, the authors focused their innovative therapeutic approach on estimating the effects of prebiotics to address the shared pathophysiological background. The results of this short-term trial revealed that prebiotic supplementation had no significant beneficial effects, when compared to standard therapy, on depressive symptoms, BDNF levels, and inflammatory biomarkers. The beneficial effects of combined therapeutic protocols, based on dietary interventions along with standard therapy, were also confirmed in the depressive children and adolescents [137]. Dietary add-on was performed in the form of 12-week omega-3 FA (fish oil, 20 mL/d) or omega-6 FA (sunflower oil, 20 mL/d) supplementation in patients with depressive and depressive/anxiety symptoms. While omega-6 supplementation had no significant impact on the evaluated parameters, omega-3 FA supplementation resulted in decreased depression symptomatology, which appeared along with increased peripheral BNDF levels (app. 30%). Interestingly, those effects were observed only in patients without anxiety symptoms. The other supplementation protocol (along with standard therapy) based on the attempt to improve oxidative balance was employed by Hasebe and colleagues using N-acetylcysteine (NAC, 2000 mg/d for 12 weeks) [138]. Unlike the FA diet, NAC supplementation did not result in significant peripheral BDNF alterations, although it improved depressive signs.

Szuhany and coworkers, along with standard therapy, applied the specific exercise protocol (adjunctive exercise or stretching control intervention, after a brief behavioral activation, for 12 weeks) in MDD patients but did not confirm a relationship between BDNF levels and depression severity [139]; although a significant and gradual increase in BDNF was achieved with physical activity (15–20%), there was no improvement in depression symptoms. The very similar observations for programmed physical activity followed the trial of Kerling and collaborators (increased BDNF level when compared to standard therapy in sedentary patients) [140].

**Table 3:** Combined therapeutic approaches in depressive symptoms treatment according to neurotrophin system alterations.

Ref.	Type of Study	Number of Patients	Therapeutic Protocol	Clinical Outcome	NT System Alterations	Proposed Mechanism
[139]	randomized controlled trial	29 (MDD)	standard therapy + adjunctive exercise or stretching control for 12 weeks	n.c.	↑ BDNF	-
[124]	randomized controlled trial	25 (MDD)	standard therapy + rTMS (20 sessions/month)	Positive (↓ depressive symptoms)	↑ BDNF ↑ GDNF	↓ oxidative stress
[125]	randomized controlled trial	60 (MDD, middle-aged and elderly)	escitalopram, (5–20 mg/d) + rTMS (20 min/d, 5 days/w)	Positive (↓ depressive symptoms)	↑ BDNF	↑ nerve cell growth
[126]	randomized controlled trial	50 (MDD)	agomelatine (25 mg to 75 mg/d) + rTMS (5/w) for 8 weeks	Positive (↓ depressive symptoms)	↑ BDNF	↑ neurotransmitter content
[127]	randomized controlled trial	69 (MDD)	sertraline (50 mg/d) + tDCS (20 or 30 min sessions) for two weeks	Positive (↓ depressive symptoms)	BDNF n.c.	-
[128]	randomized controlled trial	66 (MDD)	paroxetine (20 mg/kg/d) + acupuncture for 4 weeks	Positive (↓ depressive symptoms)	↑ BDNF	↓ BDNF DNA methylation
[130]	randomized controlled trial	50 (post stroke depression)	escitalopram (25–50 mg/d) + REBT + Baduanjin exercise for 8 weeks	Positive (↓ depressive symptoms)	↑ BDNF	↓ neuroinflammation
[131]	randomized controlled trial	60 (depressive patients)	escitalopram (5–20 mg/d) + MCBT (120 min/w) for 8 weeks	Positive (↓ depressive symptoms)	↑ BDNF ↑ NGF	-
[135]	randomized controlled trial	40 (PD)	pramipexole (0.75 mg/d) + ZPG (13.5 g/d) for 12 weeks	Positive (↓ depressive symptoms)	↑ BDNF	↓ neuroinflammation
[136]	randomized controlled trial	31 (obese women with MDD on calorie restriction diet)	standard therapy + inulin (10 g/d) for 8 weeks	Positive (↓ depressive symptoms)	BDNF n.c.	-
[137]	randomized controlled trial	58 (children and adolescents depression)	standard therapy + omega-3 FA (20 mL/d) or omega-6 FA (20 mL/d) for 12 weeks	Positive (↓ depressive symptoms)–only with omega-3 FA	↑ BDNF–only with omega-3 FA	-
[138]	randomized controlled trial	118 (MDD)	standard therapy + NAC (2000 mg/d) for 12 weeks	Positive (↓ depressive symptoms)	BDNF n.c.	-
[141]	randomized controlled trial	30 (relapsing-remitting MS)	Brythonic training for 10 weeks	Positive (↓ depressive symptoms)	↑ BDNF	-

Abbreviations, acronyms, and symbols denote (in alphabetical order): BDNF, brain-derived neurotrophic factor; DNA, deoxyribonucleic acid; FA, fatty acids; GDNF, glial cell line-derived neurotrophic factor; MCBT, mindfulness-based cognitive behavioral therapy; MDD, major depressive disorder; MS, multiple sclerosis; NAC, N-acetylcysteine; n.c., no significant change; NGF, nerve growth factor; NT, neurotrophin; PD, Parkinson’s disease; REBT, rational emotive behavior therapy; Ref., reference (number); rTMS, repetitive transcranial magnetic stimulation; tDCS, transcranial direct current stimulation; ZPG, Zishen Pingchan granules; ↑, increased/upregulated; ↓, decreased/downregulated; -, not reported/not applicable.

As is obvious from the previously described combined protocols, all therapeutic strategies were based on standard (pharmacological) therapy, supplemented with additional methodology (dietary, exercise, etc.). However, the only combined therapy without antidepressant usage included the evaluation of NT system elements, which was presented by Farajnia [141]. The authors analyzed the impact of 10-week cognitive-aerobic exercise training (Brythonic) that combines aerobic movements while performing cognitive tasks and compared it to (only) aerobic exercise training in patients with relapsing-remitting multiple sclerosis with mild motor deficits. Although it significantly increased peripheral BDNF levels (app. 10%) and showed overall mood improvements, it did not significantly alter depression levels when compared to the exercise group. Still, depression improvement was evident when compared to control subjects.

## 5. Neurotrophin System in Bipolar Disorder

Distinct changes in mood and behavior during discrete time periods, characterized by recurrent episodes of depression and mania (or hypomania), are principal manifestations of bipolar disorder (BD). Since there is an evident disproportion of symptomatic time (75% of depressive episodes or symptoms), BD can be misdiagnosed as major depressive episodes [142]. Looking for a potential solution to face the issue of a high rate of misdiagnosis between MDD and BD in clinical practice, Yang and colleagues [143] performed an investigation of mature BDNF and pro-BDNF in postmortem human brain samples (parietal cortex and cerebellum) and peripheral tissues (liver and spleen) in patients with variously classified psychiatric disorders. The analysis of parietal cortex (Brodmann area 7) samples revealed a decrease in BDNF in both MDD and BD patients (app. 40%), as well as in schizophrenia patients (50%), with no change in cerebellar samples. Although the authors failed to propose the different results for BDNF and pro-BDNF as potential biomarkers for differential diagnosis (Table 4), the results of the study strongly supported the presence of neurotrophin system elements alterations in MDD, BD, and schizophrenia. Since decreased BDNF levels in older adults with BD, in BD-I (app. 30%) and not in BD-II, were previously documented by Soares and colleagues [144], it is not surprising that numerous attempts have been made to achieve therapeutic protocols to improve clinical features of BD along with an increase in BDNF. Thus, as the safest way to intervene under various medical conditions, dietary modifications that include omega-3 supplementation were employed by Gholipour and coworkers in BD patients [145]. The authors included 2 g/day of omega-3 supplements for 2 months, which resulted in a significant decline in depressive symptoms that were accompanied by a 50% increase in serum concentration of BDNF. The proposed explanation for improving BD symptoms may be related to the reduction in neuroinflammation and oxidative stress, as some of the widely accepted actions of omega-3 fatty acids [146].

A study by Huang and colleagues [147] evaluated the impact of the adjunctive action of dextromethorphan (30 mg/day) in BD patients who underwent a protocol with valproic acid (500 to 2500 mg daily–plasma levels between 50 and 100 μg/dL) for 12 weeks. Both applied agents showed improvements in the spectrum of mood disorders symptoms–depression (Hamilton Depression Rating Scale) and mania (Young Mania Rating Scale), as well as in cognitive functions. However, more convincing results regarding BDNF alterations were observed in the group of patients receiving the combination therapy (increase of 20%), whereas individual valproic acid administration did not result in a significant increase in BDNF. The beneficial effects observed with the therapy combination were attributed to neuroprotective (valproic acid) and anti-inflammatory (valproic acid and dextromethorphan) properties, while elevation in BDNF can be connected to both mechanisms. Unlike the promising results and optimistic conclusions achieved with dextromethorphan, an earlier trial of tianeptine (37.5 mg/day) as adjunctive therapy in BD patients receiving standard therapy (lithium, valproic acid, carbamazepine, risperidone, benzodiazepines) showed no significant improvement in BD symptoms [148]. The observed benefits were achieved only in a few aspects of short-term memory patterns. Also, evaluated serum BDNF levels showed no significant alterations throughout the protocol (24 weeks). A similar lack of clinical improvement with adjunctive therapy was observed with N-acetyl cysteine supplementation (2000 mg/day for 24 weeks) in a clinical trial performed by Panizzutti and collaborators [149]. In line with that, the authors also showed no significant alterations in BDNF and pro-inflammatory cytokines.

Lee and coworkers evaluated BD-II patients with comorbid alcohol dependence undergoing regular treatment with valproic acid (500 or 1000 mg/day–50–100 μg/mL in plasma) who were given add-on memantine (5 mg/day) for 12 weeks [150]. This noncompetitive NMDA receptor antagonist significantly improved the valproic acid action by means of reduction in depressive and manic symptoms and suicidal ideation. At the same time, the addition of memantine resulted in a significant increase in peripheral BDNF levels (up to 15%), which was accompanied by a decline in pro-inflammatory cytokines. The obtained results led to the conclusion that beneficial behavioral actions of memantine may be mediated via the anti-inflammatory action of BDNF. Interestingly, the authors also reported a significant increase in alcohol withdrawal among the estimated population. The impact of the BDNF Val66Met polymorphism in BD patients was summarized in the study by Lee and colleagues [151], based on previous clinical trials performed by the same investigator team (with a total of 355 patients). The summarized data showed a positive correlation between plasma BDNF levels and improvements in cognitive scores (also evaluated in this study), regardless of the BD subtype, but only in those with the Val/Met genotypes and BP-I. On the other hand, specific BD symptomatology (estimated by HDRS and YMRS) was significantly improved following applied treatments, but with no significant change in peripheral BDNF levels. Unfortunately, an in-depth analysis that could reveal some specific features, like BD subtypes, as well as a potential explanation of BDNF genotype variations, was not presented in this study. Another potential distinctive use of BDNF in clinical entities (subthreshold BD and BD-II) was estimated by Wang and coworkers [152]. According to the authors, patients with shorter and longer hypomania (BD-II) were classified using the Chinese Version of the Modified Schedule of Affective Disorder and Schizophrenia-Life Time, and the clinical evaluation (HDRS and YMRS) was performed along with determination of BDNF and cytokine levels, at the baseline and at the end of a 12-week course of standard therapy. The results presented showed significantly lower BDNF values in BD patients (shorter hypomania), when compared to the BD-II group (30%), from the beginning to the end of the trial, with no significant change in response to therapy within the groups. No significant change in response to therapy was also observed within the groups by means of cytokine profile, although baseline values of some pro-inflammatory markers (IL-6) were initially higher in the BD-II group of patients. All applied protocols resulted in clinical improvement, yet there was no explicit interpretation of the potential role of neurotrophins and/or cytokines in clinical outcome.

Although not formally classified as a category of neurotrophins, GDNF, due to its neuroprotective effect [153], was analyzed in a trial of Idemoto and investigators [154] to evaluate the potential differences between BD (n = 143) and MDD (n = 116) patients, as well as their response to lithium therapy. In both groups of patients, baseline values of peripheral BDNF were lower than in healthy controls (20% in BD). Following that finding, the authors proposed that serum GDNF levels could be considered as a potential biomarker for patients with mood disorders in remission or depressive states but also stated that serum GDNF levels are not suitable for differential diagnoses between MDD and BD since there was no significant difference between the two patient groups. Furthermore, according to the authors, serum GDNF levels could be considered a biomarker of the lithium response in BD patients. Following the therapeutic protocols, peripheral GDNF levels were reduced in BD patients with remission or depression, suggesting that lower GDNF levels in BD were associated with a good lithium response. The authors addressed the positive clinical outcome of therapy to the decline of GDNF that may contribute to improved glial response (astrocytes) and blood–brain barrier (BBB) function.

**Table 4:** Neurotrophin system alterations in bipolar disorder.

Ref.	Type of Study	Number of Patients	Therapeutic Protocol	Clinical Outcome	NT System Alterations	Proposed Mechanism
[143]	postmortem study	15 (postmortem analysis)	-	-	↓ BDNF ↑ pro-BDNF	-
[144]	cross-sectional study	118 (older adults)	-	-	↓ BDNF (in BD-I), n.c. in BD-II	-
[145]	randomized controlled trial	60	standard therapy + omega-3 supplements (2 g/d) for 2 months	Positive (↓ depressive symptoms)	↑ BDNF	↓ neuroinflammation ↓ oxidative stress
[147]	randomized controlled trial	96	valproic acid (500 to 2500 mg/d) + dextromethorphan (30 mg/d) for 12 weeks	Positive (↓ depressive symptoms ↓ mania symptoms)	↑ BDNF	↑ neuroprotection ↓ neuroinflammation
[148]	randomized controlled trial	36	standard therapy + tianeptine (37.5 mg/d)	n.c.	BDNF n.c.	-
[149]	randomized controlled trial	93	standard therapy + N-acetyl cysteine (2 g/d for 24 weeks)	n.c.	BDNF n.c.	-
[150]	randomized controlled trial	45 (with comorbid alcohol dependence)	standard therapy + memantine (5 mg/d)	Positive (↓ depressive symptoms ↓ mania symptoms ↑ alcohol withdrawal)	↑ BDNF	↓ neuroinflammation
[151]	randomized controlled trial	355	standard therapy + memantine (5 mg/d) or dextromethorphan (30–60 mg/d)	Positive (↓ depressive symptoms ↓ mania symptoms)–depending on BDNF genotype	BDNF n.c.	-
[152]	randomized controlled trial	89	standard therapy for 12 weeks	Positive (↓ depressive symptoms ↓ mania symptoms)	↑ BDNF (in BD-II)	neuroinflammation?
[154]	randomized controlled trial	143	standard therapy + lithium	Positive (↓ depressive symptoms ↓ mania symptoms)	↓ GDNF	glial response (astrocytes)? BBB function?
[155]	randomized controlled trial	70	standard therapy + MCBT	Positive (↓ depressive symptoms ↓ impulsive behavior ↑ cognitive functions)	↓ pro-BDNF	↑ neuroplasticity ↑ pro-BDNF/BDNF balance

Abbreviations, acronyms, and symbols denote (in alphabetical order): BBB, blood–brain barrier; BD-I, bipolar disorder type I; BD-II, bipolar disorder type II; BDNF, brain-derived neurotrophic factor; GDNF, glial cell line-derived neurotrophic factor; MCBT, mindfulness-based cognitive behavioral therapy; n.c., no significant change; NT, neurotrophin; pro-BDNF, precursor form of brain-derived neurotrophic factor; Ref., reference (number); ↑, increased/upregulated; ↓, decreased/downregulated; -, not reported/not applicable; ?, unverified mechanism.

A novel therapeutic approach as a modality of psychotherapy, mindfulness-based cognitive therapy (MCBT), was recently evaluated for its effect on the adolescent population with depressive episodes of BD [155]. The authors included 140 patients on standard pharmacotherapy (quetiapine 200–800 mg and serum lithium carbonate at 0.6–1.2 mmol/L), while 70 of them also underwent MCBT (two 90 min sessions per week over eight weeks). Different dimensions of impulsivity (motor impulsivity, cognitive impulsivity, and non-planning impulsivity) showed notable improvement after completing the MCBT protocol, which was accompanied by alleviated depressive and anxiety symptoms (related to self-injurious behavior). A significant decline in pro-BDNF serum levels (app. 10%) was also observed (a significant decrease was manifested even after four weeks). The authors suggested that pro-BDNF may serve as a potential biological marker for adolescents with BD, highlighting the decline of peripheral pro-BDNF for its potential neuroprotective effect by reshaping the pro-BDNF/BDNF balance.

## 6. Neurotrophin System in Anxiety Disorders

Even though anxiety disorders are often undervalued from the clinical point of view, they are widespread across the world, and as society develops, the disease burden of anxiety disorders tends to become an increasingly severe public health issue [156]. Anxiety disorders are classified into categories of generalized anxiety disorder (GAD), panic disorder, social anxiety disorder, and specific phobias. Common features are excessive and persistent fear, nervousness, and avoidance behaviors in response to perceived threats. In addition, patients often experience autonomic dysfunctions, according to DSM-5 criteria, which must persist for at least six months in generalized cases [157]. The potential role of the neurotrophin system in the generation and modulation of fear suggests a complex interplay between neuropsychological and behavioral events in response to the perception of dangerous stimuli [158], and numerous investigations have been conducted to estimate the neurotrophin system elements as potential biomarkers for the evaluation of fear-related disorders (Table 5).

Similar to MDD, the pharmacological approach to various medical conditions accompanied by increased anxiety levels is prevalent among other therapeutic procedures. Postoperative mood disorders, including anxiety and depression, are prevalent across surgical populations, with estimated rates varying across surgical specialties [159], and are also observed following elective cardiac valve surgery. To address that medical issue, Zhang and collaborators conducted a trial using a single low dose of esketamine (0.3 mg/kg) in those patients, following a standardized anesthesia protocol [78]. The results obtained in this study showed a significant reduction in anxiety symptoms. Biochemical analyses revealed that positive clinical outcomes were accompanied by decreased pro-inflammatory cytokines and increased BDNF (increase of 20–25% in postoperative days 1–3). According to the results obtained, the authors proposed that improvement in early postoperative anxiety may be addressed through modulation of neuroinflammation that involves neurotrophic signaling. The effects of esketamine on postoperative anxiety and depression were also evaluated by Bi and coworkers [160]. The authors administered an intravenous injection of esketamine during the induction period of general anesthesia at a higher dose than in the previous study (0.5 mg/kg) in female patients with thyroid cancer. Again, the authors reported an anxiolytic and antidepressant response accompanied by a significant increase in peripheral BDNF (by 20%, when compared to preoperative values) and 5-HT levels. To reveal the obvious dose-dependence of perioperatively administered esketamine, Luo and colleagues applied two doses (0.2 and 0.5 mg/kg) in patients undergoing non-cardiac thoracic surgery [161]. The results of this trial confirmed no significant impact of a lower esketamine dose, but positive clinical outcomes (decrease in anxiety, depression, and pain sensations), accompanied by increased BDNF (observed as early as at the end of surgery–20% for lower dose and 30% for higher dose, as well as three days after surgery–25% for lower dose and 40% for higher dose) and decreased neuroinflammatory and brain injury marker (IL-6, NSE, and S100β) levels, were observed with 0.5 mg/kg. The authors concluded that beneficial mechanisms could be attributed to the reduced production of pro-inflammatory and brain injury-related factors, as well as the generation of BDNF.

Anxiety episodes accompanied by surgical procedures were also evaluated in dentistry. Sivrikaya and coworkers analyzed the relationship between anxiety and BDNF serum level through impacted third molar surgery in patients under local anesthesia [162]. The influence of standard dental surgical procedures was manifested by decreased anxiety and depression, associated with increased BDNF levels (by 25%) after completing the procedure. However, statistical analysis did not confirm the correlation between the estimated parameters, which the authors explained as a situational episode. Another clinical entity that is repetitively evaluated with anxiety is functional dyspepsia, which was the objective of investigation by Liu et al. [163]. In this clinical trial, the authors evaluated the effects of tandospirone (30 mg/d for 8 weeks) on both gastrointestinal symptoms and behavioral correlates. Previously increased anxiety and decreased peripheral BDNF levels, when compared to healthy controls, were successfully reversed by tandospirone to a significant anxiolytic effect accompanied by increased BDNF (up to 25%) and IL-10 and decreased IL-6 levels. The obtained results indicated that the therapeutic effect of tandospirone on functional dyspepsia may be related to the modulation of neurotrophins and inflammatory cytokines. Aside from comorbidities, anxiety disorders also appear in clinical practice in the form of treatment-resistant generalized anxiety and/or social anxiety disorders. In a trial with the principal limitation of the small sample size (12 patients), Glue and researchers evaluated the effect of ascending doses of ketamine (0.25, 0.5, and 1 mg/kg) and midazolam (0.01 mg/kg), administered at weekly intervals, on anxiety level along with BDNF concentrations [164]. Unlike midazolam, the beneficial effects of ketamine occurred within an hour of dosing and persisted for up to 1 week, depending on dose (with maximal effect of 30%, after 120 min). Simultaneously, serum BDNF concentrations declined over time and were similar across all treatments. Another specific population of patients was evaluated for the potential impact of neurotrophin system elements on anxiety level. Thus, Wang and colleagues analyzed the effect of upper-limb robot-assisted therapy (combined with pneumatic gloves–PG) on upper-limb function (UL-RAT&PG) and mood alterations in young and middle-aged stroke patients [165]. A significant anxiolytic effect (along with an antidepressant) was observed following advanced rehabilitation methodology (5 days a week, for 4 consecutive weeks), when compared to conventional therapy, which was accompanied by increased BDNF serum levels (by 40%). Those results correlated with EEG and MRI findings that confirm increased brain plasticity, based on which the authors potentiated the role of neurotrophic factors. The interesting observations were presented in the earlier trial by Phillips and coworkers, who simultaneously analyzed anxiety and BDNF levels in women with weight recovery in anorexia nervosa [166]. Namely, although the patients initially (during anorexia nervosa) showed a negative correlation between BDNF and anxiety levels, after successful therapeutic protocols (mainly with SSRIs), this relationship was absent, suggesting that it has been adjusted at a different level.

Not surprisingly, as is more or less specific to anxiety etiology, dietary protocols have been employed in the treatment of a broad spectrum of anxiety disorders. The impact of polyphenols applied in the form of epigallocatechin-3-gallate (EGCG, 350 mg) and curcumin (665 mg) as a supplementation to standard therapy on mood disorder symptomology, along with the potential impact on peripheral BDNF, was estimated in a randomized controlled trial [167]. The positive clinical outcome, by means of decreased anxiety level, accompanied by a significant antidepressant effect, was confirmed after 4 and 8 weeks of protocol. However, the authors reported that serum BDNF concentrations did not significantly increase over time, suggesting that the supplementation did not influence these levels. Still, the authors postulated that significant alterations of BDNF could be achieved with a different experimental design and an estimated population. In contrast, the dietary intervention with micro-encapsulated Lacticaseibacillus rhamnosus HN001 in healthy elderly subjects (60–80 y.) maintained the same anxiety scores, as well as depressive scores, and did not change BDNF levels; however, it showed improvement in brain resting state functional connectivity in regions involved in visual processing and perception following a 6-week protocol [168]. Still, the authors believe their results show promise for improving cognitive impairment and promoting healthy aging. In line with this, Climent and coworkers [169] analyzed the potential effects of psychobiotic protection of nutritional supplements and probiotics in patients undergoing hemodialysis using a composite capsule of probiotics containing live bacteria–Bifidobacterium breve, Bifidobacterium animalis lactis, and Lactobacillus paracasei. Again, there were no significant alterations in BDNF levels; only the authors reported a decrease in anxiety and depression levels. They highlighted the importance of the gut–brain axis, but the complex interplay between the gut microbiome and human health still requires further exploration. Even more convincing benefits for anxiety symptoms and BDNF levels were presented in the 12-week trial of Haghighat et al. [170] following both synbiotic and probiotic supplementation in hemodialysis patients. Notably, synbiotic supplementation (15 g of prebiotics and 5 g of probiotics containing Lactobacillus acidophilus, Bifidobacterium bifidum, Bifidobacterium lactis, and Bifidobacterium longum) resulted in a greater improvement in the HADS depression score and serum level of BDNF protein (30% by the end of protocol) compared to the probiotic (5 g of probiotics as in the synbiotic group with 15 g of maltodextrin). In contrast, probiotic supplementation with Bifidobacterium longum (for 6 weeks) in patients with irritable bowel syndrome did not significantly alter the anxiety level or peripheral BDNF levels but achieved significant antidepressant action [171]. Dietary intervention to treat mood disorders was also employed by using wheat germ consumption in subjects with type 2 diabetes mellitus [172]. Although the authors reported a significant increase in BDNF levels (by 40%) accompanied by improvement in depression and stress-related features, there was no change in anxiety levels following the 12-week trial. Another dietary intervention using a traditional Chinese herb (anxiolytic and hypnotic effects), Xinkeshu, was presented by Ma et al. [173]. In this randomized placebo-controlled trial, the authors administered Xinkeshu (4 × 3 tablets daily for 12 weeks) to coronary artery disease patients after successful revascularization (n = 30), since they usually have increased anxiety levels. This adjuvant therapeutic approach resulted in anxiolytic and antidepressant effects, which were accompanied by a significant increase in peripheral NT-3 levels. The authors attributed those beneficial outcomes to the “neurotrophin hypothesis” that confirms the role of neurotrophins in the development and survival of neurons and promotes cell connections and plasticity previously decreased in response to stress. Finally, the framework for dietary interventions could be completed with the trial that evaluated intermittent fasting effects on anxiety levels in healthy volunteers [174]. The authors presented that the fasting protocol (abstinence from solid food for at least 24 h weekly for 8 weeks) did not significantly alter results obtained in the anxiety scale nor peripheral BDNF levels (Table 5).

Although considered modern and/or non-standard methods, non-invasive transcranial stimulation techniques are widely used in the treatment of different aspects of anxiety disorders. The intention of researchers to explore new therapeutic options for mild cognitive impairment patients resulted in a trial to estimate transcranial photobiomodulation (t-PBM) effects. De Oliveira and collaborators performed a trial to evaluate the impact of t-PBM (the treatment comprised 20 min sessions, 24 sessions over 60 days) on various behavioral patterns and serum BDNF levels in adults over 50 years [175]. Although the main goal was achieved by an increase in cognitive functions, the authors also reported a decrease in anxiety patterns, along with increased BDNF levels (up to 20%), which lasted up to 5 months (probably due to BDNF antioxidant action). There were no statistically significant changes in depressive state between the t-PBM and placebo groups at any evaluation point. Based on that, they proposed the t-PBM application as an adjunctive treatment for preventing neurodegenerative diseases and improving mood disorders. The research group by Xiao employed rhythmic low-field magnetic stimulation to test the potential improvement of mood disorders in MDD patients (n = 22) [176]. This transcranial stimulation methodology was performed as rhythmic alpha stimulation or rhythmic delta stimulation for 6 weeks (5 sessions per week). Clinical benefits were manifested by the reduction in mood disorder severity (anxiety and depressive disorder); only the more prominent anxiolytic effect was observed in patients with rhythmic delta stimulation and was accompanied by increased peripheral BDNF levels (app. 30%). The authors proposed that a change in BDNF levels (at week 2) was the most strongly predicted clinical response, since the process of repetitive magnetic stimulation may improve brain oscillatory activity and connectivity, and consequently, increase neuroplasticity. The benefits of rTMS were also confirmed in patients with a primary increased anxiety level, i.e., generalized anxiety disorder, by Lu et al. [177]. Bilateral low-frequency rTMS over the dorsolateral prefrontal cortex (5 days per week, for two weeks) resulted in a significant, almost two-fold increase in serum BDNF levels, accompanied by a significant reduction in anxiety symptoms. The authors offered an explanation that different frequencies of rTMS stimulate BDNF synthesis in the cerebral cortex, which in turn increases neuronal regeneration, synaptic plasticity, and emotional regulation. In contrast, an earlier trial that employed cranial electrotherapy stimulation (three times per week for 8 weeks in 20 min sessions) in healthy postmenopausal female volunteers, although it resulted in anxiolytic and antidepressant effects, did not significantly affect BDNF and NGF peripheral levels [178].

An interesting alternative approach was applied to investigate the effects of internet and wearable device training on limb function recovery in stroke patients [179]. Aside from routine post-hospital follow-up guidance, the authors employed internet remote home rehabilitation guidance combined with wearable device training (12 weeks), which resulted in a significant anxiolytic effect (along with an antidepressant), but also in increased levels of serum neurotrophins (BDNF, NT-3, and NGF). The authors proposed the neuroregenerative action of estimated neurotrophins as the beneficial mechanism. Those findings may be of special importance in the light of the fact that serum BDNF levels did not correlate with anxiety levels in poststroke anxiety patients, although a significant (negative) correlation was observed in poststroke depression [180]. One earlier intervention considering potential lifestyle changes that could affect anxiety level via alterations in neurotrophin system elements was performed by Glazachev and colleagues [181]. The authors employed repeated sessions of systemic passive hyperthermia (whole-body infrared heating with head-out) in young healthy men, which resulted in a significant increase in serum BDNF (up to 25%). Increased BDNF was accompanied by reduced anxiety levels. The quantitative analysis revealed that intermittent light-intensity exercise, performed in parallel, did not significantly affect estimated parameters. An expectedly beneficial long-lasting (nine-month) multidisciplinary protocol was applied in the study conducted by Bartlett et al. in individuals with preclinical Huntington’s disease (HD) [182]. The preventive protocol consisted of periodized aerobic and resistance training (twice weekly for one hour) and comprised resistance, endurance, and high-intensity interval training; computerized cognitive training (three times weekly for one hour) to target various aspects of cognitive functions; and social events (for three months) in small groups to encourage social engagement. Although with no clear clinical outcome (improvement in anxiety and depression levels was not significant), the authors reported evidence that the multidisciplinary protocol slowed the reduction in hypothalamic volume loss, as well as maintained peripheral BDNF levels in individuals with preclinical HD. Still, due to the small sample size in this pilot study (n = 18), they suggested that larger randomized controlled trials may confirm the clinical benefits of this therapeutic approach.

**Table 5:** Neurotrophin system alterations in anxiety disorders.

Ref.	Type of Study	Number of Patients	Therapeutic Protocol	Clinical Outcome	NT System Alterations	Proposed Mechanism
[78]	randomized controlled trial	71 (cardiac valve surgery)	standard anesthesia + esketamine (0.3 mg/kg)	Positive (↓ anxiety)	↑ BDNF	↓ neuroinflammation
[160]	randomized controlled trial	40 (thyroid cancer surgery)	standard anesthesia + esketamine (0.5 mg/kg)	Positive (↓ anxiety)	↑ BDNF	-
[161]	randomized controlled trial	87 (non-cardiac thoracic surgery)	standard anesthesia + esketamine (0.2 and 0.5 mg/kg)	Positive (↓ anxiety)	↑ BDNF	↓ neuroinflammation ↓ brain injury
[162]	randomized controlled trial	55 (healthy adults)	impacted third molar extraction under local anesthesia	Positive (↓ anxiety)	↑ BDNF	-
[163]	randomized controlled trial	60 (functional dyspepsia)	tandospirone (30 mg/d for 8 weeks)	Positive (↓ anxiety)	↑ BDNF	↓ neuroinflammation
[164]	randomized controlled trial	12 (treatment-resistant generalized anxiety)	ketamine (0.25, 0.5, 1 mg/kg) or midazolam (0.01 mg/kg)	Positive (↓ anxiety)	↓ BDNF	-
[165]	randomized controlled trial	20 (young and middle-aged stroke patients)	UL-RAT&PG (5 d/w, 4 weeks)	Positive (↓ anxiety)	↑ BDNF	↑ brain plasticity
[166]	randomized controlled trial	16 (females with anorexia nervosa)	SSRIs	Positive (↓ anxiety)	BDNF n.c.	
[167]	randomized controlled trial	28 (adults)	standard therapy + EGCG (350 mg) and curcumin (665 mg) for 8 weeks	Positive (↓ anxiety)	BDNF n.c.	-
[168]	randomized controlled trial	58 (elderly population)	encapsulated and non-encapsulated *Lacticaseibacillus rhamnosus* HN001	n.c.	BDNF n.c.	-
[169]	randomized controlled trial	10 (hemodialysis patients)	composite probiotic supplementation (for 6 months)	Positive (↓ anxiety)	BDNF n.c.	-
[170]	randomized controlled trial	50 (hemodialysis patients)	synbiotic and probiotic supplementation (for 12 weeks)	Positive (↓ anxiety)	↑ BDNF	-
[171]	randomized controlled trial	22 (irritable bowel syndrome)	probiotic supplementation with *Bifidobacterium longum* (for 6 weeks)	n.c.	BDNF n.c.	-
[172]	randomized controlled trial	40 (diabetes mellitus type 2)	wheat germ (20 g/d for 12 weeks)	n.c.	↑ BDNF	-
[173]	randomized placebo-controlled trial	30 (coronary artery disease patients after successful revascularization)	Xinkeshu (4 × 3 tablets/d for 12 weeks)	Positive (↓ anxiety)	↑ NT-3	↑ neuroplasticity
[174]	nonrandomized controlled clinical trial	22 (healthy adults)	intermittent fasting (24 h/w, for 8 weeks)	n.c.	BDNF n.c.	-
[175]	randomized controlled trial	47 (healthy adults)	t-PBM (20 min, 24 sessions over 60 days)	Positive (↓ anxiety)	↑ BDNF	↓ oxidative stress?
[176]	randomized controlled trial	11 (MDD)	rhythmic delta stimulation (5 sessions/w) for 6 weeks	Positive (↓ anxiety)	↑ BDNF	↑ neuroplasticity
[177]	randomized controlled trial	28 (generalized anxiety disorder)	low-frequency rTMS over dorsolateral prefrontal cortex (5 d/w, for 2 weeks)	Positive (↓ anxiety)	↑ BDNF	↑ neurotranmitters production ↑ neuroplasticity
[178]	randomized controlled trial	25 (postmenopausal healthy female volunteers)	cranial electrotherapy stimulation (3/w for 8 weeks)	Positive (↓ anxiety)	BDNF n.c. NGF n.c.	-
[179]	randomized controlled trial	40 (stroke patients)	internet and wearable device training	Positive (↓ anxiety)	↑ BDNF ↑ NGF ↑ NT-3	↑ neuroregeneration
[181]	randomized controlled trial	14 (healthy subjects)	systemic passive hyperthermia	Positive (↓ anxiety)	↑ BDNF	-
[182]	non-randomized pilot clinical trial	18 (preclinical Huntington’s disease)	combined (exercise, cognitive tasks, social programs) for 9 months	n.c.	↑ BDNF	↑ hypothalamus volume

Abbreviations, acronyms, and symbols denote (in alphabetical order): BDNF, brain-derived neurotrophic factor; EGCG, epigallocatechin gallate; MDD, major depressive disorder; n.c., no significant change; NGF, nerve growth factor; NT, neurotrophin; NT-3, neurotrophin-3; Ref., reference (number); rTMS, repetitive transcranial magnetic stimulation; SSRIs, selective serotonin reuptake inhibitors; t-PBM, transcranial photobiomodulation; UL-RAT&PG, upper-limb robot-assisted therapy combined with pneumatic gloves; ↑, increased/upregulated; ↓, decreased/downregulated; -, not reported/not applicable; ?, unverified mechanism.

Aside from any intervention, the importance of the BDNF gene polymorphism in generalized anxiety disorder has been evaluated by Gonzales-Castro et al. [183]. This case–control study showed the association between BDNF Val66Met polymorphism and the risk of developing generalized anxiety disorder (evaluated using 4 inheritance models); however, it found no association between this polymorphism and the symptomatology of anxiety.

## 7. Neurotrophin System in Schizophrenia

Schizophrenia represents a mental disease characterized by a complex and incomprehensible mental condition that creates alienation from reality, usually accompanied by progressive deficits in working memory, attention, and executive functioning [184]. Since the etiology of schizophrenia remains unclear and neuroprotective factors (including neurotrophins) are most likely related to the pathogenesis of schizophrenia, reasonable efforts have been made to reveal the algorithms that could connect symptom severity and efficiency of treatment methods with neurotrophin system alterations (Table 6).

As expected, most data on the impact of neurotrophin system elements in schizophrenia patients was obtained from clinical trials that evaluated the effects of various antipsychotics (from different generations), since the pharmacological approach is dominant and first-line therapy for this psychiatric issue. A recent clinical trial by Zhang [185] evaluated the impact of 8-week treatment with magnesium valproate (0.25–1 g/d) alone and in combination with quetiapine (0.15–0.75 g/d) in patients with schizophrenia. The analysis included clinical outcome, as well as BDNF and glial fibrillary acidic protein (GFAP) levels in peripheral blood. Presented results showed that magnesium valproate (a commonly used clinical antiepileptic drug), which enhances the expression of aminobutyric acid and 5-HT receptors (by inhibiting the selectivity of aminobutyric acid transferase) when administered concomitantly with quetiapine (a D2 antagonist that inhibits the reuptake of norepinephrine transporter), although with different pharmacological effects, exerted a synergistic effect. The benefits of combined therapy were manifested by improvement in psychiatric symptoms accompanied by diminished cognitive dysfunction (with relatively mild side effects). At the same time, the impact on neurotrophic factors appeared as an increment on serum BDNF (app. 20%) and a reduction in GFAP levels. A potential connection between estimated parameters could be addressed to neuroprotective features of BDNF (repair of neuronal oxidative damage [186]), as well as GFAP’s role in regulating astrocyte functions [187]. Another comparative clinical trial with therapeutics in the treatment of schizophrenia was presented by Jena and investigators [188]. In this randomized controlled clinical trial, the authors employed (following 4 weeks without medication) olanzapine (10 mg/d) or lurasidone (80 mg/d) for 6 weeks. The outcome analysis revealed that both drugs alleviated the symptoms of schizophrenia (although olanzapine was better tolerated). The extensive exploration of neurotrophic factor alterations showed that the serum levels of all estimated parameters (BDNF, NGF, and NT-3) significantly increased. The impact of monotherapy on neurotrophin levels did not differ for NGF and NT-3, but serum BDNF levels were significantly higher in olanzapine-treated patients (three-fold increase vs. two-fold increase in lurasidone). Another comparison of antipsychotic drugs’ effects in first-episode schizophrenia, specifically risperidone and its main active metabolite, paliperidone, was presented by Wu and contributors [189]. Although both employed protocols with two D2 and 5HT2 antagonists, improved clinical symptoms, as well as cognitive functioning, a negative correlation between the reduction rate of the PANSS score and the increase in serum BDNF level (20% increase for both drugs) after the treatment was found only in the paliperidone group. A response to monotherapy in schizophrenia patients was analyzed by Krivoy and colleagues [190] in a trial that evaluated the effects of clozapine. According to the criteria defined as a reduction of <20% in pre-clozapine treatment PANSS score, responders’ group (significant clinical improvement) expressed higher serum levels of BDNF (up to 25%) and glutamate. Interestingly, glutamate levels correlated with the psychosis severity only in clozapine responders (probably due to improvement in glutamate metabolism, although not explicitly documented in this study). It is worth noticing that other explored neurotrophic factors, GDNF and NGF, did not significantly differ between the groups of responders and non-responders. In contrast, the clinical trial performed on drug-naive first-episode patients with schizophrenia treated with risperidone (4–6 mg/d, for 3 months), conducted by Wu and coworkers [191], also showed clinical improvement but failed to detect significant changes in BDNF levels. This finding is in line with the earlier report indicating that BDNF did not significantly change after a 30-month trial with standard therapeutic protocols in a multicenter study, suggesting that this neurotrophic factor could not be considered as a plausible biological marker for predicting schizophrenia relapse [192]. Finally, one of the unofficial add-ons to standard therapeutic protocols in schizophrenia, cannabis, was used in the clinical trial presented by George and coworkers [193]. The authors showed that the combined antipsychotic therapy and cannabis use disorder significantly increased peripheral BDNF levels (app. 15%), though the clinical outcome was controversial. Namely, they observed an increase in both positive and negative (not significant) symptoms, but with a notable sub-dosage in the combined group, allowing the authors to reach decisive concluding remarks.

However, not all accurate pharmacological protocols showed significant alterations in neurotrophin system elements along with antipsychotics. In addition to standard protocols, Strzelecki and collaborators [194] included sarcosine (a glycine transporter–inhibitor of GlyT-1) as a supplement (2 g/d) for six months. As previously declared, despite no significant alterations in peripheral BDNF levels, sarcosine add-on was significantly more effective when compared to the placebo group in terms of negative symptoms, general psychopathology, and the total PANSS score.

Similar to other frequent and serious medical issues, a significant effort was directed toward addressing schizophrenia by changing substantial lifestyle characteristics, such as interventions in physical activity. Based on their previous results indicating that physical activity can improve neurocognition in individuals with schizophrenia [195], the research group led by Bang-Kittilsen [196] analyzed the impact of physical activity characteristics on clinical outcome and neurotrophin status in patients with schizophrenia on standard therapy. The one group of patients was exposed to high-intensity interval training (85–95% of the maximal heart rate), while the other group underwent low-intensity active video gaming for 12 weeks. Although the authors presented extensive statistical analysis, there was no data to confirm significant alterations in clinical outcomes or in peripheral BDNF and pro-BDNF levels after the completion of therapeutic protocols, as well as after follow-up testing. However, the authors declared that baseline BDNF moderated the effect of physical exercise on neurocognition and accounted for a small part of the inter-individual variation. Physical activity has also been confirmed for its benefits as an additional protocol in schizophrenia patients (after the first episode) in a study performed by Nuechterlein and researchers [197]. This investigator’s group joined a physical activity program for cognitive training and showed that, when applied simultaneously, aerobic exercise significantly enhanced the impact of cognitive training on cognitive and functional outcomes, with a positive correlation to exercise protocol duration. As the authors stated, BDNF gain tended to predict the amount of cognitive improvement, although it did not reach significance.

Another frequent lifestyle intervention, aside from the standard pharmacological approach, in schizophrenia patients in the last decade was based on dietary protocols. An initial report was presented by Chung and colleagues [198], before dietary interventions, considering the connection between fatty acid content and neurotrophin system elements (BDNF) in the peripheral blood of patients treated with paliperidone extended release (3–12 mg/day). As the primary focus of this study, the authors reported that psychopathology was positively correlated with polyunsaturated fatty acid levels and negatively correlated with saturated fatty acid levels after completing the therapeutic protocol (8 weeks). Interestingly, BDNF levels showed a negative correlation with polyunsaturated fatty acids and a positive correlation with saturated and monounsaturated fatty acids. This study was followed by a trial performed in treatment-resistant schizophrenia patients who received alpha-lipoic acid as an add-on [199]. Alpha-lipoic acid, known for its ability to reverse NMDA receptor hypofunction in addition to dopamine receptor blockade activity, has also been confirmed to possess neurotrophic and antioxidant characteristics [200]. Supplementation with alpha-lipoic acid showed beneficial clinical outcomes (improvement in negative symptoms) and decreased oxidative stress markers, but it had a negative effect on BDNF levels.

**Table 6:** Neurotrophin system alterations in schizophrenia.

Ref.	Type of Study	Number of Patients	Therapeutic Protocol	Clinical Outcome	NT System Alterations	Proposed Mechanism
[185]	randomized controlled trial	156	magnesium valproate (0.25–1 g/d) and/or magnesium + valproate (0.25–1 g/d) quetiapine (0.15–0.75 g/d) for 8 weeks	Positive (↓ psychiatric symptoms ↑ cognitive functions ↑ life quality)	↑ BDNF	↑ oxidative damage repair ↓ glial function
[188]	randomized controlled trial	101	olanzapine (10 mg/d) or lurasidone (80 mg/d) for 6 weeks	Positive (↓ psychiatric symptoms ↑ patients’ functioning)	↑ BDNF ↑ NGF ↑ NT-3	↑ multiple neuroprotective effects
[189]	randomized controlled trial	94	risperidone or paliperidone for 12 weeks	Positive (↓ psychiatric symptoms ↑ cognitive functions)	↑ BDNF	-
[190]	cross-sectional, observational study	89	clozapine (minimum 18 weeks)	Positive (↓ psychiatric symptoms)	↑ BDNF GDNF n.c. NGF n.c.	↑ glutamate metabolism?
[191]	observational longitudinal study	183	risperidone (4–6 mg/d) for 3 months	Positive (↓ psychiatric symptoms)	BDNF n.c.	↑ oxidative equilibrium
[193]	cross-sectional observational study	40	standard therapy + cannabis	Negative (↑ psychiatric symptoms)	↑ BDNF	-
[194]	randomized controlled and parallel trial	57	standard therapy + sarcosine (2 g/d) for 6 months	Positive (↓ psychiatric symptoms)	BDNF n.c.	-
[195]	randomized controlled trial	71	standard therapy + physical exercise (high and low intensity)	n.c.	BDNF n.c. pro-BDNF n.c.	-
[197]	randomized controlled trial	47	standard therapy + cognitive therapy + physical exercise	Positive (↓ psychiatric symptoms ↑ cognitive functions)	BDNF n.c.	-
[198]	randomized controlled trial	75	paliperidone (3–12 mg/d) for 8 weeks	Positive (↓ psychiatric symptoms ↑ cognitive functions)	BDNF n.c.	-
[199]	pilot randomized controlled trial	20	standard therapy + alpha lipoic acid	Positive (↓ negative symptoms)	BDNF n.c.	-
[201]	sham-controlled clinical trial	43	paliperidone (3–6 mg/d, *per os*) + rTMS (20 min, 5/w) for 4 weeks	Positive (↓ negative symptoms)	↑ BDNF	↑ brain blood flow ↑ neurotransmitters activity ↑ growth factors production
[202]	randomized controlled trial	64	standard therapy + rTMS or cTBS (15 min, 5/w) for 4 weeks	Positive (↓ psychiatric symptoms)	↑ BDNF ↑ GDNF	↑ neuroplasticity
[203]	randomized controlled trial	40	standard therapy + ECT or MST (10/w for 4 weeks)	Positive (↓ psychiatric symptoms)	↑ BDNF ↓ pro-BDNF	↓ neuronal death ↑ synaptic plasticity ↑ hippocampal volume
[204]	pilot study	12	standard therapy + rTMS (20 min, 5/w) for 4 weeks	n.c.	BDNF n.c.	-
[205]	randomized controlled trial	30	standard therapy + EA (3/w for 4 weeks)	n.c.	BDNF n.c.	-
[206]	randomized controlled trial	35	CRT (40 h over 4 months)	n.c. in psychiatric symptoms–depending on BDNF genotype ↑ cognitive functions	BDNF n.c.	-
[207]	randomized controlled trial	40	CRT (40 h over 4 months)	n.c. in psychiatric symptoms ↑ cognitive functions	↓ BDNF methylation	↑ neuroplasticity
[208]	randomized controlled trial	30	standard therapy + NF (2/w for 3 months)	Positive (↓ psychiatric symptoms)	↑ BDNF	-

Abbreviations, acronyms, and symbols denote (in alphabetical order): BDNF, brain-derived neurotrophic factor; CRT, cognitive remediation therapy; cTBS–continuous theta burst stimulation; EA, electroacupuncture; ECT, electroconvulsive therapy; GDNF, glial cell line-derived neurotrophic factor; MDD, major depressive disorder; MST, magnetic seizure therapy; n.c., no significant change; NF, neurofeedback; NGF, nerve growth factor; NT, neurotrophin; NT-3, neurotrophin-3; pro-BDNF, precursor form of brain-derived neurotrophic factor; Ref., reference (number); rTMS, repetitive transcranial magnetic stimulation; ↑, increased/upregulated; ↓, decreased/downregulated; -, not reported/not applicable; ?, unverified mechanism.

The importance of transcranial stimulation methodology in the treatment of schizophrenia is confirmed by the fact that it represents the second most frequent therapeutic protocol, after pharmacological, in addressing this serious medical issue. Not surprisingly, as probably one of the most comfortable methods for the patients, magnetic stimulation has been widely employed in the treatment of schizophrenia. Zhai and coworkers evaluated the potential benefits of repetitive transcranial magnetic stimulation (rTMS) on the negative symptoms of chronic schizophrenia, along with serum BDNF levels [201]. A total of 43 patients, all simultaneously administered paliperidone (3–6 mg/d, per os), underwent rTMS treatment (20 min five times a week) for four weeks. The applied combined protocol, when compared to the sham group (only pharmacotherapy), showed significant clinical improvement by means of negative symptoms of schizophrenia, which was accompanied by an increase in serum BDNF levels (app. 30%). According to results obtained in this study, the authors proposed that rTMS in patients with chronic schizophrenia boosts neurotransmitter biological activity, enhances neurological blood flow, cell-to-cell interaction, and promotes growth factor production (including neurological BDNF expression), which in turn improves negative symptoms. Another research group recently analyzed the response to rTMS and continuous theta-burst stimulation (cTBS), both for 15 min, 5 times/week for 4 weeks, in schizophrenia patients with auditory verbal hallucinations on standard pharmacological therapy [202]. The authors presented that both applied transcranial stimulation protocols resulted in substantial symptom improvement (positive, negative, and general symptoms) while noting that cTBS was more efficient. At the same time, serum levels of BDNF and GDNF also increased following treatment in both groups (more pronounced in the cTBS group), while GABA and glutamate levels remained unchanged. The authors proposed that transcranial stimulation could enhance neuroplasticity within prefrontal brain circuits, possibly through the enhancement of neurotransmission pathways, including GABAergic and glutamatergic systems. Another comparison between two transcranial stimulation methods in schizophrenia patients was made in a study by Li and collaborators [203]. The authors applied magnetic seizure therapy (MST) or electroconvulsive therapy-ECT (10 sessions per week, over four weeks; 17 and 23 patients, respectively) along with standard therapy. Both treatments improved clinical outcome, but MST was more efficient in some specific cognitive aspects (language score). Also, both protocols resulted in significant decreases in pro-BDNF (30% for ECT and 20% for MST), while BDNF levels were increased (almost two-fold in both protocols). Interestingly, ECT significantly increased hippocampal volumes, while MST had no effect. The authors speculated that alterations in mBDNF/pro-BDNF balance were potentially critical to neuronal death and synaptic plasticity, finally resulting in total hippocampal volume changes. In contrast to the results presented in previous studies, the investigator team of Mendes-Filho [204], while estimating the effects of rTMS over the supplementary motor area in patients with schizophrenia with obsessive-compulsive symptoms in a pilot study, showed that rTMS did not significantly change the clinical outcomes, as well as peripheral BDNF levels, after treatment and on follow-up. It should be noted that the group of authors led by Sun [205] estimated the potential benefits of electroacupuncture (EA) on clinical symptoms in schizophrenic patients. However, EA (12 sessions, 3 times a week over a period of 4 weeks) did not significantly alter clinical symptoms and did not affect peripheral BDNF levels. Still, the authors reported that EA showed a positive correlation with memory improvement (Table 6).

In addition, it is worth noticing that psychotherapy protocols were also employed in the treatment of schizophrenia patients during the last decade. Thus, Penades and colleagues performed cognitive remediation treatment (CRT, 40 h of treatment, consisting of 1 h sessions two or three times a week over 4 months) in patients with schizophrenia along with standard pharmacological therapy [206]. However, no significant improvement was observed after completion of the protocol. Also, there was no change in peripheral BDNF levels. On the other hand, the authors noticed a significant positive interaction between the serum BDNF levels and the different BDNF genotypes (the Val/Val group showed significantly higher serum levels after the CRT). The same group of authors [207] reached similar conclusions when they analyzed the connection between cognitive improvement and BDNF gene methylation in schizophrenic patients following CRT. The authors proposed that peripheral methylation levels of the BDNF gene may be a key factor involved in neuroplasticity regulation. To evaluate if neurofeedback (NF) can be effective as an add-on therapy in schizophrenia rehabilitation programs, Markiewicz and researchers [208] performed a trial with NF training sessions (twice a week for three months) along with standard therapy. The applied therapeutic design resulted in significant improvement by means of clinical outcome, as well as in increased BDNF serum levels (by 20–25%).

## 8. Neurotrophin System in Posttraumatic Stress Disorder

A spectrum of complex psychiatric conditions that arise in response to exposure to traumatic events and significantly affect mental well-being is commonly characterized as posttraumatic stress disorder (PTSD) [209]. With notably high common underlying pathophysiological mechanisms, PTSD is accompanied by an elevated risk of comorbid psychiatric illnesses, including depressive disorders. Dell’Oste and colleagues [210] conducted an interesting study aimed at distinguishing bipolar disorder patients with PTSD from those in a current MDD episode by analyzing blood BDNF content in two different compartments—intraplatelet and platelet-poor plasma—in an effort to define reliable biochemical criteria for clinical evaluation. A total of 20 subjects in the euthymic phase with PTSD showed no difference in BDNF content between the two compartments, while patients in the MDD episode expressed significantly lower intraplatelet BDNF levels when compared to healthy controls, which appeared along with the increased score in HAM-D when compared to PTSD patients. At the same time, by maintaining all quantified BDNF levels as in healthy controls, patients with PTSD showed a higher score in the Impact of Event Scale-Revised in comparison to patients with MDD episodes (Table 7).

In the study conducted by Jager and collaborators [211], the authors evaluated the effects of programmed physical activity on serum BDNF levels along with clinical parameters in PTSD patients (with previously confirmed lower BDNF levels when compared to healthy subjects). The principal result obtained in this study showed that a single session of high-intensity interval training (HIIT) was able to significantly increase serum BDNF levels (app. 15%) in individuals with PTSD, but this effect was transient (expiring after only 30 min). This was accompanied by improvement in the results obtained in the Posttraumatic Stress Disorder Checklist for DSM-5. Low-intensity interval training showed no immediate significant effect, nor any after completion of a 12-day protocol, on BDNF levels and clinical outcomes. Crombie and investigators [212] presented another study that evaluates the potential beneficial effects of physical activity in the treatment of PTSD, this time with the focus on improving the efficacy of exposure-based therapies, which are hypothesized to work via the mechanisms of fear extinction learning to exposure for PTSD. The study included a female population with PTSD (n = 35) who underwent a 3-day protocol (fear acquisition, extinction, and extinction recall) followed by either moderate-intensity aerobic exercise or a light-intensity control condition. The applied protocols resulted in an increase in peripheral BDNF level, which was accompanied by reduced threat expectancy; however, no specific mechanism was proposed to connect the obtained results.

Difede and researchers [213] employed a total of 192 military service members who acquired Iraq or Afghanistan war combat-related PTSD to evaluate the impact of enhancing exposure therapy [214]. This methodology was applied in the form of virtual reality and/or imaginal exposure with a cognitive enhancer [215]. Both applied protocols showed similar clinical improvement with no difference between the treatment groups. The authors also observed the moderating effect of the BDNF Val66Met polymorphism following the administration of a cognitive enhancer (D-cycloserine). Participants carrying the BDNF Met66 allele (Val/Met and Met/Met combined) showed significant clinical improvement with the applied cognitive enhancer, suggesting the role of BDNF polymorphism in PTSD. The importance of BDNF polymorphism was also confirmed in the study performed by Dai and investigators [216] on the population affected by the flood in China. Gene analyses obtained 17 years after the initial incident showed that Met carriers for BDNF rs6265 were at a higher risk of developing PTSD and exhibited more severe PTSD symptoms compared to Val/Val homozygotes.

**Table 7:** Neurotrophin system alterations in posttraumatic stress disorder.

Ref.	Type of Study	Number of Patients	Therapeutic Protocol	Clinical Outcome	NT System Alterations	Proposed Mechanism
[210]	randomized comparative study	20	-	-	n.c.	-
[211]	randomized controlled trial	40	high and low intensity interval training	Positive (↓ PTSD symptoms) only after HIIT first session	↑ BDNF (only after the first HIIT session)	-
[212]	randomized controlled trial	35	acquisition, extinction, and extinction recall protocol followed by aerobic exercise	Positive (↓ threat expectancy)	↑ BDNF	-
[214]	randomized controlled trial	192	enhancing exposure therapy (prolonged and virtual)	Positive (↓ PTSD symptoms)	responsiveness depending on BDNF polymorphism	-

Abbreviations, acronyms, and symbols denote (in alphabetical order): BDNF, brain-derived neurotrophic factor; HIIT, high-intensity interval training; n.c., no significant change; NT, neurotrophin; PTSD, post-traumatic stress disorder; Ref., reference (number); ↑, increased/upregulated; ↓, decreased/downregulated; -, not reported/not applicable.

## 9. Neurotrophin System in Autism Spectrum Disorders

Autism spectrum disorder (ASD) is a neurobiological disorder, influenced by both genetic and environmental factors (affecting the developing brain), characterized by specific deficits in social communication and the presence of restricted interests and repetitive behaviors [217]. Therefore, it is not surprising that alterations in the neurotrophin system, which is strongly involved in neurodevelopment across all phases, have prompted investigators to pursue a potential causal therapeutic approach in clinical trials (Table 8).

An interventional pilot study conducted by Allan and colleagues [218] on children with ASD aimed to evaluate the impact of a modified ketogenic diet for 4 months on behavioral patterns. Although it started with 47 patients, only 11 completed the protocol, and the results showed increased sociability accompanied by reduced plasma BDNF levels (app. 75%). More detailed analyses revealed variable responses in BDNF-associated miRNAs (a reduction in miR-134 and miR-132, an increase in miR-375, and no change in miR-125b) and an anti-inflammatory outcome (decrease in IL-1β and IL-12p70 levels) associated with the ketogenic diet [219]. Upon completing the data analysis related to gut microbiota, the authors proposed that the ketogenic diet positively influenced behavioral symptoms in children with ASD not only through the direct effect of ketones on metabolic activity in the brain, but also through increased gut microbial diversity, with the prominent role of increased production of butyrate in the gut. Butyrate easily passes the blood–brain barrier and directly affects the inflammatory state, as well as through interactions with certain receptors, neurotransmitters, and/or BDNF miRNAs [220], thus confirming that the gut microbiome can affect the expression of epigenetic factors.

In a search of genetic BDNF polymorphism as a potential predictive factor in the confirmed fragile X syndrome (the most common monogenic cause of ASD [221]), AlOlaby and collaborators [222] confirmed that some specific forms of BDNF genotype significantly correlated with response to SSRI protocols (sertraline 2.5–5 mg/d for 8 months) in ASD subjects. In this double-blind placebo-controlled clinical trial focusing on genes related to the serotonin pathway and positive outcomes in young children with ASD (with confirmed fragile X syndrome) treated with sertraline, the val/val BDNF genotype was most responsive to the effects of sertraline according to Clinical Global Impression Scale-Improvement criteria when compared to the val/met and met/met genotypes.

An interesting application of a non-standard therapeutic method for treating ASD was recently reported by an Egyptian research team [223]. The authors estimated the influence of low-level laser acupuncture (LLLA) on the sample of ASD children with a language-stimulating environment, when compared to neurotypical children without LLLA (n = 15 + 15), by means of ASD severity, language performance, the expression of miR-320, and the level of BDNF in this observational longitudinal study. Two sessions per week for 12 consecutive weeks of LLLA administration resulted in significant clinical improvement (enhanced language performance and decreased ASD severity) and decreased BDNF levels (up to 20%) when compared to the parent-assisted language stimulation group, which is in line with the similar, beneficial, effect also achieved with LLLA that explained the potential role of BDNF in more details [224]. However, the estimation of miR-320 (nerve growth regulation factor) showed no significant change between the two experimental groups. Indeed, both ASD groups after therapeutic protocols had significantly lower miR-320 levels when compared to healthy controls. Alterations in gray matter and cortical thickness, addressed to estimated biomarkers [225], were proposed as a potential mechanism that underlies observed clinical changes.

**Table 8:** Neurotrophin system alterations in autism spectrum disorders.

Ref.	Type of Study	Number of Patients	Therapeutic Protocol	Clinical Outcome	NT System Alterations	Proposed Mechanism
[218]	interventional pilot study	11	modified ketogenic diet	Positive (↑ sociability)	↓ BDNF	↓ neuroinflammation
[222]	placebo-controlled clinical trial	25	sertraline (2.5–5 mg/d) for 8 months	↑ response to sertraline	responsivity depends on BDNF genotype	↑ serotonin pathways
[223]	observational longitudinal study	15	LLLA	Positive (↑ language performance, ↓ ASD severity)	↓ BDNF	alterations in gray matter and cortical thickness?
[226]	interventional pilot study	16	rTMS, tDCS	Positive (↓ ASD severity)	↓ BDNF	↑ MAPK and PI3K/AKT1 pathways

Abbreviations, acronyms, and symbols denote (in alphabetical order): AKT1, serine/threonine kinase 1; ASD, autism spectrum disorder; BDNF, brain-derived neurotrophic factor; LLLA, low-level laser acupuncture; MAPK, mitogen-activated protein kinase; NT, neurotrophin; PI3K, phosphoinositide 3-kinase; Ref., reference (number); rTMS, repetitive transcranial magnetic stimulation; tDCS, transcranial direct current stimulation; ↑, increased/upregulated; ↓, decreased/downregulated; ?, unverified mechanism.

Non-pharmacological therapeutic approaches to ASD in the last decade also included the transcranial stimulation methods. In order to intervene at the level of aberrant neural connectivity and intra-cortical inhibitory dysfunction, the key features of autism, a research group of Cuban investigators [226] applied non-invasive brain stimulation techniques–low frequency rTMS and tDCS in twenty sessions for one month in children (age 5–13, n = 16), with or without pharmacotherapy (carbamazepine, risperidone, methylphenidate, or haloperidol). The effect of brain stimulation was manifested by a significant reduction in ASD severity, which was accompanied by a trend towards reduced BDNF serum levels (app. 15%).

## 10. Neurotrophin System in Sleep Disorders

Sleep disorders represent a heterogeneous group of conditions that disturb normal sleep patterns and affect overall health, safety, and quality of life [227]. In searching for management options for sleep disorders, various research groups have focused on the potential role of neurotrophin system elements as biomarkers to predict the outcomes of numerous therapeutic approaches (Table 9).

The randomized comparative study by Kumar and colleagues [228] analyzed the effects of 12-week treatment with zolpidem and clobazam in patients with insomnia. Patients were evaluated for sleep quality, quality of life, and serum BDNF levels. Patients with insomnia treated by zolpidem and clobazam showed significant improvements in terms of insomnia severity, sleep quality, and quality of life, accompanied by higher BDNF levels when compared to control subjects. The observed BDNF increase (app. 10% for zolpidem and 20% for clobazam) indicates a possible association with sleep regulation, because of the postulated neuroprotective mechanism. Although not primarily aimed at the evaluation of sleep disorders, a clinical trial performed by Wang and collaborators [229] to investigate repeated ketamine infusions (0.5 mg/kg) in MDD and BD patients revealed that this therapeutic approach was positively associated with high serum BDNF levels (increase by 50% and 75% after 13 and 26 days, respectively), as well as early sleep disturbance improvement (24 h after the first ketamine injection). Interestingly, the authors noted that this initial reaction may be useful for predicting the repeated-dose ketamine antidepressant effect. Since the authors did not estimate the downstream mechanisms involved in this positive response to ketamine, the observed alterations were attributed to the previously described beneficial effects of ketamine as a circadian regulator, as well as its impact on the Notch, MAPK [230], and glutamatergic signaling pathways [231].

More detailed insight into BDNF impact on sleep disorders was presented in the study by Deuschle and coworkers [232]. Namely, the authors offered an analysis of various types of sleep disorders, as well as a precise classification of sleep stages to estimate the connection with BDNF level alterations. The results of this observational study, which classified sleep disorders as primary insomnia (35 patients), restless legs syndrome (31 patients), idiopathic hypersomnia (17 patients), and narcolepsy (10 patients), during sleep stages N1, N2, N3, and REM sleep, revealed a specific algorithm of BDNF alterations. Serum levels of BDNF were related to the proportion of sleep stage N3 and REM sleep, with no significant difference among specific sleep disorders, which was consistent with the assumption that sleep stage N3 is involved in the regulation of neuroplasticity.

The effects of In the randomized controlled trial by Golmohammadi and collaborators [233] employed the MIND (Mediterranean-DASH Intervention for Neurodegenerative Delay) diet on type 2 diabetic women with insomnia, as previously described [234]. The results showed that the 3-month MIND diet was efficient in significantly improving sleep quality, depression, and anxiety compared to the control (low-calorie diet) group. At the same time, BDNF was significantly higher (app. 50%) in the women who underwent the MIND low-calorie diet, while cortisol levels were reduced. The authors proposed that the beneficial effects of the MIND diet could be attributed to previously described antioxidant and anti-inflammatory effects of this dietary protocol [235].

**Table 9:** Neurotrophin system alterations in sleep disorders.

Ref.	Type of Study	Number of Patients	Therapeutic Protocol	Clinical Outcome	NT System Alterations	Proposed Mechanism
[228]	randomized comparative study	60	zolpidem (5 mg/d) or clobazam (5 mg/d) for 12 weeks	Positive (↓ insomnia severity, ↑ sleep quality, ↑ quality of life)	↑ BDNF	↑ neuroprotection
[229]	open-label study	127	ketamine (repeated infusions of 0.5 mg/kg)	Positive (↓ sleep disruption, ↓ depression)	↑ BDNF	↑ circadian activity ↑ MAPK pathway ↑ glutamatergic signaling
[232]	observational study	93	-	-	↓ BDNF (N3 stage and REM)	↑ neuroplasticity
[233]	randomized controlled trial	22	MIND low-calorie diet (12 weeks)	Positive (↑ sleep quality, ↓ anxiety, ↓ depression)	↑ BDNF	↓ inflammation ↓ oxidative stress ↓ cortisol
[236]	randomized clinical trial	87	acupuncture (8 weeks)	Positive (↑ total sleep time)	↑ BDNF (initially low baseline)	-

Abbreviations, acronyms, and symbols denote (in alphabetical order): BDNF, brain-derived neurotrophic factor; MAPK, mitogen-activated protein kinase; MIND, Mediterranean-DASH Intervention for Neurodegenerative Delay; N3, non-rapid eye movement sleep stage 3; NT, neurotrophin; Ref., reference (number); REM, rapid eye movement sleep; ↑, increased/upregulated; ↓, decreased/downregulated; -, not reported/not applicable.

A randomized clinical trial presented by Liou and colleagues [236] performed on a very specific population that usually expresses sleeping disorders–cancer survivors [237], evaluated the effects of acupuncture and cognitive behavioral therapy comparatively. The results showed no difference in the final clinical outcome depending on the therapeutic approach. However, cancer survivors with insomnia and initially low baseline BDNF who underwent acupuncture significantly increased serum BDNF levels (app. 20%), which were accompanied by increased total sleep time. The 8-week cognitive behavioral therapy applied in this study showed no significant impact on BDNF levels nor on sleep parameters expressed through the insomnia severity index [236].

Although not formally classified as a primary psychiatric disorder, obstructive sleep apnea has also been found to exacerbate numerous behavioral functions, such as cognition, which can appear alongside various pathological states, including Parkinson’s disease [238]. In a prospective observational study, Kaminska and researchers [239] included PD patients with obstructive sleep apnea to evaluate the impact on clinical outcome and BDNF serum levels. The applied methodology used for the quantification of sleep disorder confirmed that the elevated BDNF levels correlated with increased sleepiness, and the authors proposed systemic inflammation mechanisms to be key factors for the determination of sleep disorder severity.

## 11. Neurotrophin System in Obsessive-Compulsive Disorder

Obsessive-compulsive disorder (OCD) has not been analyzed as a specific psychiatric entity in the context of the impact of neurotrophin system element alterations in the available literature sources from the last decade. Several publications described compulsive behavior as a part of the clinical appearance of behavioral patterns that are mainly accompanied by addiction pathology, especially alcohol withdrawal and/or opioid use disorder.

## 12. Conclusions

In the last decade, a respectable number of psychiatric clinical trials have included evaluations of neurotrophin system elements as additional parameters to follow therapeutic procedures and their outcomes. Their valuable results revealed the potential benefits of these still-exploratory markers (rather than biomarkers) for in-depth analyses of their protocols, including efficiency, prognosis, etc.

While we are still gathering new data on this topic, some general remarks could be made. Firstly, there is an obvious disproportion among clinical entities that involved NT system analysis, which were predominantly presented with mood disorders, while some other pathologies were insufficiently analyzed regarding those markers’ alterations. Even among the trials grouped according to pathology and/or therapeutic protocols, there is an evident heterogeneity by means of participants (sample size, disease severity and duration, age of participants, comorbidities, etc.) and treatments (drugs, doses, duration of protocols, subsequent enrollment of other therapies), which often limits the possibility of reaching reliable conclusions.

Even more important is the disproportion for the selection of evaluated NT system elements. Namely, BDNF was the most extensively evaluated neurotrophin (the other neurotrophins were rarely addressed), and only a limited number of studies included the estimation of neurotrophin receptors (only TrkB), with no available data regarding the receptors that mediate the negative actions of neurotrophins (as explained in Section 3). After all, most of the investigations overlooked numerous downstream mechanisms impacted by the NT system that might be substantially important for the clinical outcomes.

Named observations could, at the same time, be considered a potential explanation for the observed differences and consequent interpretations, given the role of the NT system, as a unique complex system in psychiatric disorders.

## 13. Future Directions

Future research should employ longitudinal and multimodal approaches for better insight into the causal relationships between neurotrophin system element dynamics and psychiatric pathology. Integrating the network of peripheral biomarkers with neuroimaging, genetic, and epigenetic data may provide a more comprehensive understanding of neurotrophin-related mechanisms. There is a need to explore complex models that capture interactions among multiple components of the neurotrophic system and related pathways.

Translational investigations could also be useful to improve the estimation of neurotrophins modulation accompanied by specific therapeutic protocols, including pharmacological, behavioral, and other neuromodulatory interventions, with special attention to individualized treatment strategies, and provide a more detailed approach in the field of precision psychiatry.

## Data Availability

No new data were created or analyzed in this study. Data sharing is not applicable to this article.

## Abbreviations

The following abbreviations are used in this manuscript:

NGF	Nerve Growth Factor
BDNF	Brain Derived Neurotrophic Factor
NT	Neurotrophin
Trk	Tropomyosin Receptor Kinase
MMP	Matrix Metalloproteinases
PAI-1	Plasminogen Activator Inhibitor-1
PLC-γ1	Phospholipase C-gamma 1
MAPK	Mitogen-activated Protein Kinase
ERK	Extracellular Signal-regulated Kinase
PI3K	Phosphatidylinositol 3-kinases
Akt	Protein Kinase B
Nrf2	Nuclear Factor Erythroid 2-related Factor 2
CREB	cAMP Response Element-binding Protein
IRS	Insulin Receptor Substrate
SOD	Superoxide Dismutase
PKC	Protein kinase-C
TRAF6	TNF Receptor Associated Factor 6
NRIF	Neurotrophin Receptor Interacting Factor
JNK	c-Jun N-terminal Kinase
NF-κB	Nuclear Factor Kappa B
TNF-α	Tumor Necrosis Factor-alpha
IL	Interleukin
MDD	Major Depressive Disorder
5-HT	5-hydroxytryptamine (serotonin)
CaMKII	Calcium/calmodulin-dependent protein kinase II
wb-BDNF	Whole Blood Brain Derived Neurotrophic Factor
NMDA	N-Methyl-D-aspartate
SSRIs	Selective Serotonin Reuptake Inhibitors
CBT	Cognitive Behavioral Therapy
PSD	Post-schizophrenic Depression
GDNF	Glial Cell Line-derived Neurotrophic Factor
HPA axis	Hypothalamic–pituitary–adrenal axis
rTMS	Repetitive Transcranial Stimulation
tDCS	Transcranial Direct Current Stimulation
TRD	Treatment Resistant Depression
MCBT	Mindfulness-based Cognitive Therapy
REBT	Rational Emotive Behavior Therapy
PD	Parkinson’s Disease
FA	Fatty Acid
NAC	N-acetylcysteine
BD	Bipolar Disorder
BBB	Blood–brain Barrier
GAD	Generalized Anxiety Disorder
NSE	Neuron-specific Enolase
UL-RAT&PG	Upper-limb Robot-assisted Therapy and Pneumatic Gloves
EGCG	Epigallocatechin-3-gallate
HADS	Hospital Anxiety and Depression Scale
t-PBM	Transcranial Photobiomodulation
HD	Huntington’s Disease
GFAP	Glial Fibrillary Acidic Protein
PANSS	Positive and Negative Syndrome Scale
cTBS	Continuous Theta-burst Stimulation
MST	Magnetic Seizure Therapy
ECT	Electroconvulsive Therapy
EA	Electroacupuncture
CRT	Cognitive Remediation Treatment
NF	Neurofeedback
PTSD	Posttraumatic Stress Disorder
HIIT	High-intensity Interval Training
ASD	Autism Spectrum Disorder
LLLA	Low-level Laser Acupuncture
MIND diet	Mediterranean-DASH Intervention for Neurodegenerative Delay Diet
OCD	Obsessive-compulsive Disorder
LTP	Long-term Potentiation

## References

[1] Patel V., Saxena S., Lund C., Thornicroft G., Baingana F., Bolton P., Chisholm D., Collins P.Y., Cooper J.L., Eaton J. (2018). The Lancet Commission on Global Mental Health and Sustainable Development. Lancet.

[2] (2022). Global, Regional, and National Burden of 12 Mental Disorders in 204 Countries and Territories, 1990–2019: A Systematic Analysis for the Global Burden of Disease Study 2019. Lancet Psychiatry.

[3] First M.B., Tasman A., Riba M.B., Alarcón R.D., Alfonso C.A., Kanba S., Lecic-Tosevski D., Ndetei D.M., Ng C.H., Schulze T.G. (2024). Psychiatric Classification. Tasman’s Psychiatry.

[4] Nakamura S. (2022). Integrated Pathophysiology of Schizophrenia, Major Depression, and Bipolar Disorder as Monoamine Axon Disorder. Front. Biosci.-Sch..

[5] Rădulescu I., Drăgoi A., Trifu S., Cristea M. (2021). Neuroplasticity and Depression: Rewiring the Brain’s Networks through Pharmacological Therapy (Review). Exp. Ther. Med..

[6] Murck H., Martin C.R., Preedy V.R., Patel V.B., Rajendram R. (2025). Neuroendocrine and Autonomic Dysregulation in Affective Disorders. Handbook of the Biology and Pathology of Mental Disorders.

[7] Sălcudean A., Bodo C.-R., Popovici R.-A., Cozma M.-M., Păcurar M., Crăciun R.-E., Crisan A.-I., Enatescu V.-R., Marinescu I., Cimpian D.-M. (2025). Neuroinflammation—A Crucial Factor in the Pathophysiology of Depression—A Comprehensive Review. Biomolecules.

[8] Huang H., Luo Z., Min J., Luo W., Zhou X., Wang C. (2025). Targeting Neuroinflammation in Schizophrenia: A Comprehensive Review of Mechanisms and Pharmacological Interventions. Int. Immunopharmacol..

[9] Steardo L., D’Angelo M., Monaco F., Di Stefano V., Steardo L. (2025). Decoding Neural Circuit Dysregulation in Bipolar Disorder: Toward an Advanced Paradigm for Multidimensional Cognitive, Emotional, and Psychomotor Treatment. Neurosci. Biobehav. Rev..

[10] Grotzinger A.D., Werme J., Peyrot W.J., Frei O., De Leeuw C., Bicks L.K., Guo Q., Margolis M.P., Coombes B.J., Batzler A. (2026). Mapping the Genetic Landscape across 14 Psychiatric Disorders. Nature.

[11] Hobbs M., Moltchanova E., Marek L., Yogeeswaran K., Milfont T.L., Deng B., Sibley C.G. (2025). Environmental Influences on Mental Health: Eight-Year Longitudinal Data Show a Bi-Directional Association between Residential Mobility and Mental Health Outcomes. Health Place.

[12] Wingo T.S., Liu Y., Gerasimov E.S., Vattathil S.M., Wynne M.E., Liu J., Lori A., Faundez V., Bennett D.A., Seyfried N.T. (2022). Shared Mechanisms across the Major Psychiatric and Neurodegenerative Diseases. Nat. Commun..

[13] Levy M.J.F., Boulle F., Steinbusch H.W., Van Den Hove D.L.A., Kenis G., Lanfumey L. (2018). Neurotrophic Factors and Neuroplasticity Pathways in the Pathophysiology and Treatment of Depression. Psychopharmacology.

[14] Numakawa T., Kajihara R. (2025). The Role of Brain-Derived Neurotrophic Factor as an Essential Mediator in Neuronal Functions and the Therapeutic Potential of Its Mimetics for Neuroprotection in Neurologic and Psychiatric Disorders. Molecules.

[15] Hernández-del Caño C., Varela-Andrés N., Cebrián-León A., Deogracias R. (2024). Neurotrophins and Their Receptors: BDNF’s Role in GABAergic Neurodevelopment and Disease. Int. J. Mol. Sci..

[16] Von Bohlen Und Halbach O., Klausch M. (2024). The Neurotrophin System in the Postnatal Brain—An Introduction. Biology.

[17] Popova N.K., Ilchibaeva T.V., Naumenko V.S. (2017). Neurotrophic Factors (BDNF and GDNF) and the Serotonergic System of the Brain. Biochemistry.

[18] Shen Z., Zhu J., Yuan Y., Ren L., Qian M., Lin M., Cai M., Zhang Z., Shen X. (2019). The roles of brain-derived neurotrophic factor (BDNF) and glial cell line-derived neurotrophic factor (GDNF) in predicting treatment remission in a Chinese Han population with generalized anxiety disorder. Psychiatry Res..

[19] Al-Qudah M.A., Al-Dwairi A. (2016). Mechanisms and Regulation of Neurotrophin Synthesis and Secretion. Neurosciences.

[20] Kowiański P., Lietzau G., Czuba E., Waśkow M., Steliga A., Moryś J. (2018). BDNF: A Key Factor with Multipotent Impact on Brain Signaling and Synaptic Plasticity. Cell. Mol. Neurobiol..

[21] Mitre M., Mariga A., Chao M.V. (2017). Neurotrophin Signalling: Novel Insights into Mechanisms and Pathophysiology. Clin. Sci..

[22] Bogacheva P.O., Molchanova A.I., Pravdivceva E.S., Miteva A.S., Balezina O.P., Gaydukov A.E. (2022). ProBDNF and Brain-Derived Neurotrophic Factor Prodomain Differently Modulate Acetylcholine Release in Regenerating and Mature Mouse Motor Synapses. Front. Cell. Neurosci..

[23] Wang M., Xie Y., Qin D. (2021). Proteolytic Cleavage of proBDNF to mBDNF in Neuropsychiatric and Neurodegenerative Diseases. Brain Res. Bull..

[24] Lei M., Liu Q., Nie J., Huang R., Mei Y., Pan D., Chen Y., Liu W. (2024). Impact and Mechanisms of Action of BDNF on Neurological Disorders, Cancer, and Cardiovascular Diseases. CNS Neurosci. Ther..

[25] Suleymanova E., Karan A. (2025). The Plasminogen Activation System in the Central Nervous System: Implications for Epilepsy and Neuropsychiatric Disorders. Int. J. Mol. Sci..

[26] Angelucci F., Veverova K., Katonová A., Vyhnalek M., Hort J. (2023). Serum PAI-1/BDNF Ratio Is Increased in Alzheimer’s Disease and Correlates with Disease Severity. ACS Omega.

[27] Chen Z.-Y., Ieraci A., Teng H., Dall H., Meng C.-X., Herrera D.G., Nykjaer A., Hempstead B.L., Lee F.S. (2005). Sortilin Controls Intracellular Sorting of Brain-Derived Neurotrophic Factor to the Regulated Secretory Pathway. J. Neurosci..

[28] Al-Yozbaki M., Acha-Sagredo A., George A., Liloglou T., Wilson C.M. (2020). Balancing Neurotrophin Pathway and Sortilin Function: Its Role in Human Disease. Biochim. Biophys. Acta BBA—Rev. Cancer.

[29] Haddad Y., Adam V., Heger Z. (2017). Trk Receptors and Neurotrophin Cross-Interactions: New Perspectives Toward Manipulating Therapeutic Side-Effects. Front. Mol. Neurosci..

[30] Enkavi G. (2026). Molecular Determinants of Signal Transduction in Tropomyosin Receptor Kinases. FEBS Open Bio.

[31] Ahmed F., Zapata-Mercado E., Rahman S., Hristova K. (2021). The Biased Ligands NGF and NT-3 Differentially Stabilize Trk-A Dimers. Biophys. J..

[32] Conroy J.N., Coulson E.J. (2022). High-Affinity TrkA and P75 Neurotrophin Receptor Complexes: A Twisted Affair. J. Biol. Chem..

[33] Covaceuszach S., Konarev P.V., Cassetta A., Paoletti F., Svergun D.I., Lamba D., Cattaneo A. (2015). The Conundrum of the High-Affinity NGF Binding Site Formation Unveiled?. Biophys. J..

[34] Zanin J.P., Montroull L.E., Volosin M., Friedman W.J. (2019). The P75 Neurotrophin Receptor Facilitates TrkB Signaling and Function in Rat Hippocampal Neurons. Front. Cell. Neurosci..

[35] Meeker R., Williams K. (2015). The P75 Neurotrophin Receptor: At the Crossroad of Neural Repair and Death. Neural Regen. Res..

[36] Toader C., Serban M., Munteanu O., Covache-Busuioc R.-A., Enyedi M., Ciurea A.V., Tataru C.P. (2025). From Synaptic Plasticity to Neurodegeneration: BDNF as a Transformative Target in Medicine. Int. J. Mol. Sci..

[37] Siddi C., Balla J., Fadda P., Dedoni S. (2026). Epigenetic Regulation of Trk Receptors and Neurotrophic Signalling in Neuroblastoma: Mechanisms, Plasticity, and Therapeutic Opportunities. Int. J. Mol. Sci..

[38] Lao-Peregrin C., Xiang G., Kim J., Srivastava I., Fall A.B., Gerhard D.M., Kohtala P., Kim D., Song M., Garcia-Marcos M. (2024). Synaptic Plasticity via Receptor Tyrosine Kinase/G-Protein-Coupled Receptor Crosstalk. Cell Rep..

[39] Singh A.A., Katiyar S., Song M. (2025). Phytochemicals Targeting BDNF Signaling for Treating Neurological Disorders. Brain Sci..

[40] Bruna B., Lobos P., Herrera-Molina R., Hidalgo C., Paula-Lima A., Adasme T. (2018). The Signaling Pathways Underlying BDNF-Induced Nrf2 Hippocampal Nuclear Translocation Involve ROS, RyR-Mediated Ca2+ Signals, ERK and PI3K. Biochem. Biophys. Res. Commun..

[41] Gray N.E., Farina M., Tucci P., Saso L. (2022). The Role of the NRF2 Pathway in Maintaining and Improving Cognitive Function. Biomedicines.

[42] Guo W., Nagappan G., Lu B. (2018). Differential Effects of Transient and Sustained Activation of BDNF-TrkB Signaling. Dev. Neurobiol..

[43] Ji Y., Lu Y., Yang F., Shen W., Tang T.T.-T., Feng L., Duan S., Lu B. (2010). Acute and Gradual Increases in BDNF Concentration Elicit Distinct Signaling and Functions in Neurons. Nat. Neurosci..

[44] Qiu D., Mao L., Kikuchi S., Tomita M. (2004). Sustained MAPK Activation Is Dependent on Continual NGF Receptor Regeneration. Dev. Growth Differ..

[45] Wang X., Wang Q., Hou L., Wei G., He C., Li H., Liu L. (2025). Advances in ERK Signaling Pathway in Traumatic Brain Injury: Mechanisms and Therapeutic Potential. Neurochem. Res..

[46] Ateaque S., Merkouris S., Wyatt S., Allen N.D., Xie J., DiStefano P.S., Lindsay R.M., Barde Y. (2022). Selective Activation and Down-regulation of Trk Receptors by Neurotrophins in Human Neurons Co-expressing TrkB and TrkC. J. Neurochem..

[47] Duman R.S., Deyama S., Fogaça M.V. (2021). Role of BDNF in the Pathophysiology and Treatment of Depression: Activity-dependent Effects Distinguish Rapid-acting Antidepressants. Eur. J. Neurosci..

[48] Bhujbal S.P., Hah J.-M. (2025). Advances in JNK Inhibitor Development: Therapeutic Prospects in Neurodegenerative Diseases and Fibrosis. Arch. Pharm. Res..

[49] Kamada H., Nito C., Endo H., Chan P.H. (2007). Bad as a Converging Signaling Molecule between Survival PI3-K/Akt and Death JNK in Neurons after Transient Focal Cerebral Ischemia in Rats. J. Cereb. Blood Flow Metab..

[50] Azman K.F., Zakaria R. (2022). Recent Advances on the Role of Brain-Derived Neurotrophic Factor (BDNF) in Neurodegenerative Diseases. Int. J. Mol. Sci..

[51] Bothwell M. (2019). Recent Advances in Understanding Context-Dependent Mechanisms Controlling Neurotrophin Signaling and Function. F1000Research.

[52] Singh S., Singh T.G. (2020). Role of Nuclear Factor Kappa B (NF-κB) Signalling in Neurodegenerative Diseases: An Mechanistic Approach. Curr. Neuropharmacol..

[53] Liu D., Zhong Z., Karin M. (2022). NF-κB: A Double-Edged Sword Controlling Inflammation. Biomedicines.

[54] Charlton T., Prowse N., McFee A., Heiratifar N., Fortin T., Paquette C., Hayley S. (2023). Brain-Derived Neurotrophic Factor (BDNF) Has Direct Anti-Inflammatory Effects on Microglia. Front. Cell. Neurosci..

[55] Düsedau H.P., Kleveman J., Figueiredo C.A., Biswas A., Steffen J., Kliche S., Haak S., Zagrebelsky M., Korte M., Dunay I.R. (2019). P75^NTR^ Regulates Brain Mononuclear Cell Function and Neuronal Structure in *Toxoplasma* Infection-induced Neuroinflammation. Glia.

[56] Farina L., Minnone G., Alivernini S., Caiello I., MacDonald L., Soligo M., Manni L., Tolusso B., Coppola S., Zara E. (2022). Pro Nerve Growth Factor and Its Receptor p75NTR Activate Inflammatory Responses in Synovial Fibroblasts: A Novel Targetable Mechanism in Arthritis. Front. Immunol..

[57] Yang C., Ding H., Yu M., Zhou F., Han C., Liang R., Zhang X., Zhang X., Meng F., Wang S. (2022). proBDNF/p75NTR Promotes Rheumatoid Arthritis and Inflammatory Response by Activating Proinflammatory Cytokines. FASEB J..

[58] Li H., Liu T., Sun J., Zhao S., Wang X., Luo W., Luo R., Shen W., Luo C., Fu D. (2023). Up-Regulation of ProBDNF/p75NTR Signaling in Spinal Cord Drives Inflammatory Pain in Male Rats. J. Inflamm. Res..

[59] Demuth H., Hosseini S., Düsedeau H.P., Dunay I.R., Korte M., Zagrebelsky M. (2023). Deletion of p75NTR Rescues the Synaptic but Not the Inflammatory Status in the Brain of a Mouse Model for Alzheimer’s Disease. Front. Mol. Neurosci..

[60] Yang C., Liang R., Liu Y., Meng F., Zhou F., Zhang X., Ning L., Wang Z., Liu S., Zhou X. (2024). Upregulation of proBDNF/p75NTR Signaling in Immune Cells and Its Correlation with Inflammatory Markers in Patients with Major Depression. FASEB J..

[61] Yilanli M., Kaur J. (2026). Healthcare Professional Burnout. StatPearls.

[62] Ormel J., Kessler R.C., Schoevers R. (2019). Depression: More Treatment but No Drop in Prevalence How Effective Is Treatment? And Can We Do Better?. Curr. Opin. Psychiatry.

[63] Wang Y., Qin C., Chen H., Liang W., Liu M., Liu J. (2025). Global, Regional, and National Burden of Major Depressive Disorders in Adults Aged 60 Years and Older from 1990 to 2021, with Projections of Prevalence to 2050: Analyses from the Global Burden of Disease Study 2021. J. Affect. Disord..

[64] Wang H., Yang Y., Pei G., Wang Z., Chen N. (2023). Neurotrophic Basis to the Pathogenesis of Depression and Phytotherapy. Front. Pharmacol..

[65] Forster A., Rodrigues J., Sperlich B., Hewig J. (2026). The Use of Behavioral Reconsolidation Interference in Depressive Disorders. A Double-Blinded Randomized Controlled Experimental Registered Report. Psychophysiology.

[66] Popovic D., Weyer C., Dwyer D.B., Griffiths S.L., Lalousis P.A., Barnes N.M., Vetter C., Neuner L.-M., Buciuman M.-O., Sarisik E. (2026). Multivariate Brain-Blood Signatures in Early-Stage Depression and Psychosis. JAMA Psychiatry.

[67] Lee B.-H., Park Y.-M., Hwang J.-A., Kim Y.-K. (2021). Variable Alterations in Plasma Erythropoietin and Brain-Derived Neurotrophic Factor Levels in Patients with Major Depressive Disorder with and without a History of Suicide Attempt. Prog. Neuropsychopharmacol. Biol. Psychiatry.

[68] Chiou Y.-J., Huang T.-L. (2016). Serum Brain-Derived Neurotrophic Factors in Taiwanese Patients with Drug-Naïve First-Episode Major Depressive Disorder: Effects of Antidepressants. Int. J. Neuropsychopharmacol..

[69] Williams M.S., Ngongang C.K., Ouyang P., Betoudji F., Harrer C., Wang N.-Y., Ziegelstein R.C. (2016). Gender Differences in Platelet Brain Derived Neurotrophic Factor in Patients with Cardiovascular Disease and Depression. J. Psychiatr. Res..

[70] Caldieraro M.A., Vares E.A., Souza L.H., Spanemberg L., Guerra T.A., Wollenhaupt-Aguiar B., Ferrari P., Nierenberg A.A., Fleck M.P. (2017). Illness Severity and Biomarkers in Depression: Using a Unidimensional Rating Scale to Examine BDNF. Compr. Psychiatry.

[71] Luo L., Mao X., Fu W., Song X., Zhou L. (2025). Serum Levels of CaMKII in Patients with Hyperventilation Syndrome and Its Correlation with Anxiety and Depression. Medicine.

[72] Ortolá R., Sotos-Prieto M., Carballo-Casla A., Cabello-Plan S., Koni A., Mustieles V., García-Segura L.M., Artalejo A.R., Rodríguez-Artalejo F., García-Esquinas E. (2025). Role of Serum Brain-Derived Neurotrophic Factor as a Biomarker of Chronic Pain in Older Adults. Eur. J. Pain.

[73] Chang X., He Y., Liu Y., Fei J., Qin X., Song B., Yu Q., Shi M., Guo D., Hui L. (2024). Serum Brain Derived Neurotrophic Factor Levels and Post-Stroke Depression in Ischemic Stroke Patients. J. Affect. Disord..

[74] Qiu X., Wang H., Lan Y., Miao J., Pan C., Sun W., Li G., Wang Y., Zhao X., Zhu Z. (2022). Blood Biomarkers of Post-Stroke Depression after Minor Stroke at Three Months in Males and Females. BMC Psychiatry.

[75] Brammanathan S., Jain R., Sarkar S., Raghav R., Sagar R. (2024). Serum BDNF Levels Among Patients with Alcohol Dependence, Depression and Alcohol Dependence with Comorbid Depression—A Comparative Study. J. Psychoact. Drugs.

[76] Tschorn M., Kuhlmann S.L., Rieckmann N., Beer K., Grosse L., Arolt V., Waltenberger J., Haverkamp W., Müller-Nordhorn J., Hellweg R. (2021). Brain-Derived Neurotrophic Factor, Depressive Symptoms and Somatic Comorbidity in Patients with Coronary Heart Disease. Acta Neuropsychiatr..

[77] Jing J., Zhang M., Yin W., Ji Y., Sun L., Qu R., Li Y. (2025). Esketamine Relieves Postoperative Depression and Pain Indicators in Patients Undergoing Laparoscopic Total Hysterectomy. BMC Anesthesiol..

[78] Zhang Z.-N., Hao X.-Y., Cai C., Sun L., Zhang Z.-Y., Wang M., Wu Y.-S., Wang Y., Cao J.-B., Liu Y.-H. (2025). Effect of Esketamine on Postoperative Depression and Anxiety in Patients Undergoing Cardiac Valve Surgery: A Randomised, Placebo-Controlled, Double-Blinded Clinical Trial. Pharmacol. Res..

[79] Dai J., Lu Y., Zou Z., Wu Z. (2025). Optimizing Esketamine Administration for Postoperative Depression: A Comprehensive Study on Laparoscopic Bariatric Surgery Patients. Psychopharmacology.

[80] Cai J., Chen X., Jin Z., Chi Z., Xiong J. (2024). Effects of Adjunctive Esketamine on Depression in Elderly Patients Undergoing Hip Fracture Surgery: A Randomized Controlled Trial. BMC Anesthesiol..

[81] Jiang M., Wang M.-H., Wang X.-B., Liu L., Wu J.-L., Yang X.-L., Liu X.-R., Zhang C.-X. (2016). Effect of Intraoperative Application of Ketamine on Postoperative Depressed Mood in Patients Undergoing Elective Orthopedic Surgery. J. Anesth..

[82] Jiang Q., Qi Y., Zhou M., Dong Y., Zheng W., Zhu L., Li Y., Zhou H., Wang L. (2024). Effect of Esketamine on Serum Neurotransmitters in Patients with Postpartum Depression: A Randomized Controlled Trial. BMC Anesthesiol..

[83] Caliman-Fontesa A.T., Leal G.C., Correia-Melo F.S., Paixão C.S., Carvalho M.S., Jesus-Nunes A.P., Vieira F., Magnavita G., Bandeira I.D., Mello R.P. (2023). Brain-Derived Neurotrophic Factor Serum Levels Following Ketamine and Esketamine Intervention for Treatment-Resistant Depression: Secondary Analysis from a Randomized Trial. Trends Psychiatry Psychother..

[84] Mishra B.R., Mohapatra D., Biswas T., Mishra A., Panigrahi S., Maiti R. (2025). Comparative Efficacy of Antidepressant Augmentation with Amantadine vs Pramipexole in Treatment-Resistant Unipolar Depression: A Randomised Controlled Trial. J. Affect. Disord..

[85] Gupta R., Gupta K., Tripathi A.K., Bhatia M.S., Gupta L.K. (2016). Effect of Mirtazapine Treatment on Serum Levels of Brain-Derived Neurotrophic Factor and Tumor Necrosis Factor-α in Patients of Major Depressive Disorder with Severe Depression. Pharmacology.

[86] Martinotti G., Pettorruso M., De Berardis D., Varasano P.A., Lucidi Pressanti G., De Remigis V., Valchera A., Ricci V., Di Nicola M., Janiri L. (2016). Agomelatine Increases BDNF Serum Levels in Depressed Patients in Correlation with the Improvement of Depressive Symptoms. Int. J. Neuropsychopharmacol..

[87] Gupta K., Gupta R., Bhatia M.S., Tripathi A.K., Gupta L.K. (2017). Effect of Agomelatine and Fluoxetine on HAM-D Score, Serum Brain-Derived Neurotrophic Factor, and Tumor Necrosis Factor—**α** Level in Patients with Major Depressive Disorder with Severe Depression. J. Clin. Pharmacol..

[88] Navarro M.L., Breum A.W., Ozenne B., Nasser A., Aripaka S.S., Armand S., Jorgensen M.B., Frokjaer V.G., Knudsen G.M. (2025). Whole Blood BDNF Is Lower in Patients with Depression and Unchanged by Escitalopram in Patients and Healthy Controls. Prog. Neuropsychopharmacol. Biol. Psychiatry.

[89] Wu Z., Chen H., Li L., Huang Y., Lan Q., Zhu H., Luo S. (2025). Effects of Sertraline on Depressive Symptoms, Serum Brain-Derived Neurotrophic Factor (BDNF), 5-HT, and Inflammatory Cytokine Expression in Pediatric Depression Patients. Exp. Clin. Psychopharmacol..

[90] Shamabadi A., Karimi H., Fallahzadeh M.A., Vaseghi S., Arabzadeh Bahri R., Fallahpour B., Abdolghaffari A.H., Akhondzadeh S. (2025). Sex-Controlled Differences in Sertraline and Citalopram Efficacies in Major Depressive Disorder: A Randomized, Double-Blind Trial. Int. Clin. Psychopharmacol..

[91] Rengasamy M., Panny B., Hutchinson Z., Marsland A., Kovats T., Griffo A., Spotts C., Howland R.H., Wallace M.L., Mathew S.J. (2025). Lack of Relationships between Ketamine Treatment and Peripheral Neurotrophic and Inflammatory Factors in a Randomized Controlled Ketamine Trial of Major Depressive Disorder. Brain. Behav. Immun..

[92] Chen M.-H., Lin W.-C., Tsai S.-J., Li C.-T., Cheng C.-M., Wu H.-J., Bai Y.-M., Hong C.-J., Tu P.-C., Su T.-P. (2021). Effects of Treatment Refractoriness and Brain-Derived Neurotrophic Factor Val66Met Polymorphism on Antidepressant Response to Low-Dose Ketamine Infusion. Eur. Arch. Psychiatry Clin. Neurosci..

[93] Kang H.-J., Bae K.-Y., Kim S.-W., Shin I.-S., Hong Y.J., Ahn Y., Jeong M.H., Yoon J.-S., Kim J.-M. (2016). BDNF Val66met Polymorphism and Depressive Disorders in Patients with Acute Coronary Syndrome. J. Affect. Disord..

[94] Maji S., Mishra A., Mohapatra D., Mishra B.R., Jena M., Srinivasan A., Maiti R. (2024). Early Augmentation Therapy with Dextromethorphan in Mild to Moderate Major Depressive Disorder: A Group Sequential, Response Adaptive Randomized Controlled Trial. Psychiatry Res..

[95] Padhan M., Mohapatra D., Mishra B.R., Maiti R., Jena M. (2024). Efficacy and Safety of Add-on Sarcosine in Patients with Major Depressive Disorder: A Randomized Controlled Trial. J. Psychiatr. Res..

[96] Merza Mohammad T.A., Merza Mohammad T.A., Salman D.M., Jaafar H.M. (2024). Pentoxifylline as a Novel Add-on Therapy for Major Depressive Disorder in Adult Patients: A Randomized, Double-Blind, Placebo-Controlled Trial. Pharmacopsychiatry.

[97] Esalatmanesh S., Kashani L., Khooshideh M., Moghaddam H.S., Ansari S., Akhondzadeh S. (2023). Efficacy and Safety of Celecoxib for Treatment of Mild to Moderate Postpartum Depression: A Randomized, Double-Blind, Placebo-Controlled Trial. Arch. Gynecol. Obstet..

[98] Biswas T., Mishra B.R., Maiti R., Padhy S.K., Mishra A. (2024). Efficacy and Safety of Low-Dose Amisulpride versus Olanzapine-Fluoxetine Combination in Post-Schizophrenic Depression: A Randomized Controlled Trial. J. Psychiatr. Res..

[99] De Souza D.C., Marchini K.B., Nunhes P.M., Domingues W.J.R., Bertolini D.A., Oliveira V., Mazzardo O., Avelar A. (2025). Resistance Training Improves Cognitive Function and Depression Without Changing BDNF Levels in People Living with HIV: A Randomized Controlled Clinical Trial. AIDS Behav..

[100] Reed J.L., Terada T., Cotie L.M., Tulloch H.E., Leenen F.H., Mistura M., Hans H., Wang H.-W., Vidal-Almela S., Reid R.D. (2022). The Effects of High-Intensity Interval Training, Nordic Walking and Moderate-to-Vigorous Intensity Continuous Training on Functional Capacity, Depression and Quality of Life in Patients with Coronary Artery Disease Enrolled in Cardiac Rehabilitation: A Randomized Controlled Trial (CRX Study). Prog. Cardiovasc. Dis..

[101] Donyaei A., Kiani E., Bahrololoum H., Moser O. (2024). Effect of Combined Aerobic–Resistance Training and Subsequent Detraining on Brain-derived Neurotrophic Factor (BDNF) and Depression in Women with Type 2 Diabetes Mellitus: A Randomized Controlled Trial. Diabet. Med..

[102] Ribeiro V.G.C., Lacerda A.C.R., Santos J.M., Coelho-Oliveira A.C., Fonseca S.F., Prates A.C.N., Flor J., Garcia B.C.C., Tossige-Gomes R., Leite H.R. (2021). Efficacy of Whole-Body Vibration Training on Brain-Derived Neurotrophic Factor, Clinical and Functional Outcomes, and Quality of Life in Women with Fibromyalgia Syndrome: A Randomized Controlled Trial. J. Healthc. Eng..

[103] Deus L.A., Corrêa H.D.L., Neves R.V.P., Reis A.L., Honorato F.S., Silva V.L., Souza M.K., De Araújo T.B., De Gusmão Alves L.S., Sousa C.V. (2021). Are Resistance Training-Induced BDNF in Hemodialysis Patients Associated with Depressive Symptoms, Quality of Life, Antioxidant Capacity, and Muscle Strength? An Insight for the Muscle–Brain–Renal Axis. Int. J. Environ. Res. Public. Health.

[104] Hola V., Polanska H., Jandova T., Jaklová Dytrtová J., Weinerova J., Steffl M., Kramperova V., Dadova K., Durkalec-Michalski K., Bartos A. (2024). The Effect of Two Somatic-Based Practices Dance and Martial Arts on Irisin, BDNF Levels and Cognitive and Physical Fitness in Older Adults: A Randomized Control Trial. Clin. Interv. Aging.

[105] Palumbo A., Aluru V., Battaglia J., Geller D., Turry A., Ross M., Cristian A., Balagula C., Ogedegbe G., Khatri L. (2022). Music Upper Limb Therapy–Integrated Provides a Feasible Enriched Environment and Reduces Post-Stroke Depression: A Pilot Randomized Controlled Trial. Am. J. Phys. Med. Rehabil..

[106] Cartmel B., Hughes M., Ercolano E.A., Gottlieb L., Li F., Zhou Y., Harrigan M., Ligibel J.A., Von Gruenigen V.E., Gogoi R. (2021). Randomized Trial of Exercise on Depressive Symptomatology and Brain Derived Neurotrophic Factor (BDNF) in Ovarian Cancer Survivors: The Women’s Activity and Lifestyle Study in Connecticut (WALC). Gynecol. Oncol..

[107] Tibaes J.R.B., Fagundes G.B., Martins L.B., Rodrigues A.M.D.S., Campos A.C., De Souza Cordeiro L.M., Teixeira A.L., Ferreira A.V.M. (2025). Effects of a Single 10-Hour Daytime Fasting Intervention on Mood and Appetite in Female Adults with and without Obesity: A Real-World Feasibility Trial. Nutr. Neurosci..

[108] Putranto R., Setiati S., Nasrun M.W., Witjaksono F., Immanuel S., Subekti I., Harimurti K., Siswanto A., Shatri H., Suwarto S. (2024). Effects of Cholecalciferol Supplementation on Depressive Symptoms, C-Peptide, Serotonin, and Neurotrophin-3 in Type 2 Diabetes Mellitus: A Double-Blind, Randomized, Placebo-Controlled Trial. Narra J..

[109] Abiri B., Sarbakhsh P., Vafa M. (2022). Randomized Study of the Effects of Vitamin D and/or Magnesium Supplementation on Mood, Serum Levels of BDNF, Inflammation, and SIRT1 in Obese Women with Mild to Moderate Depressive Symptoms. Nutr. Neurosci..

[110] Afsharfar M., Shahraki M., Shakiba M., Asbaghi O., Dashipour A. (2021). The Effects of Magnesium Supplementation on Serum Level of Brain Derived Neurotrophic Factor (BDNF) and Depression Status in Patients with Depression. Clin. Nutr. ESPEN.

[111] Yosaee S., Soltani S., Esteghamati A., Motevalian S.A., Tehrani-Doost M., Clark C.C.T., Jazayeri S. (2020). Effects of Zinc, Vitamin D, and Their Co-Supplementation on Mood, Serum Cortisol, and Brain-Derived Neurotrophic Factor in Patients with Obesity and Mild to Moderate Depressive Symptoms: A Phase II, 12-Wk, 2 × 2 Factorial Design, Double-Blind, Randomized, Placebo-Controlled Trial. Nutrition.

[112] Pandit M., Behl T., Sachdeva M., Arora S. (2020). Role of brain derived neurotropic factor in obesity. Biomed. Pharmacother..

[113] Ashtary-Larky D., Rezaeyeh A.P., Hajizadeh L., Salarpour A., Shouhani Z., Alipour M. (2026). Effects of Dietary Weight Loss on Brain-Derived Neurotrophic Factor in Individuals with Overweight and Obesity: A Systematic Review. Nutr. Rev..

[114] Hajiluian G., Karegar S.J., Shidfar F., Aryaeian N., Salehi M., Lotfi T., Farhangnia P., Heshmati J., Delbandi A.-A. (2023). The Effects of Ellagic Acid Supplementation on Neurotrophic, Inflammation, and Oxidative Stress Factors, and Indoleamine 2, 3-Dioxygenase Gene Expression in Multiple Sclerosis Patients with Mild to Moderate Depressive Symptoms: A Randomized, Triple-Blind, Placebo-Controlled Trial. Phytomedicine.

[115] Rahimlou M., Hosseini S.A., Majdinasab N., Haghighizadeh M.H., Husain D. (2022). Effects of Long-Term Administration of Multi-Strain Probiotic on Circulating Levels of BDNF, NGF, IL-6 and Mental Health in Patients with Multiple Sclerosis: A Randomized, Double-Blind, Placebo-Controlled Trial. Nutr. Neurosci..

[116] Foshati S., Ghanizadeh A., Akhlaghi M. (2022). Extra-Virgin Olive Oil Improves Depression Symptoms Without Affecting Salivary Cortisol and Brain-Derived Neurotrophic Factor in Patients with Major Depression: A Double-Blind Randomized Controlled Trial. J. Acad. Nutr. Diet..

[117] Palmer A.C.S., Zortea M., Souza A., Santos V., Biazús J.V., Torres I.L.S., Fregni F., Caumo W. (2020). Clinical Impact of Melatonin on Breast Cancer Patients Undergoing Chemotherapy; Effects on Cognition, Sleep and Depressive Symptoms: A Randomized, Double-Blind, Placebo-Controlled Trial. PLoS ONE.

[118] Pan F., Mou T., Shao J., Chen H., Tao S., Wang L., Jiang C., Zhao M., Wang Z., Hu S. (2023). Effects of Neuronavigation-Guided rTMS on Serum BDNF, TrkB and VGF Levels in Depressive Patients with Suicidal Ideation. J. Affect. Disord..

[119] Cheng C.-M., Hong C.-J., Lin H.-C., Chu P.-J., Chen M.-H., Tu P.-C., Bai Y.-M., Chang W.-H., Juan C.-H., Lin W.-C. (2022). Predictive Roles of Brain-Derived Neurotrophic Factor Val66Met Polymorphism on Antidepressant Efficacy of Different Forms of Prefrontal Brain Stimulation Monotherapy: A Randomized, Double-Blind, Sham-Controlled Study. J. Affect. Disord..

[120] Brunoni A.R., Carracedo A., Amigo O.M., Pellicer A.L., Talib L., Carvalho A.F., Lotufo P.A., Benseñor I.M., Gattaz W., Cappi C. (2020). Association of BDNF, HTR2A, TPH1, SLC6A4, and COMT Polymorphisms with tDCS and Escitalopram Efficacy: Ancillary Analysis of a Double-Blind, Placebo-Controlled Trial. Braz. J. Psychiatry.

[121] Loo C.K., Husain M.M., McDonald W.M., Aaronson S., O’Reardon J.P., Alonzo A., Weickert C.S., Martin D.M., McClintock S.M., Mohan A. (2018). International Randomized-Controlled Trial of Transcranial Direct Current Stimulation in Depression. Brain Stimul..

[122] Liu W., Yuan J., Wu Y., Xu L., Wang X., Meng J., Wei Y., Zhang Y., Kang C.-Y., Yang J.-Z. (2024). A Randomized Controlled Trial of Mindfulness-Based Cognitive Therapy for Major Depressive Disorder in Undergraduate Students: Dose-Response Effect, Inflammatory Markers and BDNF. Psychiatry Res..

[123] Nery S.F., Paiva S.P.C., Vieira É.L., Barbosa A.B., Sant’Anna E.M., Casalechi M., Dela Cruz C., Teixeira A.L., Reis F.M. (2019). Mindfulness-based Program for Stress Reduction in Infertile Women: Randomized Controlled Trial. Stress Health.

[124] Ozkan B.N., Bozali K., Boylu M.E., Velioglu H.A., Aktas S., Kirpinar I., Guler E.M. (2024). Altered Blood Parameters in “Major Depression” Patients Receiving Repetitive Transcranial Magnetic Stimulation (rTMS) Therapy: A Randomized Case-Control Study. Transl. Psychiatry.

[125] Wang X., Fan X., Zhang L., Liu X., Ji Z. (2023). Repetitive Transcranial Magnetic Stimulation in the Treatment of Middle-Aged and Elderly Major Depressive Disorder: A Randomized Controlled Trial. Medicine.

[126] Pu Z., Hou Q., Yan H., Lin Y., Guo Z. (2023). Efficacy of Repetitive Transcranial Magnetic Stimulation and Agomelatine on Sleep Quality and Biomarkers of Adult Patients with Mild to Moderate Depressive Disorder. J. Affect. Disord..

[127] Pavlova E.L., Menshikova A.A., Semenov R.V., Bocharnikova E.N., Gotovtseva G.N., Druzhkova T.A., Gersamia A.G., Gudkova A.A., Guekht A.B. (2018). Transcranial Direct Current Stimulation of 20- and 30-Minutes Combined with Sertraline for the Treatment of Depression. Prog. Neuropsychopharmacol. Biol. Psychiatry.

[128] Dilinur A., Wang Y., Zeng F.-C., Huang Y.-W., Zhang A.-J., Hu Z.-H. (2024). Study on the Mechanisms of Acupuncture Combined with Paroxetine in the Treatment of Mild to Moderate Depression Based on DNA Methylation Analysis. Acupunct. Res..

[129] Wang P., Zhang C., Lv Q., Bao C., Sun H., Ma G., Fang Y., Yi Z., Cai W. (2018). Association of DNA Methylation in BDNF with Escitalopram Treatment Response in Depressed Chinese Han Patients. Eur. J. Clin. Pharmacol..

[130] Liu Y., Chen C., Du H., Xue M., Zhu N. (2024). Impact of Baduanjin Exercise Combined with Rational Emotive Behavior Therapy on Sleep and Mood in Patients with Poststroke Depression: A Randomized Controlled Trial. Medicine.

[131] Guo H., Ren Y., Huang B., Wang J., Yang X., Wang Y. (2022). Psychological Status, Compliance, Serum Brain-Derived Neurotrophic Factor, and Nerve Growth Factor Levels of Patients with Depression after Augmented Mindfulness-Based Cognitive Therapy. Genet. Res..

[132] Peters R.B., Xavier J., Mondin T.C., Cardoso T.D.A., Ferreira F.B., Teixeira L., Gräeff K., Quevedo L.D.A., Jansen K., Souza L.D. (2021). BDNF Val66Met Polymorphism and Resilience in Major Depressive Disorder: The Impact of Cognitive Psychotherapy. Braz. J. Psychiatry.

[133] Su T.-P., Chen M.-H., Li C.-T., Lin W.-C., Hong C.-J., Gueorguieva R., Tu P.-C., Bai Y.-M., Cheng C.-M., Krystal J.H. (2017). Dose-Related Effects of Adjunctive Ketamine in Taiwanese Patients with Treatment-Resistant Depression. Neuropsychopharmacology.

[134] Dotson V.M., Hsu F.C., Langaee T.Y., Mcdonough C.W., King A.C., Cohen R.A., Newman A.B., Kritchevsky S.B., Myers V., Manini T.M. (2016). Genetic Moderators of the Impact of Physical Activity on Depressive Symptoms. J. Frailty Aging.

[135] Ning H., Zhou H., Yang N., Ren J., Wang H., Liu W., Zhao Y. (2023). Effect of Zishen Pingchan Granules Combined with Pramipexole on Serum BDNF, IL-1β, IL-6, CRP, TNF-α Levels in Depressed Patients with Parkinson’s Disease: Results of a Randomized, Double-Blind, Controlled Study. Exp. Gerontol..

[136] Vaghef-Mehrabani E., Harouni R., Behrooz M., Ranjbar F., Asghari-Jafarabadi M., Ebrahimi-Mameghani M. (2023). Effects of Inulin Supplementation on Inflammatory Biomarkers and Clinical Symptoms of Women with Obesity and Depression on a Calorie-Restricted Diet: A Randomised Controlled Clinical Trial. Br. J. Nutr..

[137] Paduchová Z., Katrenčíková B., Vaváková M., Laubertová L., Nagyová Z., Garaiova I., Ďuračková Z., Trebatická J. (2021). The Effect of Omega-3 Fatty Acids on Thromboxane, Brain-Derived Neurotrophic Factor, Homocysteine, and Vitamin D in Depressive Children and Adolescents: Randomized Controlled Trial. Nutrients.

[138] Hasebe K., Gray L., Bortolasci C., Panizzutti B., Mohebbi M., Kidnapillai S., Spolding B., Walder K., Berk M., Malhi G. (2017). Adjunctive *N*-Acetylcysteine in Depression: Exploration of Interleukin-6, C-Reactive Protein and Brain-Derived Neurotrophic Factor. Acta Neuropsychiatr..

[139] Szuhany K.L., Otto M.W. (2020). Assessing BDNF as a Mediator of the Effects of Exercise on Depression. J. Psychiatr. Res..

[140] Kerling A., Kück M., Tegtbur U., Grams L., Weber-Spickschen S., Hanke A., Stubbs B., Kahl K.G. (2017). Exercise Increases Serum Brain-Derived Neurotrophic Factor in Patients with Major Depressive Disorder. J. Affect. Disord..

[141] Farajnia S., Rajabi H., Ghaffari M., Beladi-Moghadam N., Fayazmilani R. (2025). Impact of Cognitive-Aerobic Exercise Training on Brain-Derived Neurotrophic Factor, Dual-Tasking Abilities, and Mood State in Individuals with Multiple Sclerosis. Physiol. Behav..

[142] Nierenberg A.A., Agustini B., Köhler-Forsberg O., Cusin C., Katz D., Sylvia L.G., Peters A., Berk M. (2023). Diagnosis and Treatment of Bipolar Disorder: A Review. JAMA.

[143] Yang B., Ren Q., Zhang J., Chen Q.-X., Hashimoto K. (2017). Altered Expression of BDNF, BDNF pro-Peptide and Their Precursor proBDNF in Brain and Liver Tissues from Psychiatric Disorders: Rethinking the Brain–Liver Axis. Transl. Psychiatry.

[144] Soares A.T., Andreazza A.C., Rej S., Rajji T.K., Gildengers A.G., Lafer B., Young L.T., Mulsant B.H. (2016). Decreased Brain-Derived Neurotrophic Factor in Older Adults with Bipolar Disorder. Am. J. Geriatr. Psychiatry Off. J. Am. Assoc. Geriatr. Psychiatry.

[145] Gholipour D., Shahraki M., Shakiba M., Shamsi-Goushki A. (2025). Supplementation of Omega-3 Increases Serum Levels of Brain-Derived Neurotrophic Factor and Decreases Depression Status in Patients with Bipolar Disorder: A Randomized, Double-Blind, Placebo-Controlled Clinical Trial. J. Hum. Nutr. Diet..

[146] Zinkow A., Grodzicki W., Czerwińska M., Dziendzikowska K. (2024). Molecular Mechanisms Linking Omega-3 Fatty Acids and the Gut–Brain Axis. Molecules.

[147] Huang C.-C., Tzeng N.-S., Chang Y.-H., Hong J.-S., Lu R.-B. (2026). Neuroprotective, Cognitive, and Immunomodulatory Effects of Valproic Acid Combined with Dextromethorphan in Bipolar Disorder. J. Psychiatr. Res..

[148] Kauer-Sant’Anna M., Frey B.N., Fijtman A., Loredo-Souza A.C., Dargél A.A., Pfaffenseller B., Wollenhaupt-Aguiar B., Gazalle F.K., Colpo G.D., Passos I.C. (2019). Adjunctive Tianeptine Treatment for Bipolar Disorder: A 24-Week Randomized, Placebo-Controlled, Maintenance Trial. J. Psychopharmacol..

[149] Panizzutti B., Bortolasci C., Hasebe K., Kidnapillai S., Gray L., Walder K., Berk M., Mohebbi M., Dodd S., Gama C. (2018). Mediator Effects of Parameters of Inflammation and Neurogenesis from a *N*-Acetyl Cysteine Clinical-Trial for Bipolar Depression. Acta Neuropsychiatr..

[150] Lee S., Wang T., Chen S., Chang Y., Chen P., Huang S., Tzeng N., Wang L., Lee I.H., Chen K.C. (2018). Add-On Memantine Treatment for Bipolar II Disorder Comorbid with Alcohol Dependence: A 12-Week Follow-Up Study. Alcohol. Clin. Exp. Res..

[151] Lee S.-Y., Wang T.-Y., Chen S.-L., Chang Y.-H., Chen P.-S., Huang S.-Y., Tzeng N.-S., Wang L.-J., Lee I.H., Chen K.C. (2016). The Correlation between Plasma Brain-Derived Neurotrophic Factor and Cognitive Function in Bipolar Disorder Is Modulated by the BDNF Val66Met Polymorphism. Sci. Rep..

[152] Wang T.-Y., Lee S.-Y., Chen S.-L., Chang Y.-H., Wang L.-J., Chen P.S., Chen S.-H., Chu C.-H., Huang S.-Y., Tzeng N.-S. (2016). Comparing Clinical Responses and the Biomarkers of BDNF and Cytokines between Subthreshold Bipolar Disorder and Bipolar II Disorder. Sci. Rep..

[153] Airaksinen M.S., Saarma M. (2002). The GDNF Family: Signalling, Biological Functions and Therapeutic Value. Nat. Rev. Neurosci..

[154] Idemoto K., Niitsu T., Hata T., Ishima T., Yoshida S., Hattori K., Horai T., Otsuka I., Yamamori H., Toda S. (2021). Serum Levels of Glial Cell Line-Derived Neurotrophic Factor as a Biomarker for Mood Disorders and Lithium Response. Psychiatry Res..

[155] Wang P., Wang T., Wang Y., Li H., Qiu J., Hu J., Zhu S. (2026). The Influence of Mindfulness Therapy on NSSI and Serum proBDNF in Adolescents with Depressive Episode of Depressive Episode of Bipolar Disorder. Psychiatry Res..

[156] Wu Y., Li X., Ji X., Ren W., Zhu Y., Chen Z., Du X. (2025). Trends in the Epidemiology of Anxiety Disorders from 1990 to 2021: A Global, Regional, and National Analysis with a Focus on the Sociodemographic Index. J. Affect. Disord..

[157] Park S.-C., Kim Y.-K., Kim Y.-K. (2020). Anxiety Disorders in the DSM-5: Changes, Controversies, and Future Directions. Anxiety Disorders.

[158] Musumeci G., Minichiello L. (2011). BDNF-TrkB Signalling in Fear Learning: From Genetics to Neural Networks. revneuro.

[159] Rolfzen M.L., Nagele P., Conway C., Gibbons R., Bartels K. (2024). Management of Depression and Anxiety in Perioperative Medicine. Anesthesiology.

[160] Bi X., Dai J., Li J. (2025). The Effect of Esketamine on Postoperative Anxiety and Depression in Patients with Thyroid Cancer: A Randomized, Double-Blind, Placebo-Controlled, Parallel-Group Trial. Medicine.

[161] Luo T., Deng Z., Ren Q., Mu F., Zhang Y., Wang H. (2024). Effects of Esketamine on Postoperative Negative Emotions and Early Cognitive Disorders in Patients Undergoing Non-Cardiac Thoracic Surgery: A Randomized Controlled Trial. J. Clin. Anesth..

[162] Sıvrıkaya E.C., Yılmaz O., Tuzuner T., Korkmaz Y.T., Alver A., Arıkan S.M., Kocak N., Sahın E. (2024). Is Serum BDNF Level Relıable Parameter in Detectıng of Dental Anxıety before Impacted Thırd Molar Surgery?. Med. Oral Patol. Oral Cir. Bucal.

[163] Liu L., Yang W., Lu Y., Wang J., Zheng Y., Gu S. (2023). Clinical Efficacy of Tandospirone on Functional Dyspepsia Patients with Anxiety: A Randomized, Placebo-Controlled Study. Dig. Dis. Sci..

[164] Glue P., Neehoff S., Sabadel A., Broughton L., Le Nedelec M., Shadli S., McNaughton N., Medlicott N.J. (2020). Effects of Ketamine in Patients with Treatment-Refractory Generalized Anxiety and Social Anxiety Disorders: Exploratory Double-Blind Psychoactive-Controlled Replication Study. J. Psychopharmacol..

[165] Wang L., Yuan W., Hou H., Qi G., Xu G., Liu Z., Yang Y., Gou W., Yang Q., Yu J. (2025). Effect of Upper-Limb Robot-Assisted Therapy Combined with Pneumatic Gloves on Upper Limb Function in Young and Middle-Aged Stroke Patients: A Pilot Randomized Controlled Trial. J. Neuroeng. Rehabil..

[166] Phillips K.E., Jimerson D.C., Pillai A., Wolfe B.E. (2016). Plasma BDNF Levels Following Weight Recovery in Anorexia Nervosa. Physiol. Behav..

[167] Cavanah A.M., Robinson L.A., Aguilar M.M., Molaison E.F., Greene M.W., Roberts M.D., Fruge A.D. (2026). A Randomized Controlled Trial to Determine the Effects of Curcumin and Epigallocatechin-3-Gallate Supplementation on Serum Brain-Derived Neurotrophic Factor and Mood Disturbance in Adults. Nutrients.

[168] Rode J., Hutchinson A.N., Chatzopoulou M.S., Bleiel S.B., Gebresenbet R.F., Andersson L., Persson J., Daillère R., Beitz B., Ben Abdallah B. (2025). Micro-Encapsulation Differentially Impacts Probiotic Effects on Brain Structure and Function in an Elderly Population—A Randomised Placebo-Controlled Trial. Brain. Behav. Immun..

[169] Climent E., Hevilla F., Padial M., Barril-Cuadrado G., Blanca M., Jiménez-Salcedo T., López-Picasso M., Nogueira-Pérez Á., Olveira G. (2025). Psychobiotic Protection of Nutritional Supplements and Probiotics in Patients Undergoing Hemodialysis: A Randomized Trial. Nutrients.

[170] Haghighat N., Rajabi S., Mohammadshahi M. (2021). Effect of Synbiotic and Probiotic Supplementation on Serum Brain-Derived Neurotrophic Factor Level, Depression and Anxiety Symptoms in Hemodialysis Patients: A Randomized, Double-Blinded, Clinical Trial. Nutr. Neurosci..

[171] Pinto-Sanchez M.I., Hall G.B., Ghajar K., Nardelli A., Bolino C., Lau J.T., Martin F.-P., Cominetti O., Welsh C., Rieder A. (2017). Probiotic Bifidobacterium Longum NCC3001 Reduces Depression Scores and Alters Brain Activity: A Pilot Study in Patients with Irritable Bowel Syndrome. Gastroenterology.

[172] Mohammadi H., Karimifar M., Heidari Z., Zare M., Amani R. (2022). The Effects of Wheat Germ Consumption on Mental Health and Brain-Derived Neurotrophic Factor in Subjects with Type 2 Diabetes Mellitus: A Randomized, Double-Blind, Placebo-Controlled Trial. Nutr. Neurosci..

[173] Ma H., Wang Y., Xue Y., Huang D., Kong Y., Zhao X., Zhang M. (2019). The Effect of Xinkeshu Tablets on Depression and Anxiety Symptoms in Patients with Coronary Artery Disease: Results from a Double-Blind, Randomized, Placebo-Controlled Study. Biomed. Pharmacother..

[174] Kessler C.S., Stange R., Schlenkermann M., Jeitler M., Michalsen A., Selle A., Raucci F., Steckhan N. (2018). A Nonrandomized Controlled Clinical Pilot Trial on 8 Wk of Intermittent Fasting (24 h/Wk). Nutrition.

[175] De Oliveira B.H., Lins E.F., Kunde N.F., Salgado A.S.I., Martins L.M., Bobinski F., Vieira W.F., Cassano P., Quialheiro A., Martins D.F. (2024). Transcranial Photobiomodulation Increases Cognition and Serum BDNF Levels in Adults over 50 Years: A Randomized, Double-Blind, Placebo-Controlled Trial. J. Photochem. Photobiol. B.

[176] Xiao L., Correll C.U., Feng L., Xiang Y.-T., Feng Y., Hu C.-Q., Li R., Wang G. (2019). Rhythmic Low-Field Magnetic Stimulation May Improve Depression by Increasing Brain-Derived Neurotrophic Factor. CNS Spectr..

[177] Lu R., Zhang C., Liu Y., Wang L., Chen X., Zhou X. (2018). The Effect of Bilateral Low-Frequency rTMS over Dorsolateral Prefrontal Cortex on Serum Brain-Derived Neurotropic Factor and Serotonin in Patients with Generalized Anxiety Disorder. Neurosci. Lett..

[178] Roh H.-T., So W.-Y. (2017). Cranial Electrotherapy Stimulation Affects Mood State but Not Levels of Peripheral Neurotrophic Factors or Hypothalamic- Pituitary-Adrenal Axis Regulation. Technol. Health Care.

[179] Wang Y., Liu C., Wang Z., Li Y., Jiang H., Zhang Y., Xie Y. (2024). Internet + Wearable Device Training Effects on Limb Function Recovery and Serum Neurocytokine Content in Stroke Patients. NeuroRehabilitation.

[180] Han Z., Wang Y., Qi L., Wang J., Wong J., Chen J., Luo X., Wang Q.M. (2020). Differential Association of Serum BDNF with Poststroke Depression and Poststroke Anxiety. Arch. Phys. Med. Rehabil..

[181] Glazachev O.S., Zapara M.A., Dudnik E.N., Samartseva V.G., Susta D. (2020). Repeated Hyperthermia Exposure Increases Circulating Brain Derived Neurotrophic Factor Levels Which Is Associated with Improved Quality of Life, and Reduced Anxiety: A Randomized Controlled Trial. J. Therm. Biol..

[182] Bartlett D.M., Dominguez J.F.D., Lazar A.S., Kordsachia C.C., Rankin T.J., Lo J., Govus A.D., Power B.D., Lampit A., Eastwood P.R. (2020). Multidisciplinary Rehabilitation Reduces Hypothalamic Grey Matter Volume Loss in Individuals with Preclinical Huntington’s Disease: A Nine-Month Pilot Study. J. Neurol. Sci..

[183] González-Castro T.B., Pool-García S., Tovilla-Zárate C.A., Juárez-Rojop I.E., López-Narváez M.L., Frésan A., Genis-Mendoza A.D., Pérez-Hernández N., Nicolini H. (2019). Association between BDNF Val66Met Polymorphism and Generalized Anxiety Disorder and Clinical Characteristics in a Mexican Population: A Case–Control Study. Medicine.

[184] Anwar A., Mustafa A.M., Abdou K., Rabie M.A., El-Shiekh R.A., El-Dessouki A.M. (2025). A Comprehensive Review on Schizophrenia: Epidemiology, Pathogenesis, Diagnosis, Conventional Treatments, and Proposed Natural Compounds Used for Management. Naunyn. Schmiedebergs Arch. Pharmacol..

[185] Zhang M. (2025). Therapeutic Efficacy of Quetiapine Combined with Magnesium Valproate in Schizophrenia: Impact on Serum BDNF and GFAP Levels. Medicine.

[186] Mohammadi A., Amooeian V.G., Rashidi E. (2018). Dysfunction in Brain-Derived Neurotrophic Factor Signaling Pathway and Susceptibility to Schizophrenia, Parkinson’s and Alzheimer’s Diseases. Curr. Gene Ther..

[187] Heimfarth L., Passos F.R.S., Monteiro B.S., Araújo A.A.D.S., Quintans Júnior L.J., Quintans J.D.S.S. (2022). Serum Glial Fibrillary Acidic Protein Is a Body Fluid Biomarker: A Valuable Prognostic for Neurological Disease—A Systematic Review. Int. Immunopharmacol..

[188] Jena M., Ranjan R., Mishra B.R., Mishra A., Nath S., Sahu P., Meher B.R., Srinivasan A., Maiti R. (2019). Corrigendum to “Effect of Lurasidone vs. Olanzapine on Neurotrophic Biomarkers in Unmedicated Schizophrenia: A Randomized Controlled Trial”. J. Psychiatr. Res..

[189] Wu R.-Q., Lin C.-G., Zhang W., Lin X.-D., Chen X.-S., Chen C., Zhang L.-J., Huang Z.-Y., Chen G.-D., Xu D.-L. (2018). Effects of Risperidone and Paliperidone on Brain-Derived Neurotrophic Factor and N400 in First-Episode Schizophrenia. Chin. Med. J..

[190] Krivoy A., Hochman E., Sendt K.-V., Hollander S., Vilner Y., Selakovic M., Weizman A., Taler M. (2018). Association between Serum Levels of Glutamate and Neurotrophic Factors and Response to Clozapine Treatment. Schizophr. Res..

[191] Wu Z., Liu Q., Zhang Y., Guan X., Xiu M., Zhang X. (2022). Superoxide Dismutase, BDNF, and Cognitive Improvement in Drug-Naive First-Episode Patients with Schizophrenia: A 12-Week Longitudinal Study. Int. J. Neuropsychopharmacol..

[192] Pillai A., Schooler N.R., Peter D., Looney S.W., Goff D.C., Kopelowicz A., Lauriello J., Manschreck T., Mendelowitz A., Miller D.D. (2018). Predicting Relapse in Schizophrenia: Is BDNF a Plausible Biological Marker?. Schizophr. Res..

[193] George A.B., Gupta A., Jain R., Sood M., Sarkar S. (2024). Serum Brain-Derived Neurotrophic Factor Level and Its Relation with Cannabis Use Disorder and Schizophrenia: A Cross-Sectional Exploratory Study in Patients at a Tertiary Care Hospital. Indian J. Pharmacol..

[194] Strzelecki D., Kałużyńska O., Wysokiński A. (2016). BDNF Serum Levels in Schizophrenic Patients during Treatment Augmentation with Sarcosine (Results of the PULSAR Study). Psychiatry Res..

[195] Bang-Kittilsen G., Engh J.A., Holst R., Holmen T.L., Bigseth T.T., Andersen E., Mordal J., Egeland J. (2022). High-Intensity Interval Training May Reduce Depressive Symptoms in Individuals with Schizophrenia, Putatively through Improved VO2max: A Randomized Controlled Trial. Front. Psychiatry.

[196] Bang-Kittilsen G., Egeland J., Ueland T., Andersen E., Bigseth T.T., Holmen T.L., Mordal J., Holst R., Engh J.A. (2023). The Relationship between the Brain-Derived Neurotrophic Factor and Neurocognitive Response to Physical Exercise in Individuals with Schizophrenia. Psychoneuroendocrinology.

[197] Nuechterlein K.H., McEwen S.C., Ventura J., Subotnik K.L., Turner L.R., Boucher M., Casaus L.R., Distler M.G., Hayata J.N. (2023). Aerobic Exercise Enhances Cognitive Training Effects in First-Episode Schizophrenia: Randomized Clinical Trial Demonstrates Cognitive and Functional Gains. Psychol. Med..

[198] Chung Y.-C., Cui Y., Sumiyoshi T., Kim M.-G., Lee K.-H. (2017). Associations of Fatty Acids with Cognition, Psychopathology, and Brain-Derived Neurotrophic Factor Levels in Patients with First-Episode Schizophrenia and Related Disorders Treated with Paliperidone Extended Release. J. Psychopharmacol..

[199] Mishra A., Reeta K.H., Sarangi S.C., Maiti R., Sood M. (2022). Effect of Add-on Alpha Lipoic Acid on Psychopathology in Patients with Treatment-Resistant Schizophrenia: A Pilot Randomized Double-Blind Placebo-Controlled Trial. Psychopharmacology.

[200] Salehi B., Berkay Yılmaz Y., Antika G., Boyunegmez Tumer T., Fawzi Mahomoodally M., Lobine D., Akram M., Riaz M., Capanoglu E., Sharopov F. (2019). Insights on the Use of α-Lipoic Acid for Therapeutic Purposes. Biomolecules.

[201] Zhai W., Li M., Su Z., Ji Q., Xiong Z., Zhao Y., Yang Y., Liao D., Li C., Wang C. (2023). The Effect of Repetitive Transcranial Magnetic Stimulation on the Negative Symptoms of Chronic Schizophrenia and Serum Brain-Derived Neurotrophic Factor. Psychiatr. Pol..

[202] Gao X., Ni Y., Hu W., Wang G., He X. (2025). Comparative Study about the Therapeutic Effect of cTBS and rTMS in the Treatment of Auditory Verbal Hallucinations in Schizophrenia. Postgrad. Med. J..

[203] Li J., Zhang X., Jiang J., Zhang B., Tang Y., Zhang T., Jia Y., Li Q., Xia M., Sheng J. (2022). Comparison of Electroconvulsive Therapy and Magnetic Seizure Therapy in Schizophrenia: Structural Changes/Neuroplasticity. Psychiatry Res..

[204] Mendes-Filho V.A., De Jesus D.R., Belmonte-de-Abreu P., Cachoeira C.T., Rodrigues Lobato M.I. (2016). Effects of Repetitive Transcranial Magnetic Stimulation over Supplementary Motor Area in Patients with Schizophrenia with Obsessive-Compulsive-Symptoms: A Pilot Study. Psychiatry Res..

[205] Sun Z.-L., Liu J., Guo W., Jiang T., Ma C., Li W.-B., Tang Y.-L., Ling S.-H. (2016). Serum Brain-Derived Neurotrophic Factor Levels Associate with Cognitive Improvement in Patients with Schizophrenia Treated with Electroacupuncture. Psychiatry Res..

[206] Penadés R., López-Vílchez I., Catalán R., Arias B., González-Rodríguez A., García-Rizo C., Masana G., Ruíz V., Mezquida G., Bernardo M. (2018). BDNF as a Marker of Response to Cognitive Remediation in Patients with Schizophrenia: A Randomized and Controlled Trial. Schizophr. Res..

[207] Penadés R., Almodóvar-Payá C., García-Rizo C., Ruíz V., Catalán R., Valero S., Wykes T., Fatjó-Vilas M., Arias B. (2024). Changes in BDNF Methylation Patterns after Cognitive Remediation Therapy in Schizophrenia: A Randomized and Controlled Trial. J. Psychiatr. Res..

[208] Markiewicz R., Markiewicz-Gospodarek A., Dobrowolska B., Łoza B. (2021). Improving Clinical, Cognitive, and Psychosocial Dysfunctions in Patients with Schizophrenia: A Neurofeedback Randomized Control Trial. Neural Plast..

[209] Torrico T.J., Mann S.K., Marwaha R. (2026). Posttraumatic Stress Disorder. StatPearls.

[210] Dell’Oste V., Palego L., Betti L., Fantasia S., Gravina D., Bordacchini A., Pedrinelli V., Giannaccini G., Carmassi C. (2024). Plasma and Platelet Brain-Derived Neurotrophic Factor (BDNF) Levels in Bipolar Disorder Patients with Post-Traumatic Stress Disorder (PTSD) or in a Major Depressive Episode Compared to Healthy Controls. Int. J. Mol. Sci..

[211] Jäger A., Pieper A., Priebe K., Hellweg R., Meyer K., Herrmann S., Wolfarth B., Grummt M., Ströhle A., Schoofs N. (2024). Effects of High Intensity Interval Training on Serum Brain-Derived Neurotrophic Factor in Individuals with PTSD. J. Psychiatr. Res..

[212] Crombie K.M., Sartin-Tarm A., Sellnow K., Ahrenholtz R., Lee S., Matalamaki M., Almassi N.E., Hillard C.J., Koltyn K.F., Adams T.G. (2021). Exercise-Induced Increases in Anandamide and BDNF during Extinction Consolidation Contribute to Reduced Threat Following Reinstatement: Preliminary Evidence from a Randomized Controlled Trial. Psychoneuroendocrinology.

[213] Difede J., Rothbaum B.O., Rizzo A.A., Wyka K., Spielman L., Reist C., Roy M.J., Jovanovic T., Norrholm S.D., Cukor J. (2022). Enhancing Exposure Therapy for Posttraumatic Stress Disorder (PTSD): A Randomized Clinical Trial of Virtual Reality and Imaginal Exposure with a Cognitive Enhancer. Transl. Psychiatry.

[214] Difede J., Rothbaum B.O., Rizzo A.A., Wyka K., Spielman L., Jovanovic T., Reist C., Roy M.J., Norrholm S.D., Glatt C. (2019). Enhanced Exposure Therapy for Combat-Related Posttraumatic Stress Disorder (PTSD): Study Protocol for a Randomized Controlled Trial. Contemp. Clin. Trials.

[215] Rizzo A., Skip’, Shilling R. (2017). Clinical Virtual Reality Tools to Advance the Prevention, Assessment, and Treatment of PTSD. Eur. J. Psychotraumatology.

[216] Dai W., Kaminga A.C., Wu X., Wen S.W., Tan H., Yan J., Deng J., Lai Z., Liu A. (2017). Brain-Derived Neurotropic Factor *Val66Met* Polymorphism and Posttraumatic Stress Disorder among Survivors of the 1998 Dongting Lake Flood in China. BioMed Res. Int..

[217] Hodges H., Fealko C., Soares N. (2020). Autism Spectrum Disorder: Definition, Epidemiology, Causes, and Clinical Evaluation. Transl. Pediatr..

[218] Allan N.P., Yamamoto B.Y., Kunihiro B.P., Nunokawa C.K.L., Rubas N.C., Wells R.K., Umeda L., Phankitnirundorn K., Torres A., Peres R. (2024). Ketogenic Diet Induced Shifts in the Gut Microbiome Associate with Changes to Inflammatory Cytokines and Brain-Related miRNAs in Children with Autism Spectrum Disorder. Nutrients.

[219] Lee R.W.Y., Corley M.J., Pang A., Arakaki G., Abbott L., Nishimoto M., Miyamoto R., Lee E., Yamamoto S., Maunakea A.K. (2018). A Modified Ketogenic Gluten-Free Diet with MCT Improves Behavior in Children with Autism Spectrum Disorder. Physiol. Behav..

[220] Roshanravan N., Mahdavi R., Alizadeh E., Ghavami A., Rahbar Saadat Y., Mesri Alamdari N., Alipour S., Dastouri M.R., Ostadrahimi A. (2017). The Effects of Sodium Butyrate and Inulin Supplementation on Angiotensin Signaling Pathway via Promotion of Akkermansia Muciniphila Abundance in Type 2 Diabetes; A Randomized, Double-Blind, Placebo-Controlled Trial. J. Cardiovasc. Thorac. Res..

[221] Harris S.W., Hessl D., Goodlin-Jones B., Ferranti J., Bacalman S., Barbato I., Tassone F., Hagerman P.J., Herman K., Hagerman R.J. (2008). Autism Profiles of Males with Fragile X Syndrome. Am. J. Ment. Retard..

[222] AlOlaby R.R., Sweha S.R., Silva M., Durbin-Johnson B., Yrigollen C.M., Pretto D., Hagerman R.J., Tassone F. (2017). Molecular Biomarkers Predictive of Sertraline Treatment Response in Young Children with Fragile X Syndrome. Brain Dev..

[223] Elsheikh M.S., Ashaat E.A., Ramadan A., Mohamed N.H., Elaraby N.M., El-Hariri H.M., Hashish A.F., Nashaat N.H. (2023). Efficacy of Laser Acupuncture for Children with Autism Spectrum Disorder: Clinical, Molecular, and Biochemical Study. Pediatr. Neurol..

[224] Surapaty I.A., Simadibrata C., Rejeki E.S., Mangunatmadja I. (2020). Laser Acupuncture Effects on Speech and Social Interaction in Patients with Autism Spectrum Disorder. Med. Acupunct..

[225] White R.E., Giffard R.G. (2012). MicroRNA-320 Induces Neurite Outgrowth by Targeting ARPP-1. NeuroReport.

[226] Robinson-Agramonte M.D.L.A., Michalski B., Fernández L.G., Vidal-Martinez B., Cuesta H.V., Rizo C.M., Fahnestock M. (2021). Effect of non-invasive Brain Stimulation on Behavior and Serum Brain-derived Neurotrophic Factor and Insulin-like Growth Factor-1 Levels in Autistic Patients. Drug Dev. Res..

[227] Karna B., Sankari A., Tatikonda G. (2026). Sleep Disorder. StatPearls.

[228] Kumar V., Halder S., Srivastava S., Kar R., Jain S., Almeida E.A. (2026). Sleep, BDNF, and beyond: A Comparative Study of Zolpidem and Clobazam in Insomnia Treatment. Sleep Med..

[229] Wang M., Zhang B., Zhou Y., Wang C., Zheng W., Liu W., Zhan Y., Lan X., Ning Y. (2021). Sleep Improvement Is Associated with the Antidepressant Efficacy of Repeated-Dose Ketamine and Serum BDNF Levels: A Post-Hoc Analysis. Pharmacol. Rep..

[230] Orozco-Solis R., Montellier E., Aguilar-Arnal L., Sato S., Vawter M.P., Bunney B.G., Bunney W.E., Sassone-Corsi P. (2017). A Circadian Genomic Signature Common to Ketamine and Sleep Deprivation in the Anterior Cingulate Cortex. Biol. Psychiatry.

[231] Thomas G.M., Huganir R.L. (2004). MAPK Cascade Signalling and Synaptic Plasticity. Nat. Rev. Neurosci..

[232] Deuschle M., Schredl M., Wisch C., Schilling C., Gilles M., Geisel O., Hellweg R. (2018). Serum Brain-derived Neurotrophic Factor (BDNF) in Sleep-disordered Patients: Relation to Sleep Stage N3 and Rapid Eye Movement (REM) Sleep across Diagnostic Entities. J. Sleep Res..

[233] Golmohammadi M., Attari V.E., Salimi Y., Saed L., Nachvak S.M., Samadi M. (2025). The Effect of MIND Diet on Sleep Status, Mental Health, and Serum Level of BDNF in Overweight/Obese Diabetic Women with Insomnia: A Randomized Controlled Trial. Sci. Rep..

[234] Morris M.C., Tangney C.C., Wang Y., Sacks F.M., Bennett D.A., Aggarwal N.T. (2015). MIND Diet Associated with Reduced Incidence of Alzheimer’s Disease. Alzheimers Dement..

[235] Marx W., Lane M., Hockey M., Aslam H., Berk M., Walder K., Borsini A., Firth J., Pariante C.M., Berding K. (2021). Diet and Depression: Exploring the Biological Mechanisms of Action. Mol. Psychiatry.

[236] Liou K.T., Garland S.N., Li Q.S., Sadeghi K., Green J., Autuori I., Orlow I., Mao J.J. (2021). Effects of Acupuncture versus Cognitive Behavioral Therapy on Brain-Derived Neurotrophic Factor in Cancer Survivors with Insomnia: An Exploratory Analysis. Acupunct. Med..

[237] Grassi L., Zachariae R., Caruso R., Palagini L., Campos-Ródenas R., Riba M.B., Lloyd-Williams M., Kissane D., Rodin G., McFarland D. (2023). Insomnia in Adult Patients with Cancer: ESMO Clinical Practice Guideline. ESMO Open.

[238] Lajoie A.C., Lafontaine A.-L., Kimoff R.J., Kaminska M. (2020). Obstructive Sleep Apnea in Neurodegenerative Disorders: Current Evidence in Support of Benefit from Sleep Apnea Treatment. J. Clin. Med..

[239] Kaminska M., O’Sullivan M., Mery V.P., Lafontaine A.L., Robinson A., Gros P., Martin J.G., Benedetti A., Kimoff R.J. (2022). Inflammatory Markers and BDNF in Obstructive Sleep Apnea (OSA) in Parkinson’s Disease (PD). Sleep Med..

